# Tuning the Thermochemistry and Reactivity of a Series of Cu-Based 4H^+^/4e^–^ Electron-Coupled-Proton Buffers

**DOI:** 10.1021/acs.inorgchem.4c00835

**Published:** 2024-05-09

**Authors:** Tong Wu, Ankita Puri, Yi Lin Qiu, Daniel Ye, Rajdeep Sarma, Yiwen Wang, Tomasz Kowalewski, Maxime A. Siegler, Marcel Swart, Isaac Garcia-Bosch

**Affiliations:** † Department of Chemistry, Carnegie Mellon University, Pittsburgh, Pennsylvania 15213, United States; ‡ Johns Hopkins University, Baltimore, Maryland 21218, United States; § University of Girona, Campus Montilivi (Ciències), Plaça de Sant Domènec, 17004 Girona, Spain; ⊥ ICREA, Pg. Lluís Companys 23, 08010 Barcelona, Spain

## Abstract

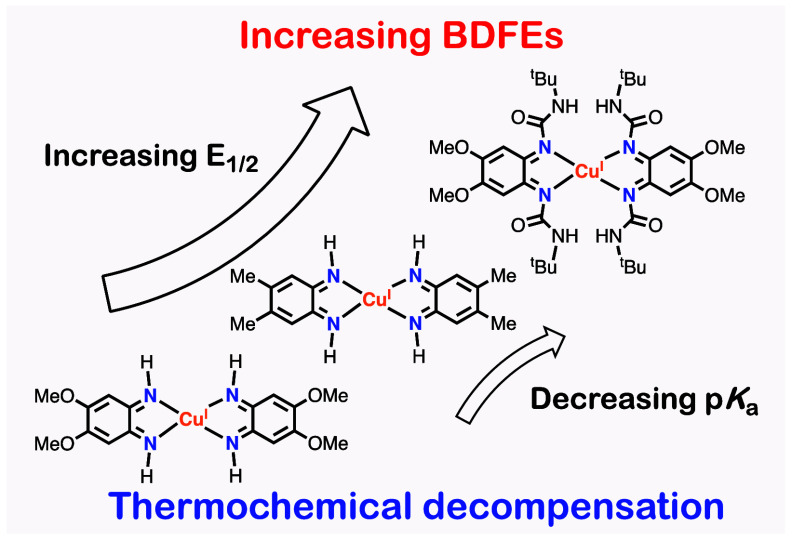

Electron-coupled-proton buffers (ECPBs) store and deliver protons and electrons in a reversible fashion. We have recently reported an ECPB based on Cu and a redox-active ligand that promoted 4H^+^/4e^–^ reversible transformations (*J. Am. Chem. Soc.***2022**, 144, 16905). Herein, we report a series of Cu-based ECPBs in which the ability of these to accept and/or donate H^•^ equivalents can be tuned via ligand modification. The thermochemistry of the 4H^+^/4e^–^ ECPB equilibrium was determined using open-circuit potential measurements. The reactivity of the ECPBs against proton-coupled electron transfer (PCET) reagents was also analyzed, and the results obtained were rationalized based on the thermochemical parameters. Experimental and computational analysis of the thermochemistry of the H^+^/e^–^ transfers involved in the 4H^+^/4e^–^ ECPB transformations found substantial differences between the stepwise (namely, BDFE_1_, BDFE_2_, BDFE_3_, and BDFE_4_) and average bond dissociation free energy values (BDFE_avg._). Our analysis suggests that this “redox unleveling” is critical to promoting the disproportionation and ligand-exchange reactions involved in the 4H^+^/4e^–^ ECPB equilibria. The difference in BDFE_avg._ within the series of Cu-based ECPBs was found to arise from a substantial change in the redox potential (*E*_1/2_) upon modification of the ligand scaffold, which is not fully compensated for by a change in the acidity/basicity (p*K*_a_), suggesting “thermochemical decompensation”.

## Abstract 2

In this paper, we show that the reactivity of Cu-based 4H^+^/4e^−^ electron-coupled-proton buffers (ECPBs) can be tuned via ligand modification. Experimental and computational studies on the thermochemistry of the H^+^/e^−^ transfers involved in the ECPB reactions indicate substantial differences between the stepwise and average bond dissociation free energy (BDFE) values of the different Cu-based ECPBs, suggesting that these systems depict “redox unleveling” and “thermochemical decompensation”.

## Introduction

**Figure 1:**
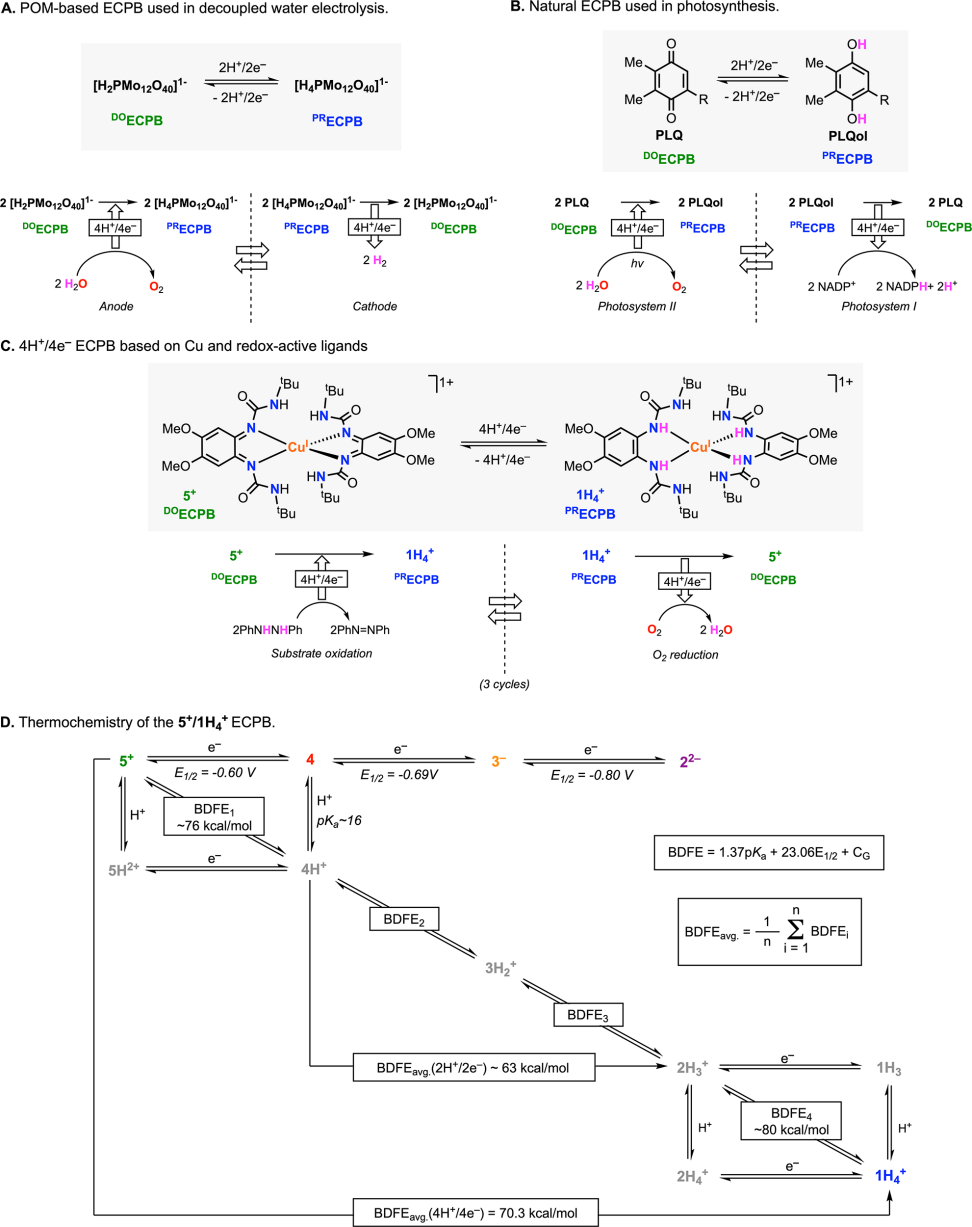
Reactivity of ECPBs based on POMs (A), natural quinones (B), and Cu complexes (C). Thermochemical analysis of the PCET reactions involved in the reactivity of the Cu 4H^+^/4e^–^ ECPB (**5**^**+**^/**1H**_**4**_^**+**^). Herein, we describe a series of Cu-based ECPBs in which the thermochemistry (*E*_1/2_, p*K*_a_, stepwise BDFEs, and BDFE_avg._) of the 4H^+^/4e^–^ equilibria can be tuned by modifying the redox-active ligand scaffold (Scheme 1).

Electron-coupled-proton buffers (ECPBs) are redox mediators capable of storing and delivering H atom equivalents (*n*H^+^ and *n*e^–^) in a reversible fashion. The ECPB term was coined by Symes and Cronin in 2013 in a research paper that described the use of polyoxometalate (POM) phosphomolybdic acid (H_3_PMo_12_O_40_) to perform the electrolysis of water in a decoupled fashion (Figure 1A).^1^ The use of the ECPB allowed us to carry out the two half-reactions at different times and space: O_2_ production during water oxidation in the anode yielded protons and electrons, which protonated and reduced the ECPB; then reoxidation of ECPB released H_2_ gas in the cathode.

**Scheme 1:**
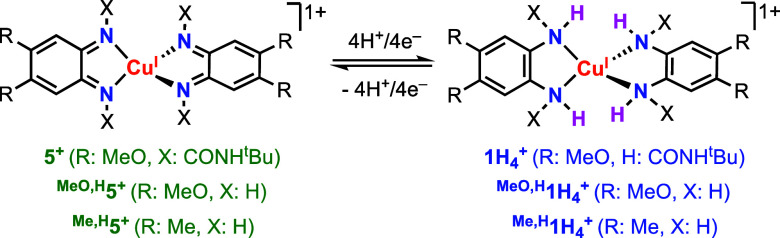
Cu-Based ECPBs Described in This Paper

Natural systems have evolved to utilize ECPBs to store, transport, and deliver protons and electrons (Figure 1B). For instance, the plastoquinone/plastoquinol ECPB couple stores the 4H^+^/4e^–^ generated in water oxidation in photosystem II and transports them to photosystem I, in which the reduction of nicotinamide adenine dinucleotide phosphate (NADP^+^) to NADPH and the production of adenosine triphosphate occurs.^2^ Inspired by these natural systems, artificial ECPBs based on water-soluble quinones (potassium 1,4-hydroquinonesulfonate and anthraquinone-2,7-disulfonic acid) have also been utilized as ECPBs in several electrochemical devices.^3−5^

We have recently reported an ECPB based on Cu and redox-active ligands that promotes reversible 4H^+^/4e^–^ transformations (Figure 1C).^6^ The ECPB capabilities of the **5**^**+**^/**1H**_**4**_^**+**^ system were demonstrated by reacting the Cu complexes with stoichiometric amounts of H^+^/e^–^ donors and acceptors [proton-coupled electron transfer (PCET), reagents], which converted **5**^**+**^ to **1H**_**4**_^**+**^ and vice versa. The Cu-based ECPB system also reacted with substoichiometric amounts of PCET reagents to shift the **5**^**+**^/**1H**_**4**_^**+**^ equilibrium. The **5**^**+**^/**1H**_**4**_^**+**^ system also allowed us to separate in time and space (i.e., decouple) substrate dehydrogenation and O_2_ protonation/reduction: complex **1H**_**4**_^**+**^ was fully transformed to complex **5**^**+**^ using O_2_ as proton–electron acceptor and after O_2_ removal, complex **5**^**+**^ reacted with 2 equiv of diphenylhydrizine (PhNHNHPh) to stoichiometrically produce **1H**_**4**_^**+**^ and azobenzene (PhN=NPh).

The thermochemistry of PCET reactions can be determined with the Bordwell equation, in which the tendency of a species to accept or donate H^•^ equivalents (bond dissociation free energy, BDFE) depends on its acidity/basicity (p*K*_a_) and its redox potential (*E*_1/2_; Figure 1D).^7^ For multiproton/multielectron events, the individual stepwise BDFE values lead to an average BDFE (BDFE_avg._) value that can be experimentally obtained by open-circuit potential (OCP) measurements, an approach recently developed by Mayer and co-workers.^8^ For the **5**^**+**^/**1H**_**4**_^**+**^ system, we reported the BDFE value for the 1H^+^/1e^–^ reduction of **5**^**+**^, the BDFE value of the 1H^+^/1e^–^ deprotonation/oxidation of **1H**_**4**_^**+**^, and the BDFE_avg._ value for the 4H^+^/4e^–^ ECPB equilibrium (Figure 1D).

## Results and Discussion

### Preparation and Characterization of Cu-Based ECPBs

**Figure 2:**
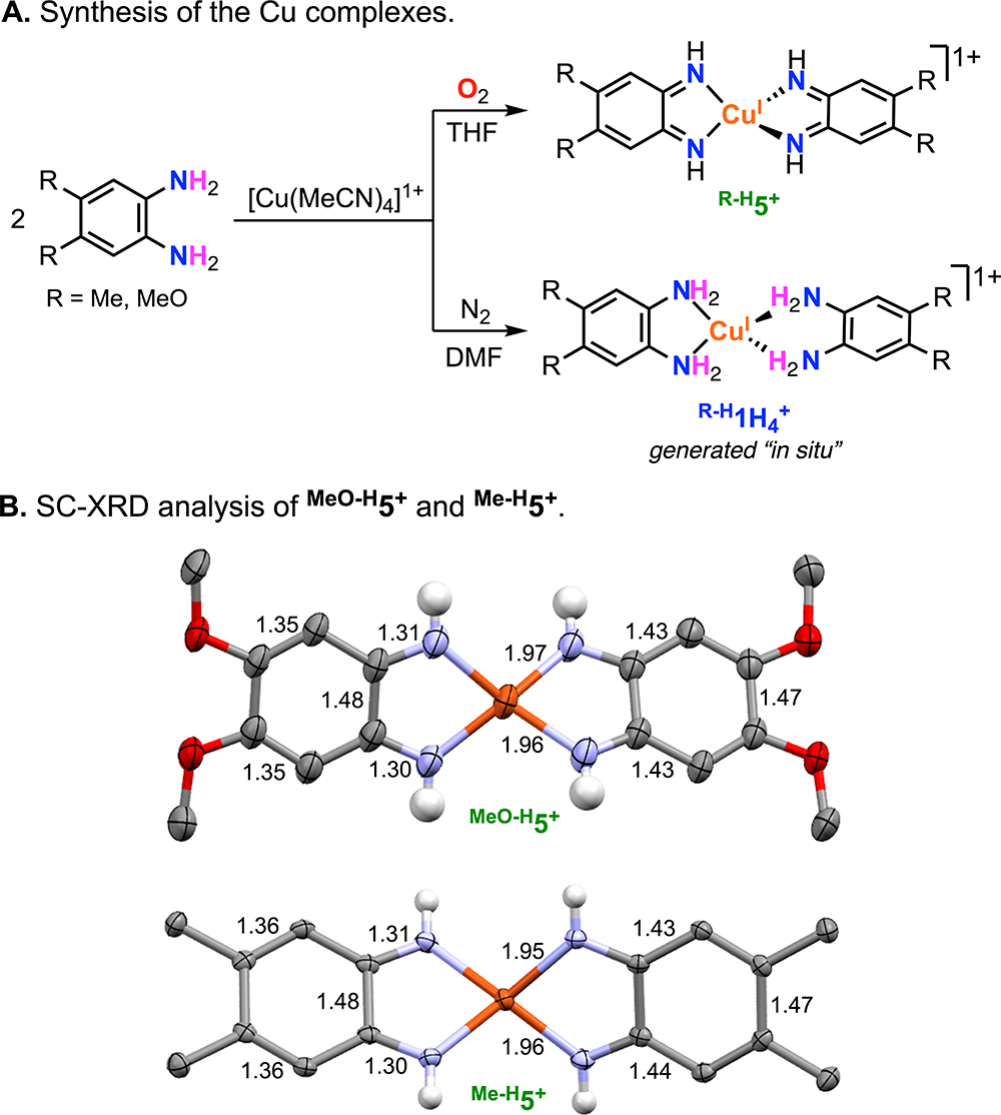
(A) Synthesis of the Cu complexes. (B) SC-XRD analysis of ^**MeO-H**^**5**^**+**^ and ^**Me-H**^**5**^**+**^. The PF_6_^–^ counteranions, lattice tetrahydrofuran solvent molecules, and most H atoms were omitted for clarity.

The deprotonated/oxidized ECPBs (^**MeO-H**^**5**^**+**^ and ^**Me-H**^**5**^**+**^) were prepared by reacting 2 equiv of the redox-active ligands (4,5-dimethoxyl-1,2-phenylenediamine or 4,5-dimethyl-1,2-phenylenediamine) and 1 equiv of [Cu^I^(MeCN)_4_]PF_6_ with O_2_ (Figure 2; see experimental details in the Supporting Information, SI). The Cu complexes, isolated as dark-blue crystals, were analyzed by ^1^H NMR (**5**^**+**^, ^**MeO-H**^**5**^**+**^, and ^**Me-H**^**5**^**+**^ are all diamagnetic species) and single-crystal X-ray diffraction (SC-XRD) analysis. The pseudotetrahedral structures of ^**MeO-H**^**5**^**+**^ and ^**Me-H**^**5**^**+**^ exhibited short C–N distances (∼1.31 Å) and aromatic C–C bonds with varying distances (two double bonds of ∼1.35 Å and four single bonds of ∼1.45 Å), supporting the formulation of these complexes as cuprous ions bound by two quinone-like ligands.^6^

Like for complex **1H**_**4**_^**+**^, the protonated/reduced ECPBs ^**MeO-H**^**1H**_**4**_^+^ and ^**Me-H**^**1H**_**4**_^**+**^ could not be isolated and were generated in situ by mixing 1 equiv of [Cu^I^(MeCN)_4_]PF_6_ with 2 equiv of the corresponding ligand under anaerobic conditions (Figure 2A). *N*,*N*-Dimethylformamide (DMF) solutions of ^**MeO-H**^**1H**_**4**_^**+**^ and ^**Me-H**^**1H**_**4**_^**+**^ underwent slow disproportionation, a process that could be precluded in the presence of ^**MeO-H**^**5**^**+**^ and ^**Me-H**^**5**^**+**^ (i.e., the protons and electrons produced during the disproportionation of ^**MeO-H**^**1H**_**4**_^**+**^ can be trapped by ^**MeO-H**^**5**^**+**^ to generate ^**MeO-H**^**4H**^**+**^, which disproportionate to regenerate ^**MeO-H**^**5**^**+**^ and ^**MeO-H**^**1H**_**4**_^**+**^; see the SI for further details). The ^1^H NMR spectra of the in situ generated ^**MeO-H**^**1H**_**4**_^**+**^ and ^**Me-H**^**1H**_**4**_^**+**^ depicted shifting and broadening of the ligand peaks compared to those of solutions containing only ligand, confirming the interaction between the Cu^I^ ion and ligand scaffolds (see the SI).

### OCP Measurements for Cu-Based ECPBs and PCET Reagents

Mayer and co-workers have recently shown that the thermochemical information of PCET couples obtained from OCP measurements is more accurate than the results obtained by traditional cyclic voltammetry (CV) in nonaqueous solvents.^8^ OCPs have been applied in analysis of the BDFE_avg._ values of PCET reagents involving multiple H^•^, including organic substrates like quinones,^8^ metal complexes,^6,8^ and POMs.^9,10^ OCPs [*E*° (V vs H_2_)] are measured at different relative concentrations of the protonated/reduced form of the PCET reagent (H_*n*_X) and deprotonated/oxidized form (X), and the resulting linear plot allows one to obtain the BDFE_avg._ [intercept: potential (*E*°, V vs H_2_) when [H_*n*_X] = [X]; BDFE = 23.06*E*° + Δ*G*(H^•^)] and the number of protons and electrons involved in the transformation (slope = −0.0592 V/*n*).

**Figure 3:**
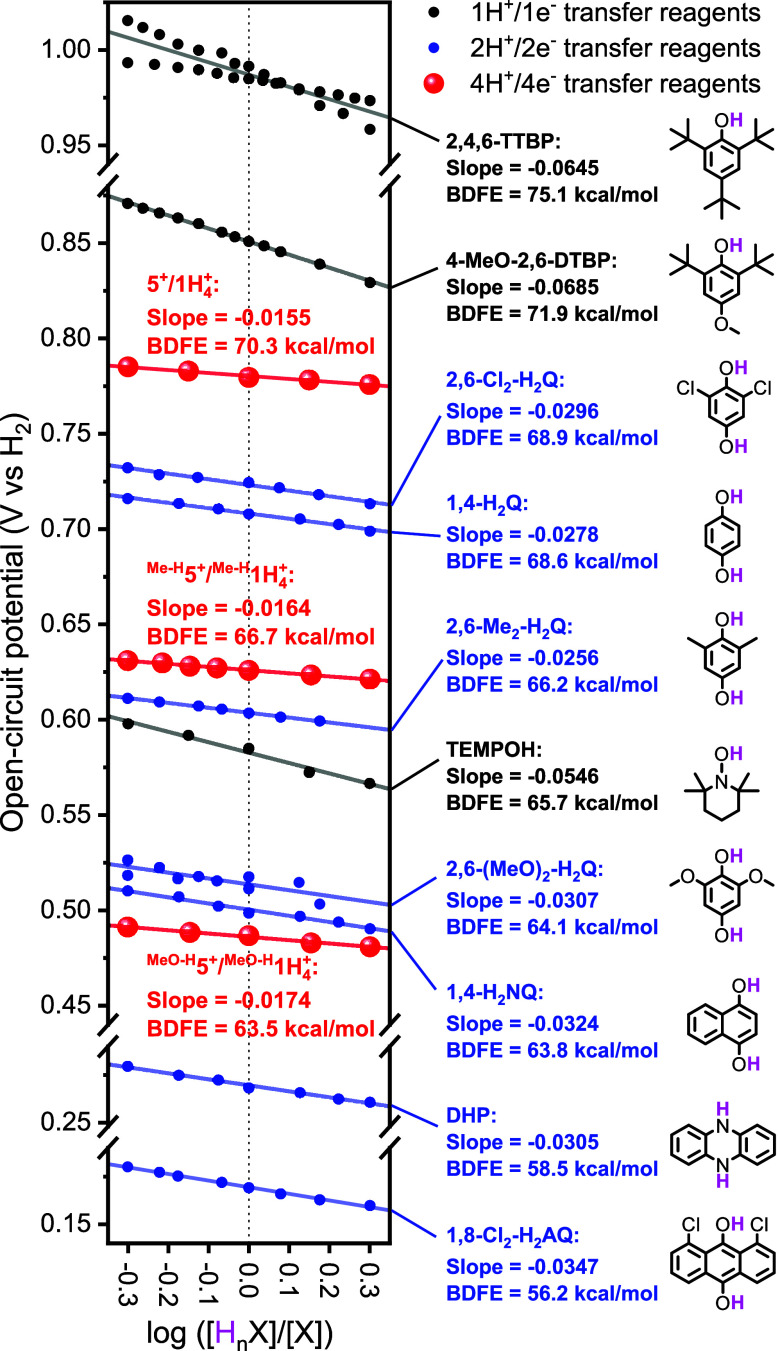
OCP measurements for the Cu-based ECPBs and PCET reagents (see the SI for experimental details).

For **5**^**+**^/**1H**_**4**_^**+**^, we reported the first example of a 4H^+^/4e^–^ PCET couple determined by OPC measurements (slope = −0.0155, with a slope of −0.0149 for the ideal 4H^+^/4e^–^ couple), with a BDFE_avg._ value of 70.3 kcal/mol [*E*° (V vs H_2_) = 0.781 V in DMF] using a triethylamine/triethylammonium buffer. We proposed that the ability of the Cu-ECPB system to undergo fast 1H^+^/1e^–^ events to regenerate the equilibrium between **5**^**+**^ and **1H**_**4**_^**+**^ allowed for determination of the BDFE_avg._ value of the 4H^+^/4e^–^ process (see further discussion in sections below). The same OCP measurements were carried out for ^**MeO-H**^**5**^**+**^/^**MeO-H**^**1H**_**4**_^**+**^ and ^**Me-H**^**5**^**+**^/^**Me-H**^**1H**_**4**_^**+**^ (Figure 3; see also the SI). Like in **5**^**+**^/**1H**_**4**_^**+**^, slopes consistent with the 4H^+^/4e^–^ processes were obtained (ca. −0.015). Interestingly, the BDFE_avg._ values for ^**MeO-H**^**5**^**+**^/^**MeO-H**^**1H**_**4**_^**+**^ and ^**Me-H**^**5**^**+**^/^**Me-H**^**1H**_**4**_^**+**^ (63.5 and 66.7 kcal/mol, respectively) were substantially lower than that for **5**^**+**^/**1H**_**4**_^**+**^ (70.3 kcal/mol), suggesting that ligand variation allows for tuning of the BDFEs of Cu-based ECPBs. Under the same conditions, we measured the OCPs for several PCET couples, including hydroquinones/quinones (2H^+^/2e^–^ couples), TEMPOH/TEMPO, and phenols/phenoxyl radicals (1H^+^/1e^–^ couples), with BDFE values ranging from 56 to 75 kcal/mol (Figure 3). A wide BDFE range from 56 to 75 kcal/mol was obtained, and the slopes of the linear plots were well in line with the H atom equivalents in each PCET couple [e.g., 4-MeO-2,6-DTBP/4-MeO-2,6-DTBP^•^, 1H^+^/1e^–^ process, slope = −0.0685, BDFE = 71.9 kcal/mol; dihydrophenazine (DHP)/phenazine, 2H^+^/2e^–^ process, slope = −0.0305, BDFE = 58.5 kcal/mol]. The reactivities between these PECT reagents and Cu-based ECPBs (**5**^**+**^, ^**MeO-H**^**5**^**+**^, and ^**Me-H**^**5**^**+**^) are described below.

### Reactivity of Cu-Based ECPBs Dependent on BDFEs

**Figure 4:**
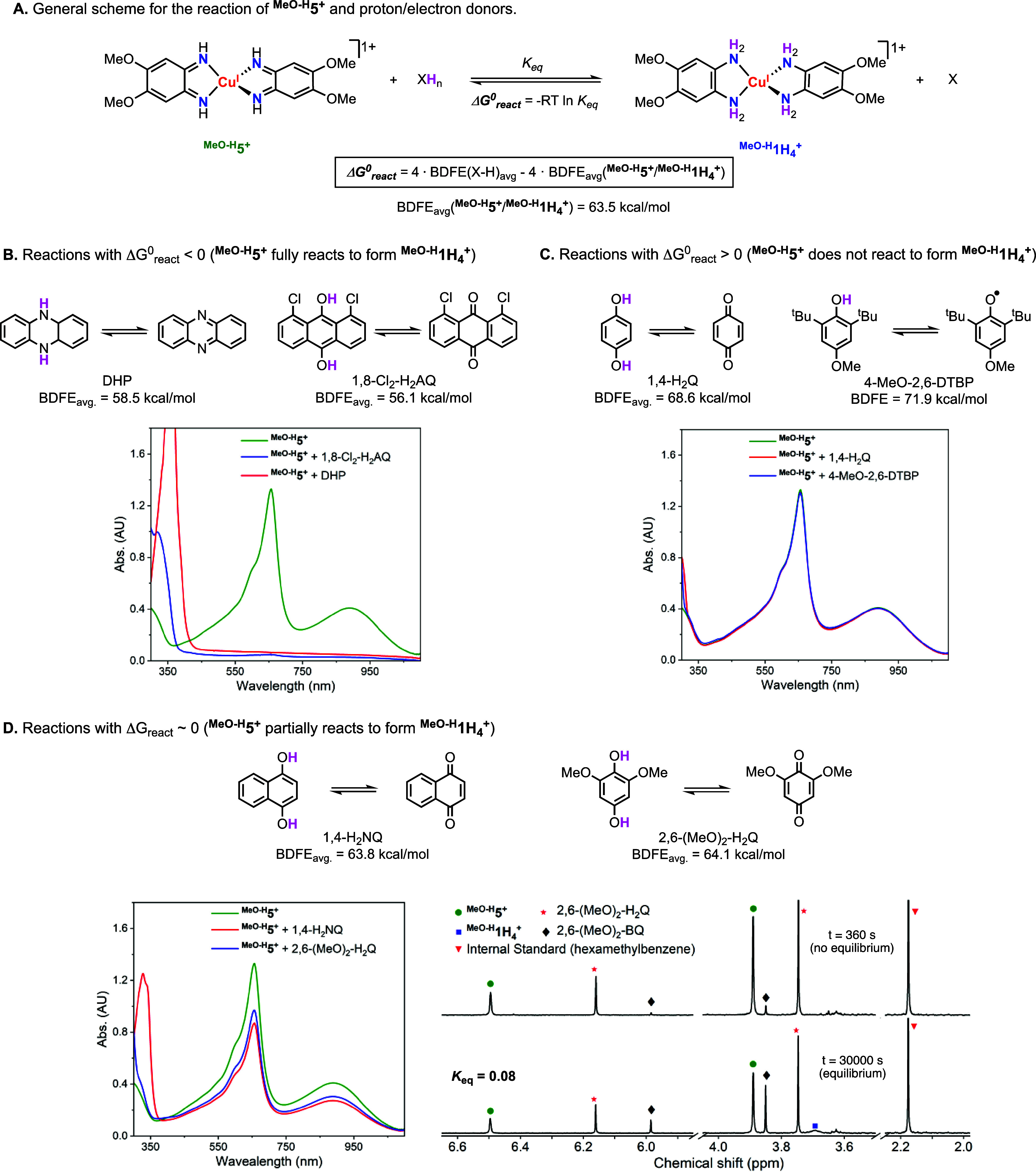
(A) General scheme for analysis of the reactivity of ^**MeO-H**^**5**^**+**^ and PCET reagents in DMF (see experimental details in the SI). (B) UV–vis spectra of the reactions between ^**MeO-H**^**5**^**+**^ and PCET reagents with X–H bonds lower than the BDFE_avg._ values of the ^**MeO-H**^**5**^**+**^/^**MeO-H**^**1H**_**4**_^**+**^ couple (Δ*G*_react_ < 0 kcal/mol; ^**MeO-H**^**5**^**+**^ fully reacts to form ^**MeO-H**^**1H**_**4**_^**+**^). (C) UV–vis spectra of reactions between ^**MeO-H**^**5**^**+**^ and X–H bonds with BDFE values higher than the BDFE_avg._ values of the ^**MeO-H**^**5**^**+**^/^**MeO-H**^**1H**_**4**_^**+**^ couple (Δ*G*_react_ < 0 kcal/mol; no reaction observed). (D) UV–vis and ^1^H NMR spectra [in the case of 2,6-(MeO)_2_-H_2_Q] of the reactions between ^**MeO-H**^**5**^**+**^ and X–H bonds with BDFE values similar to the BDFE_avg._ values of the ^**MeO-H**^**5**^**+**^/^**MeO-H**^**1H**_**4**_^**+**^ couple (Δ*G*_react_ ∼ 0 kcal/mol; ^**MeO-H**^**5**^**+**^ partially reacts to form ^**MeO-H**^**1H**_**4**_^**+**^).

The reactivity of Cu-based ECPBs with stoichiometric amounts of PCET reagents was analyzed in DMF by ^1^H NMR and UV–vis spectroscopy (Table 1; see experimental details in the SI). Based on the free energy of the PCET reactions (Δ*G*°_react_, calculated from BDFE values obtained by OCP measurements; see Figure 4A and the SI for details), the deprotonated/oxidized forms of the ECPBs (**5**^**+**^, ^**MeO-H**^**5**^**+**^, and ^**Me-H**^**5**^**+**^) should fully react with proton/electron donors with lower BDFEs (Δ*G*°_react_ < 0), should reach equilibrium with proton/electron donors with similar BDFEs (Δ*G*°_react_ ∼ 0), and should not react with proton/electron donors with higher BDFEs (Δ*G*°_react_ > 0).^11^ For example, the reaction between ^**MeO-H**^**5**^**+**^ (BDFE_avg._ = 63.5 kcal/mol) and 2 equiv of 1,8-dichlorohydroanthraquione (1,8-Cl_2_-H_2_AQ; BDFE = 56.2 kcal/mol) led to full conversion of ^**MeO-H**^**5**^**+**^ to ^**MeO-H**^**1H**_**4**_^**+**^ (Δ*G*°_react_ = −29.2 kcal/mol, see Figure 4). The reaction was followed by ^1^H NMR, and we observed the disappearance of peaks corresponding to ^**MeO-H**^**5**^**+**^ and 1,8-Cl_2_-H_2_AQ and the appearance of peaks corresponding to ^**MeO-H**^**1H**_**4**_^**+**^ and 1,8-Cl_2_-H_2_AQ (see the SI). The reaction could also be analyzed by UV–vis spectroscopy, in which the intense UV–vis bands of ^**MeO-H**^**5** (intense blue color, λ_max_ = 662 nm; ε = 25100 M^–1^ cm^–1^) decayed to produced pale-yellow solutions characteristic of ^**MeO-H**^**1H**_**4**_^**+**^ and 1,8-Cl_2_-H_2_AQ (Figure 4B, left). The reaction between ^**MeO-H**^**5**^**+**^ (BDFE_avg._ = 63.5 kcal/mol) and 2 equiv of 2,6-dimethoxy-1,4-hydroquinone [2,6-(MeO)_2_-H_2_Q; BDFE_avg._ = 64.1 kcal/mol] was also followed by ^1^H NMR and UV–vis spectroscopy (Figure 4D). Because the BDFE_avg._ values of the ECPB and the PCET reagent are similar (Δ*G*°_react_ = 2.4 kcal/mol), a thermochemical equilibrium was reached. The equilibrium constant obtained by NMR [*K*_eq_ = 0.08, determined by the concentrations of ^**MeO-H**^**5**^**+**^, ^**MeO-H**^**1H**_**4**_^**+**^, 2,6-(MeO)_2_-H_2_Q, and 2,6-(MeO)_2_-BQ] was similar to the *K*_eq_ obtained by UV–vis [*K*_eq_ = 0.05, determined by comparing the UV–vis spectra before and after the addition of 2,6-(MeO)_2_-H_2_Q to ^**MeO-H**^**5**], both in close agreement with the equilibrium constant calculated from the OCP measurements (*K*_eq_ = 0.16).

**Table 1:** Summary of the Reactivity of Cu-Based ECBPs with PCET Reagents

			^**MeO-H**^**5**^**+**^ → ^**MeO-H**^**1H**_**4**_^**+**^		^**Me-H**^**5**^**+**^ → ^**Me-H**^**1H**_**4**_^**+**^		**5**^**+**^ → **1H**_**4**_^**+**^	
PCET substrate	*E*° (V vs H_2_)^a^	BDFE (kcal/mol)	reaction	Δ*G*°_react_^b^	reaction	Δ*G*°_react_^b^	reaction	Δ*G*°_react_^b^
2,4,6-TTBP	0.987	75.1	NR	46.4	NR	33.6	NR	19.2
4-MeO-2,6-DTBP	0.851	71.9	NR	33.6	NR	20.8	*K*_eq_ = 10^–6^	6.4
2,6-Cl_2_-H_2_Q	0.724	68.9	NR	21.6	NR	8.8	*K*_eq_ = 55	–5.6
1,4-H_2_Q	0.708	68.6	NR	20.4	NR	7.6	√	–6.8
2,6-Me_2_-H_2_Q	0.592	66.0	NR	10.0	*K*_eq_ = 7.0	–2.8	√	–17.2
TEMPOH	0.582	65.7	NR	8.8	*K*_eq_ = 10^4^	–4.0	√	–18.4
2,6-(MeO)_2_-H_2_Q	0.514	64.1	*K*_eq_ = 0.08	2.4	√	–10.4	√	–24.8
1,4-H_2_NQ	0.500	63.8	*K*_eq_ = 0.46	1.2	√	–11.6	√	–26.0
DHP	0.269	58.5	√	–20.0	√	–32.8	√	–47.2
1,8-Cl_2_-H_2_AQ	0.169	56.2	√	–29.2	√	–42.0	√	–56.4

a *E*° (V) vs SHE in a triethylamine/triethylammonium buffer (50 mM) in DMF.

b Δ*G*° (kcal/mol) for reactions in DMF of ^**X-R**^**5**^**+**^ + *m*XH_*n*_ → ^**X-R**^**1H**_**4**_^**+**^ + *m*X from *n*FE° or from BDFEs. NR = not react.

As predicted by OCP measurements, ^**MeO-H**^**5**^**+**^ (BDFE_avg._ for ^**MeO-H**^**5**^**+**^/^**MeO-H**^**1H**_**4**_^**+**^ = 63.5 kcal/mol) did not react with substrates with higher BDFEs such as TEMPOH (BDFE_O–H_ = 65.7 kcal/mol; Δ*G*°_react_ = 8.8 kcal/mol) and 2,6-dimethyl-1,4-dihydroquinone (2,6-Me_2_-H_2_Q; BDFE_avg._ = 66.2 kcal/mol; Δ*G*°_react_ = 10.8 kcal/mol; Figure 4C). Conversely, 2,6-Me_2_-H_2_Q was partially dehydrogenated by ^**Me-H**^**5**^**+**^ (BDFE_avg._ for ^**Me-H**^5^+^/^**Me-H**^**1H**_**4**_^**+**^ = 66.2 kcal/mol; Δ*G*°_react_ = −2.4 kcal/mol) and fully dehydrogenated by **5**^**+**^ (BDFE_avg._ for **5**^**+**^/**1H**_**4**_^**+**^ = 70.3 kcal/mol; Δ*G*°_react_ = −16.4 kcal/mol). The superior BDFE_avg._ of the **5**^**+**^/**1H**_**4**_^**+**^ couple compared to those of ^**Me-H**^**5**^**+**^/^**Me-H**^**1H**_**4**_^**+**^ and ^**MeO-H**^**5**^**+**^/^**MeO-H**^**1H**_**4**_^**+**^ was demonstrated in the reaction between the ECPBs and 2,6-dichlorohydroquinone (2,6-Cl_2_-H_2_Q; BDFE_avg._ = 68.9 kcal/mol), in which only **5**^**+**^ reacted with this PCET reagent.

### ECPB-Mediated Decoupled O_2_ Reduction and Substrate Oxidation

By definition, ECPBs capture and deliver H^•^ equivalents in a reversible and decoupled fashion.^1^ We have previously shown that the **5**^**+**^/**1H**_**4**_^**+**^ system could be used to carry out the dehydrogenation of diphenylhydrizine (2PhNHNHPh → 2PhN=NPh + 4H^•^) and the protonation/reduction of O_2_ (O_2_ + 4H^•^ → H_2_O) in a decoupled fashion (Figure 1D). We found that, after performing the three cycles of the decoupled 4H^+^/4e^–^ process (i.e., each cycle consisted of the deprotonation/oxidation of **1H**_**4**_^**+**^ to **5** with O_2_, followed by the protonation/reduction of **1H**_**4**_^**+**^ to **5** with 2 equiv of PhNHNHPh), the ECPB mass balance [ECPB mass balance (%) = (moles of **1H**_**4**_^**+**^ + **5**)/(initial moles of **1H**_**4**_^**+**^ + **5**) × 100] decayed to ∼70%.

**Figure 5:**
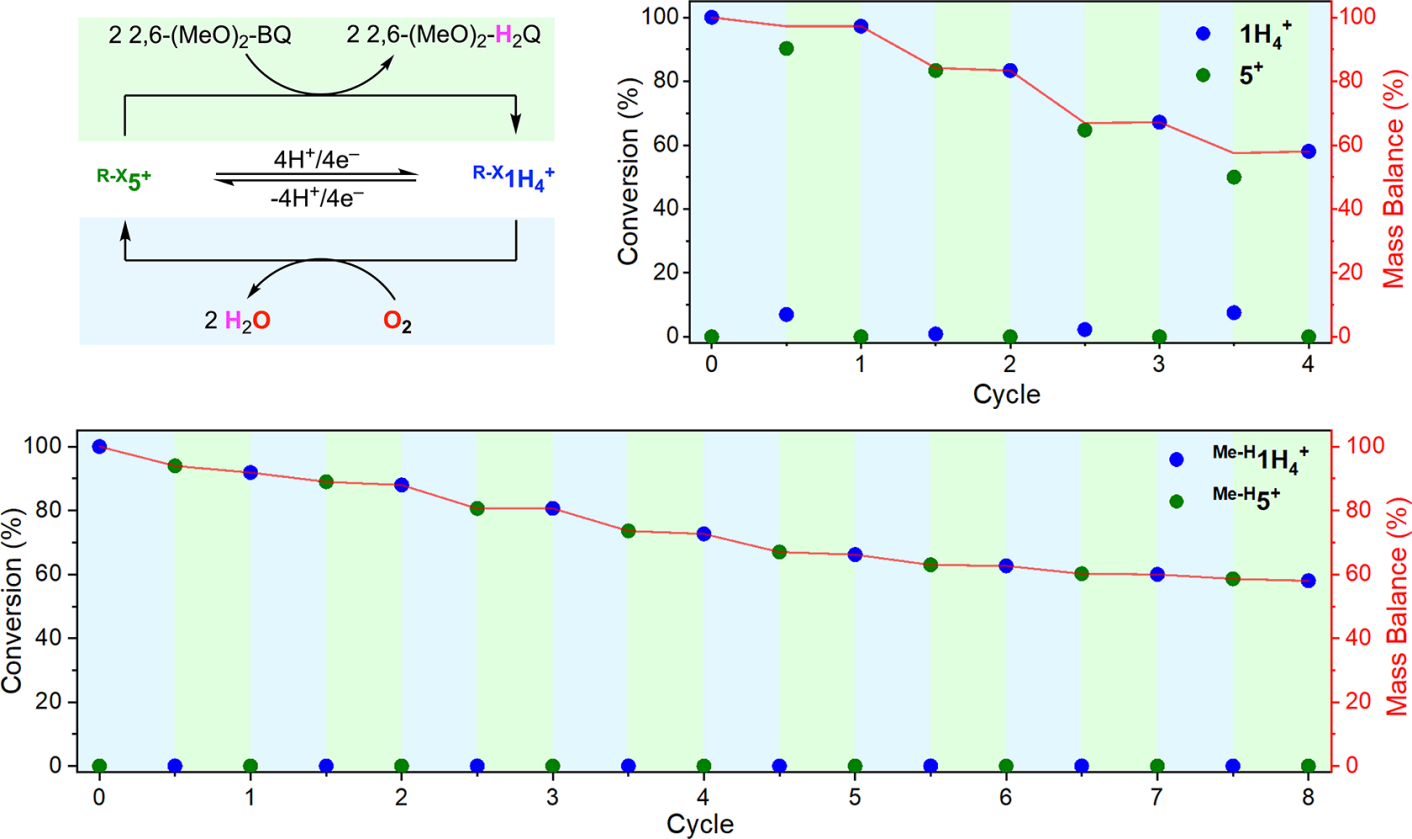
Decoupled O_2_ protonation/reduction and substrate deprotonation/oxidation [2,6-(MeO)_2_-H_2_Q] promoted by the Cu-based ECPBs ^**Me-H**^**5**^**+**^/^**Me-H**^**1H**_**4**_^**+**^ (bottom) and **5**^**+**^/**1H**_**4**_ (top right). Reactions were carried out in DMF-*d*_7_ at room temperature, followed by ^1^H NMR (see the SI for further experimental details).

For this paper, we decided to evaluate the reactivity of the series of Cu-based ECPBs in decoupled processes (Figure 5). To do so, we used **5**^**+**^/**1H**_**4**_^**+**^ and ^**Me-H**^**5**^**+**^/^**Me-H**^**1H**_**4**_^**+**^ in the decoupled protonation/reduction of O_2_ and the dehydrogenation of 2,6-(MeO)_2_-H_2_Q (BDFE_avg._ = 64.1 kcal/mol). The oxygenation of ^**PR**^**ECPB** (**1H**_**4**_^**+**^ and ^**Me-H**^**1H**_**4**_^**+**^) to produce ^**DO**^**ECPB** (**5**^**+**^ and ^**Me-H**^**5**^**+**^, respectively) was followed by ^1^H NMR (see the SI). After full consumption of ^**PR**^**ECPB**, the excess of O_2_ was removed, and 2 equiv of 2,6-(MeO)_2_-H_2_Q (i.e., 4 equiv of H^•^) was added under anaerobic conditions. The decay of ^**DO**^**ECPB** and 2,6-(MeO)_2_-H_2_Q and the formation of 2,6-(MeO)_2_-Q were quantified by NMR (see further details in the SI).

Like in our previous report, we observed that each of the decoupled cycles led to a decrease of the EPCB mass balance (Figure 5). Interestingly, we observed that, after four decoupled cycles, the mass balance for the **5**^**+**^/**1H**_**4**_^**+**^ system decreased to 60% (∼10% decrease per cycle, as previously observed), while the ^**Me-H**^**5**^**+**^/^**Me-H**^**1H**_4_^**+**^ system required eight cycles to reach the same mass balance. NMR analysis of the reactions suggests that degradation of the **5**^**+**^/**1H**_**4**_^**+**^ system occurs during the oxygenation of **1H**_**4**_^**+**^ with O_2_. The appearance of new aromatic peaks, methanol, and degradation products derived from the ureanyl substituent suggests that the redox-active diamine undergoes oxidative degradation during oxygenation (see the SI). Conversely, the oxygenation of ^**Me-H**^**1H**_**4**_^**+**^ to produce ^**Me-H**^**5**^**+**^ did not lead to any ligand decomposition products that could be detected by ^1^H NMR.

### Thermochemical Analysis of Cu-Based ECPBs

**Figure 6:**
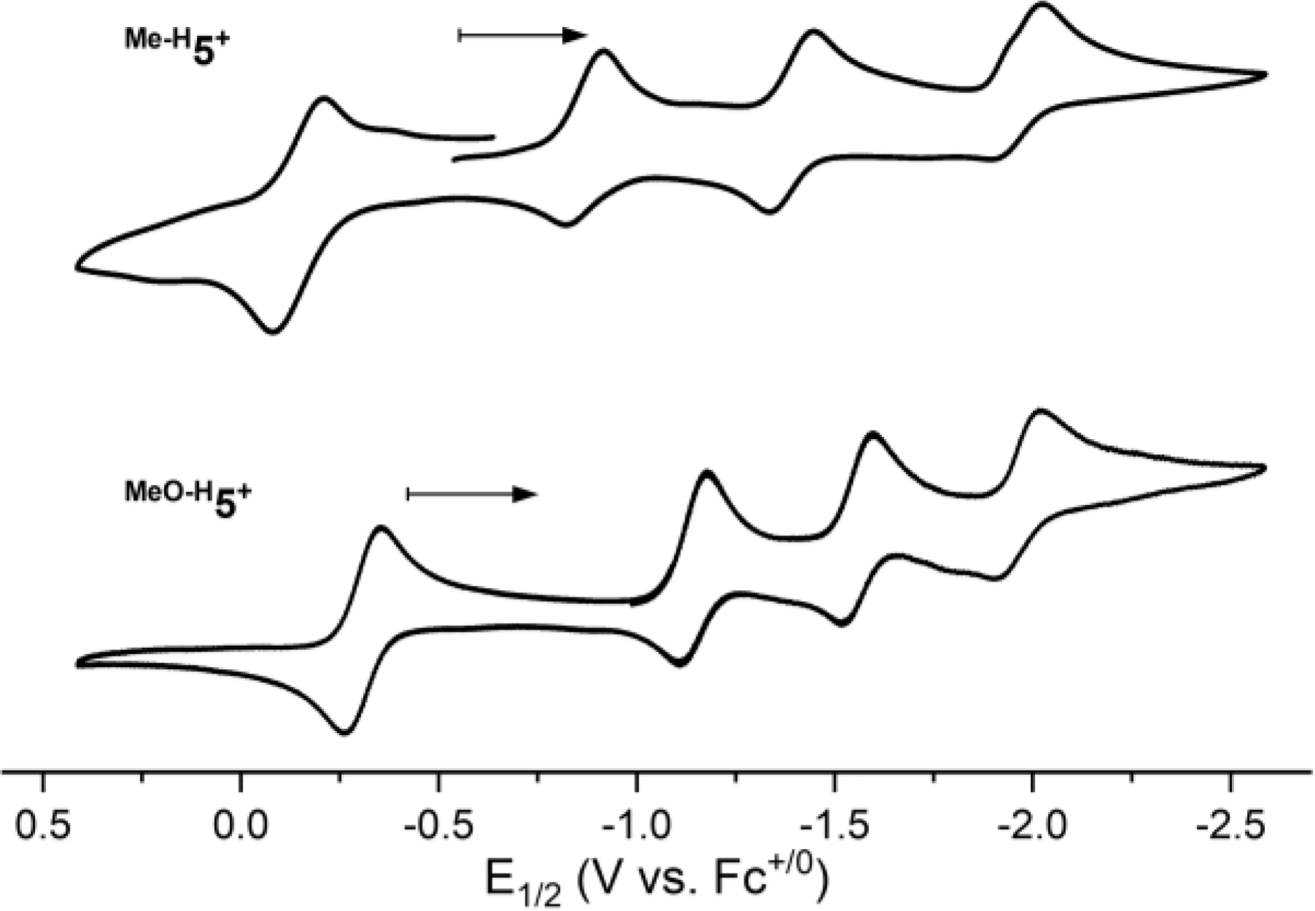
CV measurements for the reversible reduction/oxidation of complexes ^**Me-H**^**5**^**+**^ (top) and ^**MeO-H**^**5**^**+**^ (bottom).

We have previously shown that complex **5**^**+**^ can be reversibly reduced via three consecutive 1e^–^ processes to generate the complexes **4**, **3**^**–**^, and **2**^**2–**^, which were spectroscopically and computationally characterized (Figure 6).^6^ CV experiments indicated that the reduction of **5**^**+**^ to **4**, **3**^**–**^, and **2**^**2–**^ occurred at very low potentials (−0.60, −0.69, and −0.88 V vs Fc^0/+^ in DMF). CV measurements for ^**Me-H**^**5**^**+**^ and ^**MeO-H**^**5**^**+**^ exhibited shifting of the reduction events to more negative potentials (−0.87, −1.37, and −1.94 V for ^**Me-H**^**5**^**+**^; −1.18, −1.57, and −1.96 V for ^**MeO-H**^**5**^**+**^; see Figure 6, Table 2, and the SI for further details). The ability of ^**Me-H**^**5**^**+**^ and ^**MeO-H**^**5**^**+**^ to stabilize “high” oxidation states at potentials more negative than those of **5**^**+**^ also allowed one to observe the 1e^–^ oxidation of ^**Me-H**^**5**^**+**^ to ^**Me-H**^**6**^**2+**^ and ^**MeO-H**^**5**^**+**^ to ^**MeO-H**^**6**^**2+**^ at relatively negative potentials (−0.12 V for ^**Me-H**^**5**^**+**^ and −0.31 V for ^**MeO-H**^**5**^**+**^).

**Table 2:** Thermochemical Parameters of Cu-Based ECPBs

		**^MeO-H^5**^**+**^/^**MeO-H**^**1H**_**4**_^**+**^	^**Me-H**^**5**^**+**^/^**Me-H**^**1H**_**4**_^**+**^	**5**^**+**^/**1H**_**4**_^**+**^
*E*_1/2_ (V vs Fc^0/+^)	^**X-R**^**6**^**2+**^/^**X-R**^**5**^**+**^	–0.31	–0.12	
	^**X-R**^**5**^**+**^/^**X-R**^**4**	–1.18	–0.87	–0.60
	^**X-R**^**4**/^**X-R**^**3**^**–**^	–1.57	–1.37	–0.69
	^**X-R**^**3**^**–**^/^**X-R**^**2**^**2–**^	–1.96	–1.94	–0.89
p*K*_a_	^**X-R**^**4**/^**X-R**^**4H**^**+**^	∼20	∼18	∼16
BDFE (kcal/mol)	^**X-R**^**5**^**+**^/^**X-R**^**4H**^**+**^	∼68	∼72	∼76
	^**X-R**^**2H**_**3**_^**+**^/^**X-R**^**1H**_**4**_^**+**^	n.d.	n.d.	∼80
	^**X-R**^**5**^**+**^/^**X-R**^**1H**_**4**_^**+**^	63.5	66.7	70.3

**Scheme 2:**
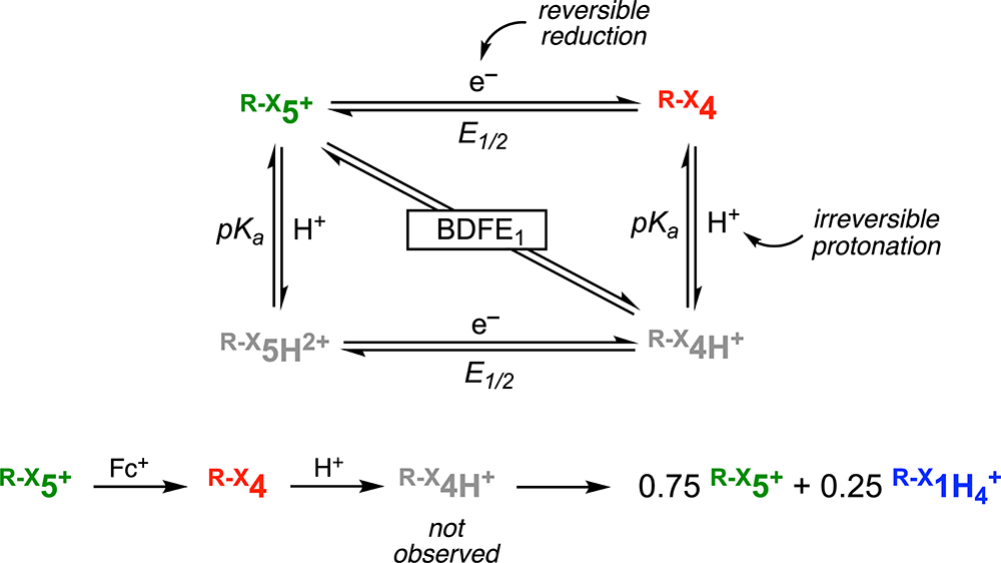
Determination of the BDFEs for the ^**R-X**^**5**^**+**^/^**R-X**^**1H**_**4**_^**+**^ Couples

In our previous report, we showed that the BDFE for the 1H^+^/1e^–^ conversion of **5**^**+**^ to **4H**^+^ was experimentally determined via the stoichiometric reduction of **5**^**+**^ to **4** and the subsequent protonation of **4** to **4H**^**+**^ (Scheme 2). The protonation of **4** to **4H**^**+**^ was irreversible (**4H**^**+**^ disproportionated to produce 0.75 equiv of **5**^**+**^ and 0.25 equiv of **1H**_**4**_^**+**^), which led to an approximate determination of the p*K*_a_ value (∼16). The BDFE of the **5**^**+**^/**4H**^**+**^ couple (obtained from the Bordwell equation with the *E*_1/2_ of the **5**^**+**^/**4** couple and the p*K*_a_ of the **4**/**4H**^**+**^ couple) was higher than the BDFE_avg._ of the 4H^+^/4e^–^ conversion of **5**^**+**^ to **1H**_**4**_^**+**^ (76 vs 70 kcal/mol). The reduction of ^**Me-H**^**5**^**+**^ and ^**MeO-H**^**5**^**+**^ to ^**Me-H**^**4** and ^**MeO-H**^**4** was carried out using stoichiometric amounts of cobaltocene. Like in complex **4**, the protonation of ^**Me-H**^**4** and ^**MeO-H**^**4** also led to disproportionation to produce the corresponding ECBP mixtures (i.e., ^**Me-H**^**5**^**+**^/^**Me-H**^**1H**_**4**_^**+**^ and ^**MeO-H**^**5**^**+**^/^**MeO-H**^**1H**_**4**_^**+**^; see the SI). Our results indicate that acids with different strengths are needed to protonate **4**, ^**Me-H**^**4**, and ^**MeO-H**^**4**, with **4** requiring stronger acids (e.g., 3-Cl-PhOH, p*K*_a_ in DMF = 16.3) than ^**Me-H**^4 (PhOH, p*K*_a_ in DMF = 18.4) and ^**MeO-H**^**4** (diphenylurea, p*K*_a_ in DMF = 20.6; see the SI). The decreased basicity of ^**Me-H**^**4** compared to that of ^**MeO-H**^**4** is consistent with the superior donating ability of the MeO groups. The diminished basicity of **4** compared to that of ^**MeO-H**^**4** can be explained by the electron-withdrawing ability of the ureanyl substituent compared to that of H.

The BDFE for the 1H^+^/1e^–^ deprotonation/oxidation of **1H**_**4**_^**+**^ to **2H**_**3**_^**+**^ using combinations of 1e^–^ oxidants and bases was previously determined to be 80 kcal/mol, which was substantially higher than the BDFE_avg._ of the **5**^**+**^/**1H**_**4**_^**+**^ couple (76 kcal/mol). Unfortunately, the oxidative deprotonation of ^**Me-H**^**1H**_**4**_^**+**^ and ^**MeO-H**^**1H**_**4**_^**+**^ could not be carried out due to the instability of these species in solution (see the SI).

**Figure 7:**
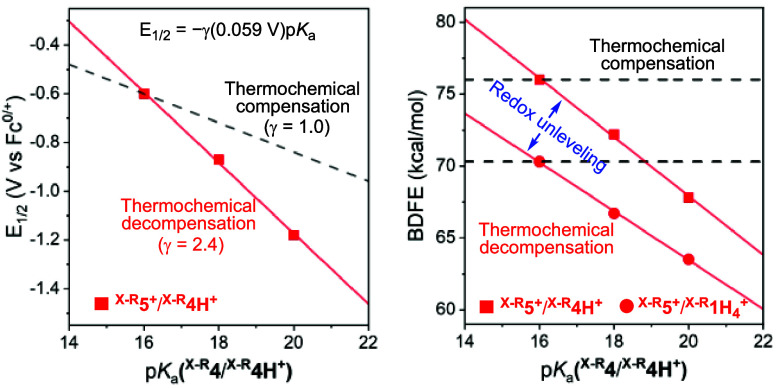
Thermochemical decompensation and redox unleveling observed for the ^**X-R**^**5**^**+**^/^**X-R**^**4H**^**+**^ and ^**X-R**^**5**^**+**^/^**X-R**^**1H**_**4**_^**+**^ couples.

For the three Cu-based ECPB systems analyzed, we observed substantial differences in the BDFEs of the ^**X-R**^**5**^**+**^/^**X-R**^**4H**^**+**^ couples, which was also observed in the BDFE_avg._ values of the ^**X-R**^**5**^**+**^/^**X-R**^**1H**_**4**_^**+**^ couples (Table 2). This effect is consistent with a modest change in p*K*_a_ of the protonation of reduced complexes ^**MeO-H**^**4**^**+**^ and ^**Me-H**^**4**^**+**^ compared to that of **4**^**+**^, a deviation smaller than what might be expected based on the change in *E*_1/2_ of the ^**X-R**^**5**^**+**^/^**X-R**^**4** couples (Figure 6). This thermochemical decompensation (BDFEs are more affected by changes in *E*_1/2_ than in p*K*_a_) has been observed and quantified in other inorganic and organic systems^12^ and explains the differences in the BDFEs of ECPB systems upon ligand modification (Figure 7).

### Mechanism of ECPB Regeneration

**Figure 8:**
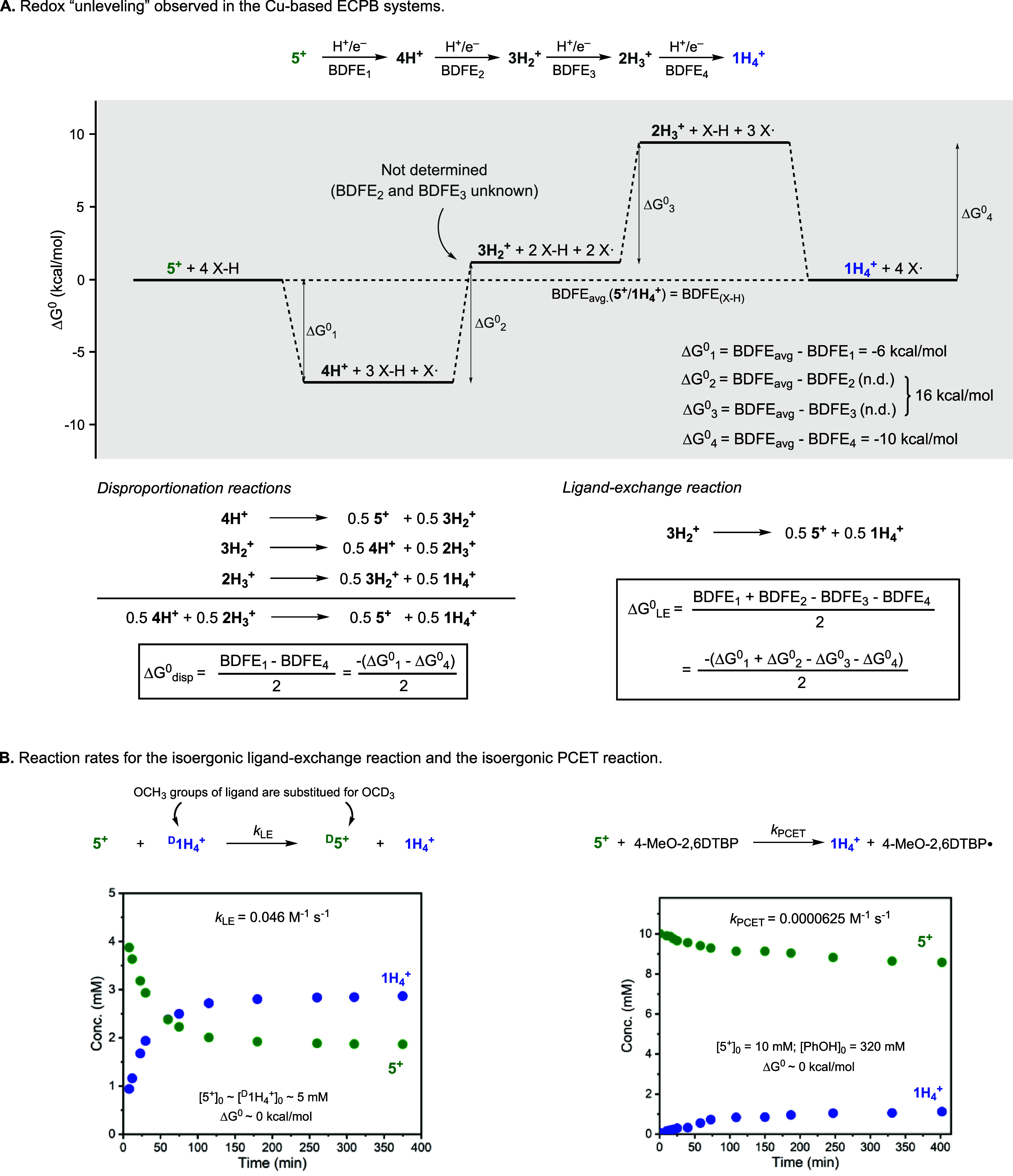
Thermochemical analysis of the disproportionation and ligand-exchange reactions involved in the **5**^**+**^/**1H**_**4**_^**+**^ ECPB (A). Comparison of the reaction rates for the ligand-exchange and PCET reactions involved in the **5**^**+**^/**1H**_**4**_^**+**^ ECPB (B).

As previously mentioned, we also observed a substantial difference between the BDFEs of the ^**X-R**^**5**^**+**^/^**X-R**^**4H**^**+**^ couples and the BDFE_avg._ values of the ^**X-R**^**5**^**+**^/^**X-R**^**1H**_**4**_^**+**^ couples (∼5 kcal/mol). We have previously hypothesized that the “redox unleveling” behavior observed in the **5**^**+**^/**1H**_**4**_^**+**^ system (i.e., difference between the BDFE_avg._ values and the stepwise BDFEs^7^) was critical for the observed reactivity, favoring the selective formation of **5**^**+**^ and **1H**_**4**_^**+**^ via disproportionation reactions (e.g., **4** → 0.5**5**^**+**^ + 0.5**3H**_**2**_^**+**^). Another process that we have not previously considered and could be involved in regeneration of the ^**X-R**^**5**^**+**^/^**X-R**^**1H**_**4**_^**+**^ couples is the ligand-exchange reaction (**3H**_**2**_^**+**^ → 0.5**5**^**+**^ + 0.5**1H**_**4**_^**+**^). The thermodynamic chemistry of the disproportionation and ligand-exchange reactions that leads to the **5**^**+**^/**1H**_**4**_^**+**^ equilibrium can be analyzed based on the BDFEs (stepwise and average) of the 4H^+^/4e^–^ conversion of **5**^**+**^ to **1H**_**4**_^**+**^ (see the isoergonic 4H^+^/4e^–^ transformation of **5**^**+**^ to **1H**_**4**_^**+**^ in Figure 8A in which **5**^**+**^ is reacted with 4 equiv of a hypothetical substrate, X–H, a species with a BDFE_(X-H)_ equal to BDFE_avg._ of the **5**^**+**^/**1H**_**4**_^**+**^ couple). Even though the BDFE values for the **4H**^**+**^/**3H**_**2**_^**+**^ and **3H**_**2**_^**+**^/**2H**_**3**_^**+**^ couples could not be experimentally determined, the BDFE_avg._ value for the **4H**^**+**^/**2H**_**3**_^**+**^ couple could be estimated (63 kcal/mol). Our analysis indicates that the overall thermodynamic driving force for the disproportionation reactions (Δ*G*°_disp_) is independent of the stepwise BDFEs of the **4H**^**+**^/**3H**_**2**_^**+**^ and **3H**_**2**_^**+**^/**2H**_**3**_^**+**^ couples, and it is favored when the BDFE of the **5**^**+**^/**4H**^**+**^ couple is lower than the BDFE of the **2H**_**3**_^**+**^/**1H**_**4**_^**+**^ couple (BDFE_1_ < BDFE_4_). In contrast, the driving force for the ligand-exchange reaction (Δ*G*°_LE_) is highly dependent on the BDFEs for the **4H**^**+**^/**3H**_**2**_^**+**^ and **3H**_**2**_^**+**^/**2H**_**3**_^**+**^ couples, and it is favored when the BDFE_avg._ value of the **5H**^**+**^/**3H**_**2**_^**+**^ couple is lower than the BDFE_avg._ of the **3H**_**2**_^**+**^/**1H**_**4**_^**+**^ couple (BDFE_1_ + BDFE_2_ < BDFE_3_ + BDFE_4_).

To gather additional evidence on the proposed disproportionation and ligand-exchange reactions that lead to fast regeneration of the Cu-based ECPB systems, we compared the rate of the ligand-exchange reaction to the rate of a PCET reaction under similar reaction conditions (Figure 8B). If our hypothesis is correct, we should observe faster reaction rates for ligand exchange compared to PCET for transformations with similar thermochemical driving forces. The ligand-exchange reaction was carried out by mixing complex **5**^**+**^ and complex ^**D**^**1H**_**4**_^**+**^ (formed by mixing Cu^I^ and 2 equiv of the OCD_3_ derivative ligand; see the SI). The reaction was followed by ^1^H NMR by monitoring the integration of the OCH_3_ peaks of **5**^**+**^ and **1H**_**4**_^**+**^ (see the spectra in the SI). After mixing equimolar amounts of **5**^**+**^ and ^**D**^**1H**_**4**_^**+**^ ([**5**^**+**^]_0_ ∼ [^**D**^**1H**^**+**^]_0_ ∼ 5 mM), we observed the decay of the OCH_3_ peaks corresponding to **5**^**+**^ and the appearance of the OCH_3_ peaks corresponding to **1H**_**4**_^**+**^. The ligand-exchange reaction (isoergonic, Δ*G*° ∼ 0 kcal/mol) reached equilibrium after ∼300 min, and the second-order rate constant was calculated using the initial rates method (*k*_LE_ = 0.046 M^–1^ s^–1^; see the SI for details). The reaction rate for a PCET reaction was obtained by reacting **5**^**+**^ and 4-MeO-2,6-DTBP. Based on BDFE considerations, the reaction between **5**^**+**^ (10 mM, BDFE_avg._ = 70.3 kcal/mol) and excess amounts of 4-MeO-2,6-DTBP (320 mM, BDFE = 71.9 kcal/mol) should reach **5**^**+**^/**1H**_**4**_^**+**^ equilibrium (isoergonic, Δ*G*° ∼ 0 kcal/mol). The reaction was also followed by ^1^H NMR by monitoring the concentrations of **5**^**+**^ and **1H**_**4**_^**+**^ (see spectra in the SI). The second-order rate constant for the PCET reaction (*k*_PCET_ = 0.0000625 M^–1^ s^–1^) was calculated using the initial rates method (see the SI), and it was significantly lower than the rate constant of the ligand-exchange reaction, reinforcing the feasibility of the proposed **5**^**+**^/**1H**_**4**_^**+**^ ECPB regeneration mechanism.

### Computational Analysis of the Electronic Structure and Thermochemistry of the Cu Species Involved in the ECPB Reactivity

**Scheme 3:**
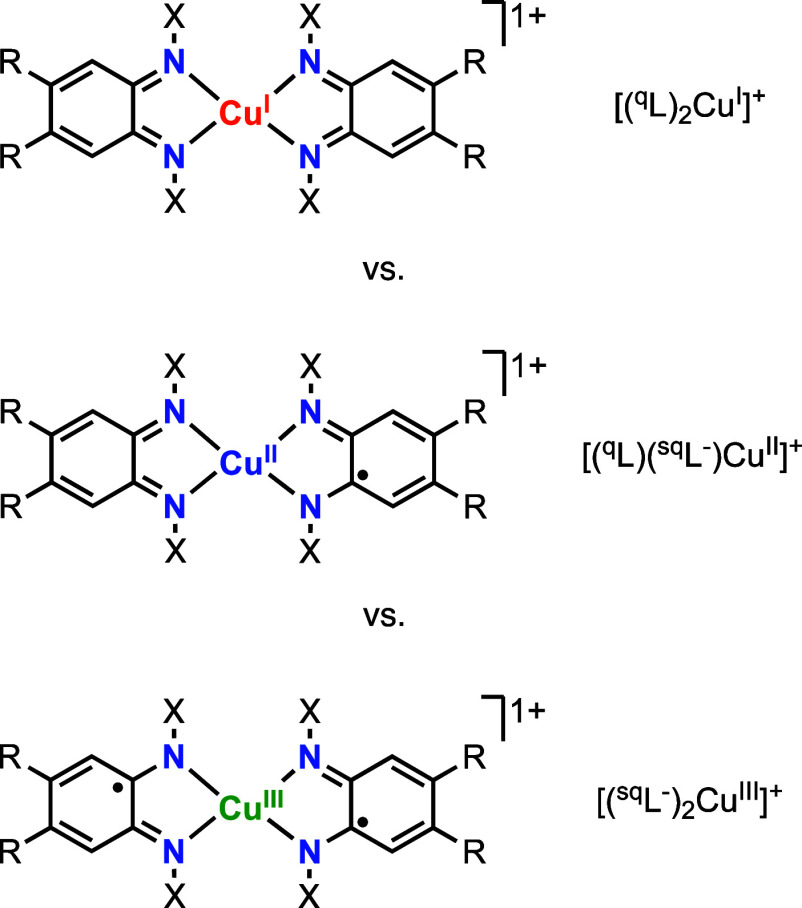
Ambiguity on the Electronic Structure of Metal Complexes Bound by Redox Non-innocent Ligands

In a landmark paper, Jørgensen stated that “ligands are innocent when they allow oxidation states of the central atom to be defined”.^13^ The author argued that some ligands are “suspect” due to the ambiguity of their electronic structure upon coordination to metal ions. In many instances, redox-active ligands are considered “non-innocent” because determining the oxidation state of the metal and the oxidation state of the ligand is not trivial.^14−17^ For example, ^**R-X**^**5**^**+**^ (which are structurally and spectroscopically described as cuprous complexes bound by quinone-like ligands) can be represented by three valence tautomers in which the Cu ion and the ligand scaffold adopt different oxidation states (Scheme 3).

**Figure 9:**
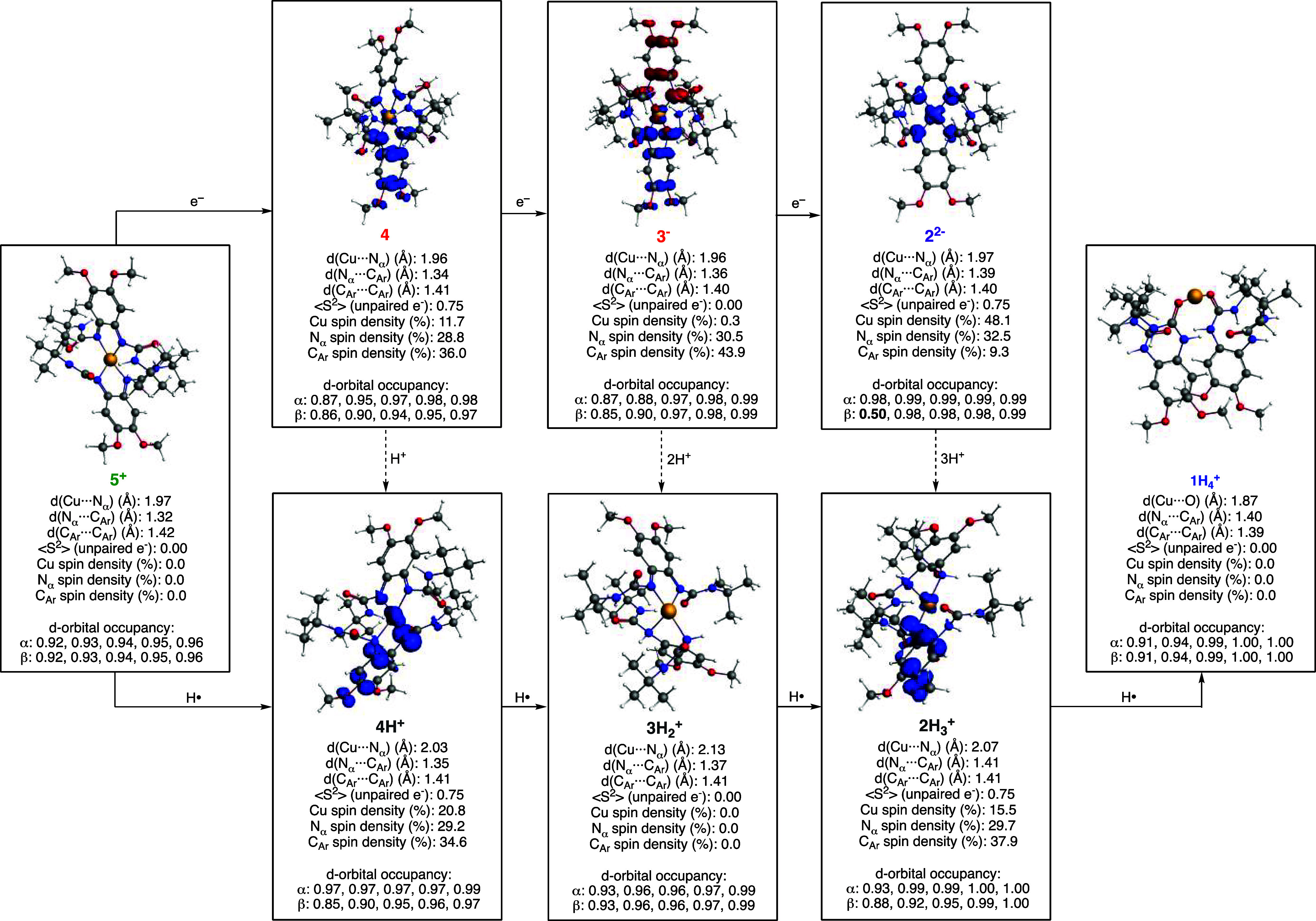
DFT computations on the electron structures of **5**^**+**^, **4**, **4H**^**+**^, **3**^**–**^, **3H**_**2**_^**+**^, **2**^**2–**^, **2H**_**3**_^**+**^, and **1H**_**4**_^**+**^.

In our previous ECPB report, we computed the electronic structure of complex **5**^**+**^ and the reduced species **4**, **3**^**–**^, and **2**^**2–**^ (Figure 9).^6^ For this paper, we calculated the structures of **5**^**+**^, **4**, **3**^**–**^, and **2**^**2–**^ again by fixing the position of the MeO substituents of the ligand in the plane of the aromatic ring and the CH_3_ molecule pointing away from each other (see details in the SI). For all of the complexes, the new structures were substantially lower in energy than those included in the previous report. However, minor changes were observed in the geometry and electronic structure of the complexes. The computed Cu···N_α_, N_α_···C_Ar_, and C_Ar_···C_Ar_ distances, spin-density plots, and d-orbital occupancies allowed us to determine the oxidation state of the metal ion (Cu^I^, Cu^II^, or Cu^III^) and the oxidation state of the ligand (catecholate-like, semiquinone-like, or quinone-like). Paradoxically, we found that the most reduced species (complex **2**^**2–**^) was formulated as a Cu^II^ ion bound by fully reduced catecholate-like ligands ([(L^2–^)_2_Cu^II^]^2–^, in agreement with the SC-XRD and electron paramagnetic resonance data), while the rest of the complexes (**3**^**–**^, **4**, and **5**^**+**^) were described as Cu^I^ ions bound by oxidized forms of the redox-active ligand (e.g., **5**^**+**^ as a Cu^I^ ion bound by two quinone-like ligands, [(L)_2_Cu^I^]^2–^). Similar redox-induced intramolecular electron-transfer processes have been recently described by Himmel and co-workers in the reduction of Cu complexes bearing redox-active urea azine and thiourea azine ligands.^18^

To have a deeper understanding of the ECPB reactivity of **5**^**+**^/**1H**_**4**_^**+**^, we also computed the structures of the Cu species involved in the 4H^+^/4e^–^ transformations, namely, **4H**^**+**^, **3H**_**2**_^**+**^, **2H**_**3**_^**+**^, and **1H**_**4**_^**+**^ (Figure 9). These computations included optimization of the complexes in different spin states (see details in the SI). Complex **4H**^+^ was described as a Cu^I^ ion (d-orbital occupancy suggested a d^10^ configuration) bound by a fully oxidized ligand (quinone-like) and a protonated semiquinone-like ligand in a doublet state (the spin-density plot suggested that the unpaired electron was mainly localized in the protonated ligand).

The computed electronic structure of complex **3H**_**2**_^**+**^ consisted of a diamagnetic cuprous complex in which one of the ligands is fully oxidized (quinone-like ligand) and the other ligand is in the full protonated/reduced form. Computations of the complex **3H**_**2**_^**+**^ in which both ligands are partially protonated and reduced (i.e., Cu ion bound by two monoprotonated semiquinone-like ligands) were also carried out, and we found that the resulting diamagnetic species (Cu^I^ ion bound by two protonated semiquinone-like ligands antiferromagnetically coupled) was 10 kcal/mol higher in energy than the other **3H**_**2**_^**+**^ isomer (see the SI).

Computations on **2H**_**3**_^**+**^ suggested the formation of a Cu complex coordinated only by three N atoms in a distorted T-shaped geometry. Our calculations formulate this species as a cuprous complex in a doublet state in which one of the ligands is fully protonated/reduced and the other ligand is in the protonated semiquinone-like form. Interestingly, the computed structure of **1H**_**4**_^**+**^ depicted a Cu ion bound only by two O atoms of the ureanyl substituents of the fully reduced form of the ligand (Figure 9). This linear complex was formulated as a diamagnetic cuprous species, in agreement with our NMR characterization. We also computed a geometric isomer of **1H**_**4**_^**+**^ in which the Cu^I^ ion was coordinated by three N_α_ atoms of the ligand scaffold (similar to **2H**_**3**_^**+**^), but the energy of this isomer was found to be substantially higher in energy (see the SI).

Analysis of the geometric and electronic changes upon the sequential reductive protonation of **5**^**+**^ to **1H**_**4**_^**+**^ revealed elongation of the Cu···N_α_ and N_α_···C_Ar_ bonds and a shortening of the C_Ar_···C_Ar_ distances, consistent with protonation/reduction of the redox-active ligand scaffold. Our computations revealed no changes in the oxidation state of the metal ion throughout the **5**^**+**^/**1H**_**4**_^**+**^ series. We believe that the fact that all of the species involved in the ECPB reactivity are Cu^I^ complexes might favor fast disproportionation and ligand-exchange reactions, which lead to fast regeneration of the **5**^**+**^/**1H**_**4**_^**+**^ equilibrium.

The thermochemistry of the PCET process involved in the **5**^**+**^/**1H**_**4**_^**+**^ ECPB was also analyzed by density functional theory (DFT) calculations (Table 3; see also the SI for details). The computed BDFE_avg._ value of the **5**^**+**^/**1H**_**4**_^**+**^ couple was found to be remarkably close to that found experimentally (70.0 vs 70.3 kcal/mol). Likewise, the computed BDFE of the **2H**_**3**_^**+**^/**1H**_**4**_^**+**^ couple also agreed with the experiment (83.8 vs 80 kcal/mol). Conversely, the computed BDFE of the **5**^**+**^/**4H**^**+**^ couple was found to be significantly lower than that obtained experimentally (58.0 vs 76 kcal/mol). This difference could be attributed to the divergence in the p*K*_a_ values of the **4**/**4H**^**+**^ couple obtained experimentally (∼16) and computationally (∼4.7) because the computed and experimental values of the reduction potential of the **5**^**+**^/**4** couple were similar (−0.67 vs −0.60 V vs Fc^0/+^). It is worth mentioning that the protonation of **4** was found to be irreversible; hence, the p*K*_a_ value obtained experimentally might be inaccurate. We also would like to note that the computed p*K*_a_ was obtained with the COSMO dielectric continuum solvation model and without the presence of implicit solvent molecules, which might have a substantial impact on the thermodynamics of proton transfer.^19^ Despite these uncertainties, the computed BDFEs would also fit in our thermodynamic analysis of the ECPB reactivity (Figure 8), in which the disproportionation reactions are thermodynamically favored when BDFE_1_ < BDFE_4_ (58.8 < 83.8) and the ligand-exchange reactions are favored when BDFE_1_ + BDFE_2_ < BDFE_3_ + BDFE_4_ (58.8 + 75.9 < 62.2 + 83.8).

**Table 3:** Computed BDFE, p*K*_a_, and *E*_1/2_ Values for the PCET Events Involved in Cu-Based ECPBs

		experimental	DFT
BDFE (kcal/mol)	**5**^**+**^/**4H**^**+**^	∼76	58.0
	**4H**^**+**^/**3H**_**2**_^**+**^	n.d.	75.9
	**3H**_**2**_^**+**^/**2H**_**2**_^**+**^	n.d.	62.2
	**2H**_**3**_^**+**^/**1H**_**4**_^**+**^	∼80	83.8
	**5**^**+**^/**1H**_**4**_^**+**^	70.3	70.0
	**^MeO-H^5**^**+**^/**^MeO-H^1H**_**4**_^**+**^	63.5	67.9
*E*_1/2_ (V vs Fc^0/+^)	**5**^**+**^/**4**	–0.60	–0.67
	**4**/**3**^**–**^	–0.69	–1.03
	**3**^**–**^/**2**^**2–**^	–0.89	–1.42
p*K*_a_	**4**/**4H**^**+**^	∼16	4.7

## Conclusions

In this paper, we studied the structure and reactivity of a series of ECPBs based on Cu and redox-active ligands based on *o*-phenylenediamine capable of performing reversible 4H^+^/4e^–^ transformations. The 4H^+^/4e^–^ thermochemistry (BDFE_avg._) of the ECPBs (namely, **5**^**+**^/**1H**_**4**_^**+**^, **^MeO-H^5**^**+**^/**^MeO-H^1H**_**4**_^**+**^, and **^Me-H^5**^**+**^/**^Me-H^1H**_**4**_^**+**^) was determined by OCP measurements. The Cu-based ECPBs were reacted with PCET reagents (O–H and N–H bonds), which resulted in multiproton/multielectron transformations controlled by the thermochemistry of the PCET reaction (e.g., difference between the BDFE_avg._ values of the ECPB and the PCET reagent). Experimental and computational analysis suggested significant differences in the thermochemistry of the stepwise and average PCET transformations involved in the ECPB reactivity. We theorized that the “redox unleveling” behavior exhibited by the Cu-ECPBs, as well as the fact that all of the species involved in the ECPB reactions are Cu^I^ complexes, might enable fast disproportionation and ligand-exchange reactions to restore the **^X-R^5**^**+**^/**^X-R^1H**_**4**_^**+**^ equilibria. Modification of the stereoelectronic properties of the Cu-based ECPB systems led to marked variations in *E*_1/2_ compared to p*K*_a_. This “thermochemical decompensation” will be further exploited in the design of new metal-based ECPB systems with broader BDFE ranges.

## Supporting Information Available

Experimental details, including characterization data, spectra, crystallographic data, electrochemical measurements, and computational studies (PDF)
