# Supplementary material for: Tuning the Thermochemistry and Reactivity of a Series of Cu-Based 4H+/4e– Electron-Coupled-Proton Buffers
Source: Inorg Chem. 2024 May 9;63(20):9014–25. doi: 10.1021/acs.inorgchem.4c00835 (PMC11110016; doi:10.1021/acs.inorgchem.4c00835)
Supplement: Supplementary file 1 — ic4c00835_si_001.pdf [file ic4c00835_si_001.pdf]

Supporting Information

for

Tuning the Thermochemistry and Reactivity of a Series of Cu-based $4H^+/4e^-$ Electron-Coupled-Proton Buffers.

Tong Wu,† Ankita Puri,† Yi Lin Qiu,† Daniel Ye,† Rajdeep Sarma,† Yiwen Wang,† Tomasz Kowalewski,† Maxime A. Siegler,¶ Marcel Swart*,Ψ and Isaac Garcia-Bosch*,†

†Department of Chemistry, Carnegie Mellon University, Pittsburgh, Pennsylvania 15213, United States.

¶Johns Hopkins University, Baltimore, Maryland 21218, United States

ΨUniversity of Girona, Campus Montilivi (Ciències), IQCC, Girona, Spain; ICREA, Pg. Lluís Companys 23, 08010, Barcelona, Spain.

Table of Contents

1. Physical methods and materials……………………………..……………..……..S3

2. Synthesis and characterization of complexes……………………..……………..S4

3. Electrochemistry…………………..……………………….……………..…………S11

4. OCP measurements of PCET substrates and Cu ECPBs ………………………..S14

5. Reactivity and of ECPBs toward PCET substrates…………………………..….S20

6. Decoupled 4H$^+$/4e$^-$ reduction/dehydrogenation reactivity……………………....S28

7. BDFE analysis ……………………………………………………………….……..S30

8. Ligand exchange reactions……………………………...…………………S34

9. DFT calculations.……………..………………………………………………………S40

10. References……………………………………………………………..…………S68

1. Physical methods and materials.

Preparation of some UV-vis or NMR samples were carried out under anaerobic conditions in an mBRAUN MB-Unilab Pro SP Glovebox system. UV-Vis measurements were carried out by using a Hewlett Packard 8454 diode array spectrophotometer with a 10 mm path quartz cell. The spectrometer was equipped with HP Chemstation software and a Unisoku cryostat for low temperature experiments. NMR spectra were recorded in 7-inch, 5-mm o.d. NMR tubes on a 500 MHz NMR (Bruker AvanceTM 500) to acquire spectra with 32 cumulative scans. Elemental analysis was performed by the ALS Group USA, Corp. dba ALS Environmental (Tucson, Arizona). Electrochemical measurements were carried out on a model 620E Electrochemical Workstation (CH Instruments).

Reagents: All reagents and solvents were purchased at the highest level of purity and used as received except as noted. Solvents were purified and dried by passing through an activated alumina purification system (INERT Pure Solv) or by conventional distillation techniques. DMF (dimethylformamide) was distilled before use.

1,8-dichloro-9,10-dihydroxyanthracene (1,8-Cl_2-H_2AQ), 5,10-dihydrophenazine (DHP), 1,4-dihydroxynaphthalene (1,4-H_2NQ), 2,2,6,6-tetramethyl-1-hydroxypiperidine (TEMPOH), 2,6-dimethoxy-1,4-hydroquinone (2,6-MeO_2-H_2Q), 2,6-dimethyl-1,4-hydroquinone (2,6-Me_2-H_2Q), 2,6-dichloro-1,4-hydroquinone (2,6-Cl_2-H_2Q) were prepared according to literature procedures[1].

2,4,6-tri-tert-butylphenoxyl radical (2,4,6-TTBP•)[2], 4-methoxy-2,6-di-tertbutylphenoxyl radical (4-MeO-2,6-DTBP•)[3] were prepared following a modified literature procedure: Under N_2 flow, the corresponding phenols (5 mmol) were dissolved in 15 mL toluene or diethyl ether, then 15 mL degassed 1M NaOH solution and 15 mmol $K_3Fe(CN)_6$ (as solid) were added while stirring. After 2 hours, the water layer (bottom) was carefully taken away by a syringe, and the remaining organic solution was removed under vacuum. In the glovebox, the crude was dissolved in 15 mL Et_2O and filtered through a funnel. The Et_2O solution was dried to acquire the phenoxyl radical. 2,4,6-TTBP• was obtained as deep blue shiny solid and crystallized in MeCN at -35 ^oC for further use; 4-MeO-2,6-DTBP• was obtained as purple oil and kept at -35 ^oC for further use.

2. Synthesis and characterization of complexes.

Synthesis and characterization of $^{MeO\text{-}H}$5^{+}:

In the glovebox, 168.0 mg of the $^{MeO\text{-}H}$**LH$_2$** ligand (1 mmol) were dissolved in 5 mL DMF in a 20 mL vial equipped with a stir bar. 186.2 mg of **[Cu^{I}(CH$_3$CN)$_4$]PF$_6$** (0.5 mmol) were added to the ligand solution. Then the mixture was capped with a septum, transferred out the glove box and stirred under O_2 flow for 2 hours. Later the solution was filtered and washed with Et_2O to obtain dark purple powder (200mg, 70 %). Crystals suitable for single crystal X-ray diffraction were acquired by slow oxidation of a THF solution of $^{MeO\text{-}H}$**LH$_2$** ligand and **[Cu^{I}(CH$_3$CN)$_4$]PF$_6$** in aerobic condition.

^{1}H-NMR (DMF-d$_7$): 10.70 (s, 4H, N-**H**), 6.51 (s, 4H, Ph-**H**), 3.90 (s, 12H, OCH$_2$-**H**).

Elemental Analysis: ($C_{16}H_{20}CuF_6N_4O_4P$·THF 1.5 H_2O) calc. C: 37.53%, H: 4.88%; N: 8.75%; exp. C: 37.54%, H: 4.38%; N: 8.29%

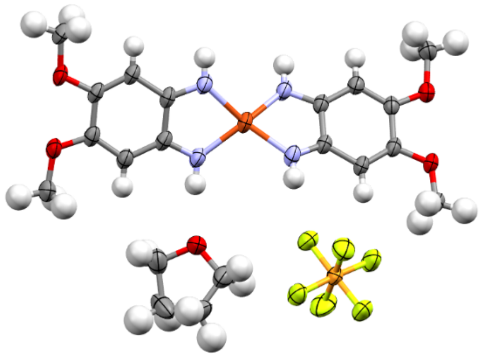

Figure S1. Displacement ellipsoid plot (50% probability level) of $^{MeO\text{-}H}$**5^{+}** at 110(2) (K). Disorder is omitted for the sake of clarity.

All reflection intensities were measured at 110(2) K using a SuperNova diffractometer (equipped with Atlas detector) with Cu *K*α radiation (λ = 1.54178 Å) under the program CrysAlisPro (Version CrysAlisPro 1.171.42.49, Rigaku OD, 2022). The same program was used to refine the cell dimensions and for data reduction. The structure was solved with the program SHELXS-2018/3 (Sheldrick, 2018) and was refined on F^2 with SHELXL-2018/3 (Sheldrick, 2018). Analytical numeric absorption correction using a multifaceted crystal model was done using CrysAlisPro. The temperature of the data collection was controlled using the system Cryojet (manufactured by Oxford Instruments). The H atoms were placed at calculated positions (unless otherwise specified) using the instructions AFIX 23, AFIX 43 or AFIX 137 with isotropic displacement parameters having values 1.2 or 1.5 U_{eq} of the attached C atoms. The H atoms attached to N1X and N2X (X = A, B) were found from difference Fourier maps, and their coordinates were refined pseudofreely using the DFIX instruction in order to keep the N–H distances within an acceptable range. The structure is partly disordered. The PF_6^- counterion is found to be disordered over two orientations, and the occupancy factor of the major component of the disorder refines to 0.68(3).The absolute configuration has been established by anomalous-dispersion effects in diffraction measurements on the crystal, and the Flack and Hooft parameters refine to -0.007(17) and -0.005(15), respectively.

Table S1. Crystallographic data for $^{MeO-H}$**5^{+}**.

Complex $^{MeO-H}$**5^{+}**	
Chemical formula	$2(C_{16}H_{20}CuN_4O_4)\cdot 2(F_6P)\cdot C_4H_8O$
M_r	1153.84
Crystal system, space group	Orthorhombic, *Fdd*2
Temperature (K)	110
a, *b*, *c* (Å)	29.5892 (6), 25.6682 (6), 11.9791 (3)
V (Å^3)	9098.1 (4)
Z	8
Radiation type	Cu $K\alpha$
μ (mm^{-1})	2.82
Crystal size (mm)	0.47 × 0.30 × 0.05
Data collection	
Diffractometer	SuperNova, Dual, Cu at zero, Atlas
Absorption correction	Analytical<br>*CrysAlis PRO* 1.171.42.49 (Rigaku Oxford Diffraction, 2022) Analytical numeric absorption correction using a multifaceted crystal model based on expressions derived by R.C. Clark & J.S. Reid. (Clark, R. C. & Reid, J. S. (1995). Acta Cryst. A51, 887-897) Empirical absorption correction using spherical harmonics, implemented in SCALE3 ABSPACK scaling algorithm.
T_{min}, T_{max}	0.436, 0.884
No. of measured, independent and observed [$I > 2\sigma(I)$] reflections	28184, 4458, 4264
R_{int}	0.037
$(\sin \theta/\lambda)_{max}$ (Å^{-1})	0.616
Refinement	
$R[F^2 > 2\sigma(F^2)]$, $wR(F^2)$, S	0.031, 0.085, 1.05
No. of reflections	4458
No. of parameters	386
No. of restraints	236
H-atom treatment	H atoms treated by a mixture of independent and constrained refinement
$\Delta\rho_{max}$, $\Delta\rho_{min}$ (e Å^{-3})	0.27, -0.31
Absolute structure	Flack x determined using 1941 quotients [(I+)-(I-)]/[(I+)+(I-)] (Parsons, Flack and Wagner, Acta Cryst. B69 (2013) 249-259).
Absolute structure parameter	-0.007 (17)

Synthesis and characterization of $^{Me\text{-}H}$5⁺:

In the glovebox, 136.0 mg of the $^{\mathbf{Me\text{-}H}}$**LH$_2$** ligand (1 mmol) were dissolved in 5 mL DMF in a 20 mL vial equipped with a stir bar. 186.2 mg of **[Cu^{I}(CH$_3$CN)$_4$]PF$_6$** (0.5 mmol) were added to the ligand solution. Then the mixture was capped with a septum, transferred out the glove box and stirred under O_2 flow for 2 hours. Later the solution was filtered to obtain dark purple powder (165mg, 60 %). Crystals suitable for single crystal X-ray diffraction were acquired by slow oxidation of a THF solution of $^{\mathbf{Me\text{-}H}}$**LH$_2$** ligand and **[Cu^{I}(CH$_3$CN)$_4$]PF$_6$** in aerobic condition.

^{1}H-NMR (DMF-d$_7$): 11.16 (s, 4H, N-**H**), 6.95 (s, 4H, Ph-**H**), 2.06 (s, 12H, CH$_2$-**H**).

Elemental Analysis: ($C_{16}H_{20}CuF_6N_4P$·THF 0.5H_2O) calc. C: 43.05%, H: 5.24%; N: 10.05%; exp. C: 43.22%, H: 4.82%; N: 10.20%

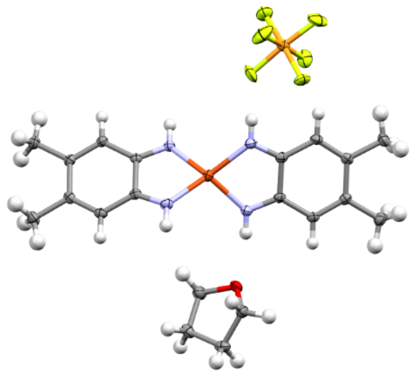

Figure S2. Displacement ellipsoid plot (50% probability level) of $^{\mathbf{Me\text{-}H}}$**5⁺** at 110(2) (K). Disorder is omitted for the sake of clarity.

All reflection intensities were measured at 110(2) K using a SuperNova diffractometer (equipped with Atlas detector) with Mo *K*α radiation (λ = 0.71073 Å) under the program CrysAlisPro (Version CrysAlisPro 1.171.42.49, Rigaku OD, 2022). The same program was used to refine the cell dimensions and for data reduction. The structure was solved with the program SHELXS-2018/3 (Sheldrick, 2018) and was refined on F^2 with SHELXL-2018/3 (Sheldrick, 2018). Numerical absorption correction based on gaussian integration over a multifaceted crystal model was applied using CrysAlisPro. The temperature of the data collection was controlled using the system Cryojet (manufactured by Oxford Instruments). The H atoms were placed at calculated positions using the instructions AFIX 23, AFIX 43 or AFIX 137 with isotropic displacement parameters having values 1.2 or 1.5 U_{eq} of the attached C atoms. The H atoms attached to N1X and N2X (X = A, B) were found from difference Fourier maps, and their coordinates were refined pseudofreely using the DFIX instruction in order to keep the N–H bond distances within an acceptable range. The structure is partly disordered. The PF_6^- counterion is found to be disordered over three orientations, and their occupancy factors refine to 0.440(3), 0.347(3) and 0.213(3).

Table S2. Crystallographic data for $^{\text{Me-H}}$**5**$^{+}$.

Complex $^{\text{Me-H}}$**5**$^{+}$	
Chemical formula	$C_{16}H_{20}CuN_4{\cdot}F_6P{\cdot}C_4H_8O$
M_r	548.97
Crystal system, space group	Monoclinic, $P2_1/c$
Temperature (K)	110
a, *b*, *c* (Å)	14.1692 (6), 12.4302 (4), 14.3249 (6)
β (°)	115.776 (5)
V (Å^3)	2271.95 (18)
Z	4
Radiation type	Mo $K\alpha$
μ (mm^{-1})	1.10
Crystal size (mm)	0.33 × 0.15 × 0.12
Data collection	
Diffractometer	SuperNova, Dual, Cu at zero, Atlas
Absorption correction	Gaussian<br>*CrysAlis PRO* 1.171.42.49 (Rigaku Oxford Diffraction, 2022) Numerical absorption correction based on gaussian integration over a multifaceted crystal model Empirical absorption correction using spherical harmonics, implemented in SCALE3 ABSPACK scaling algorithm.
T_{min}, T_{max}	0.533, 1.000
No. of measured, independent and observed [*I* > 2□(*I*)] reflections	32492, 5226, 4591
R_{int}	0.034
$(\sin \theta/\lambda)_{max}$ (Å^{-1})	0.650
Refinement	
$R[F^2 > 2\sigma(F^2)]$, $wR(F^2)$, S	0.028, 0.070, 1.06
No. of reflections	5226
No. of parameters	425
No. of restraints	596
H-atom treatment	H atoms treated by a mixture of independent and constrained refinement
$\Delta\rho_{max}$, $\Delta\rho_{min}$ (e Å^{-3})	0.48, -0.27

Preparation of $^{MeO\text{-}H}1H_4^+$ and $^{Me\text{-}H}1H_4^+$

NMR characterization: In the glovebox, $^{MeO\text{-}H}\mathbf{1H_4^+}$ or $^{Me\text{-}H}\mathbf{1H_4^+}$ were prepared by mixing 2 equiv of $^{MeO\text{-}H}\mathbf{LH_2}$ or $^{Me\text{-}H}\mathbf{LH_2}$ and 1 equiv of **$[Cu(CH_3CN)_4]PF_6$** in DMF (for UV-vis) or DMF-d_7 (for NMR). The ^{1}H-NMR spectra are shown below. As described in the manuscript, the presence of $^{MeO\text{-}H}\mathbf{5^+}$ or $^{Me\text{-}H}\mathbf{5^+}$ precluded the slow disproportionation of $^{MeO\text{-}H}\mathbf{1H_4^+}$ or $^{Me\text{-}H}\mathbf{1H_4^+}$ (see Scheme S1 below).

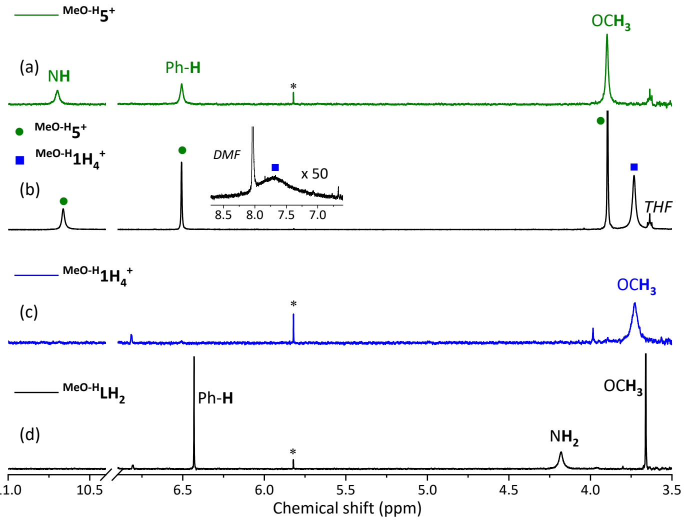

Figure S3. NMR spectra of (a) $^{MeO\text{-}H}\mathbf{5^+}$, (b) mixture of $^{MeO\text{-}H}\mathbf{5^+}$ and $^{MeO\text{-}H}\mathbf{1H_4^+}$ at 1:1 ratio, (c) $^{MeO\text{-}H}\mathbf{1H_4^+}$ and (d) $^{MeO\text{-}H}\mathbf{LH_2}$ in DMF-d_7. Note: the noted peak is impurity from solvent; only broad peak of CH_3 was observed for $^{MeO\text{-}H}\mathbf{1H_4^+}$.

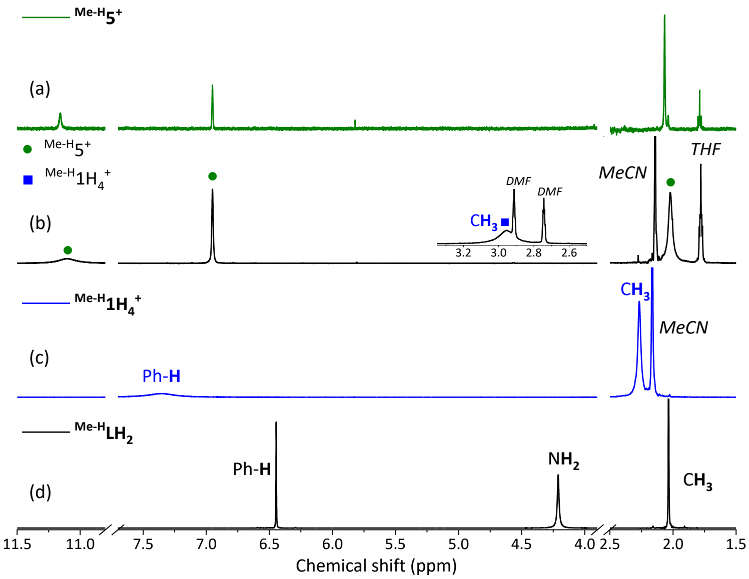

Figure S4. NMR spectra of (a) $^{Me\text{-}H}\mathbf{5^+}$, (b) mixture of $^{Me\text{-}H}\mathbf{5^+}$ and $^{Me\text{-}H}\mathbf{1H_4^+}$ at 1:1 ratio, (c) $^{Me\text{-}H}\mathbf{1H_4^+}$ and (d) $^{Me\text{-}H}\mathbf{LH_2}$ in DMF-d_7.

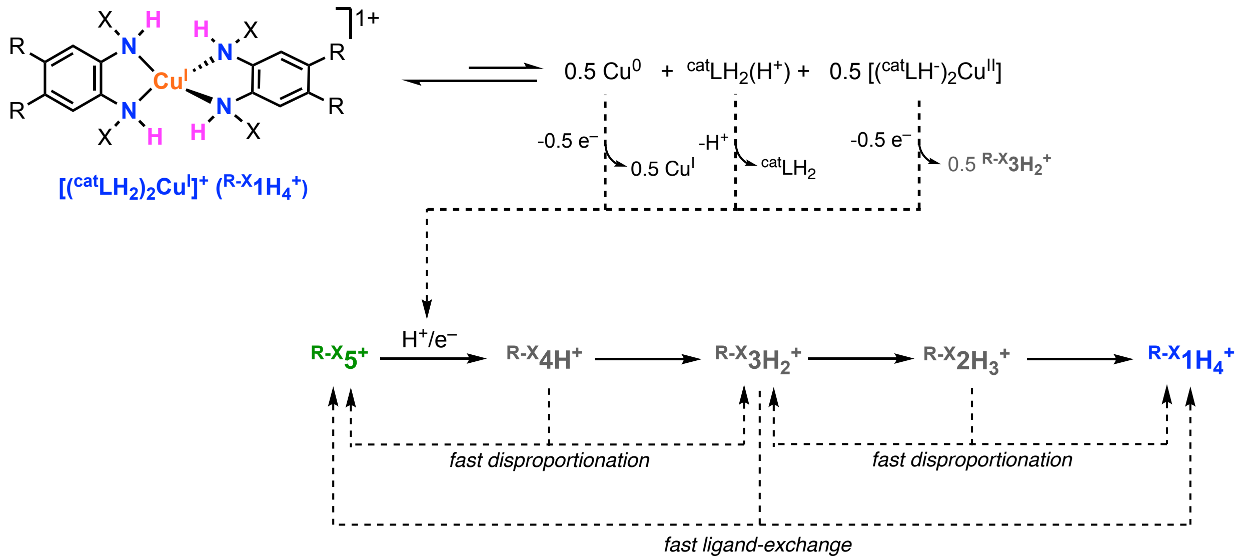

Scheme S1. Proposed mechanism for the disproportionation of **$^{R\text{-}X}$1H$_4^+$** precluded by the presence of **$^{R\text{-}X}$5$^+$.**

UV-vis characterization: In the glovebox, 3 mL of a $^{\text{MeO-H}}$**5**$^+$or $^{\text{Me-H}}$**5**$^+$ solution (0.0625 mM) in DMF were placed in a 10 mm path quartz cell capped with a rubber septum. UV-vis characterization was conducted at room temperature under Ar flow. 0.0625 mM $^{\text{MeO-H}}$**1H**$_4^+$ or $^{\text{Me-H}}$**1H**$_4^+$ solution in DMF were generated in the glovebox by addition of 1 equiv of freshly prepared **[Cu(MeCN)$_4$]PF$_6$** solution into 2 equiv of $^{\text{MeO-H}}$**LH**$_2$ or $^{\text{Me-H}}$**LH**$_2$ solution.

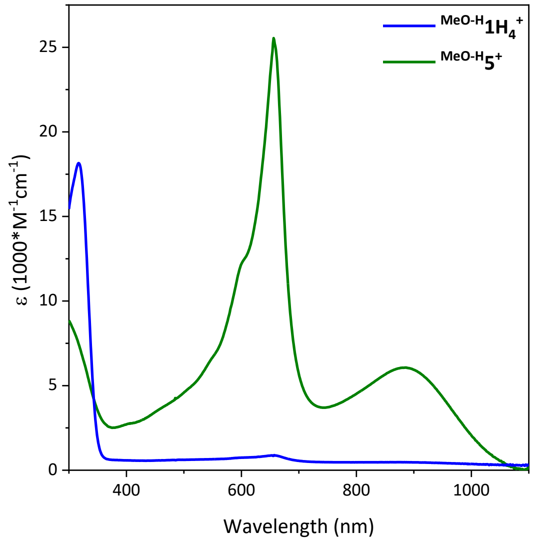

Figure S5. UV-vis of $^{\text{MeO-H}}$**1H**$_4^+$ (blue) and $^{\text{MeO-H}}$**5**$^+$ (green) in DMF at room temperature. Note: MeO-H1H4+ in DMF slowly disproportionates to form $^{\text{MeO-H}}$**5**$^+$ (see peak at 660 nm in black spectrum).

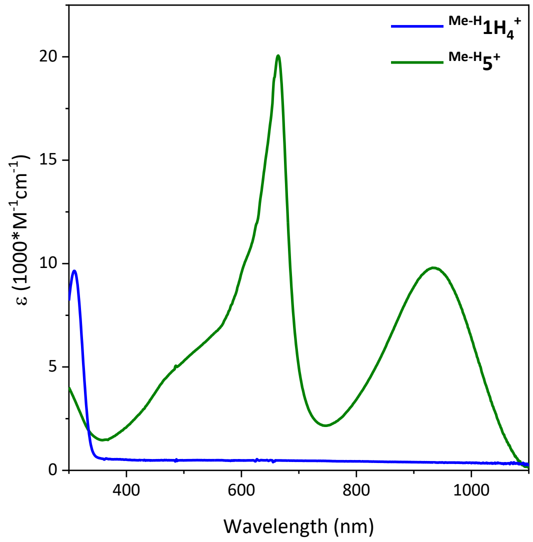

Figure S6. UV-vis of $^{\text{Me-H}}$**1H**$_4^+$ (blue) and $^{\text{Me-H}}$**5**$^+$ (green) in DMF at room temperature.

3. Electrochemistry

Cyclic voltammetry: 3 mL of a DMF solution of **$^{MeO\text{-}H}$5$^+$** or **$^{Me\text{-}H}$5$^+$** (1 mM) containing 0.1 M of **$[NBu_4]PF_6$** were prepared in the glovebox and were transferred to an electrochemical cell outside the glovebox, which was purged with Ar for 5 minutes (note: a conventional three-electrode cell was used with a glassy carbon working electrode, an Ag/$AgNO_3$ (0.01 M) and platinum wire as the counter electrode). The potentials were measured with respect to the Ag/$AgNO_3$ reference electrode and converted to $Fc^{+/0}$ ($Fc^{+/0}$ potential measured under the same experimental conditions). Cyclic voltammograms were obtained at scan rates of 100 mV/s. All electrochemical measurements were carried out under Ar atmosphere.

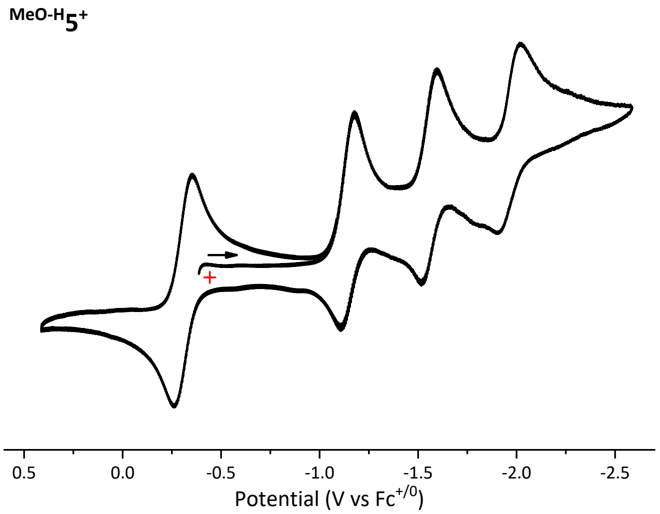

Figure S7. Cyclic voltammogram of **$^{MeO\text{-}H}$5$^+$** in DMF; scan rate = 100 mV /s; +open circuit potential.

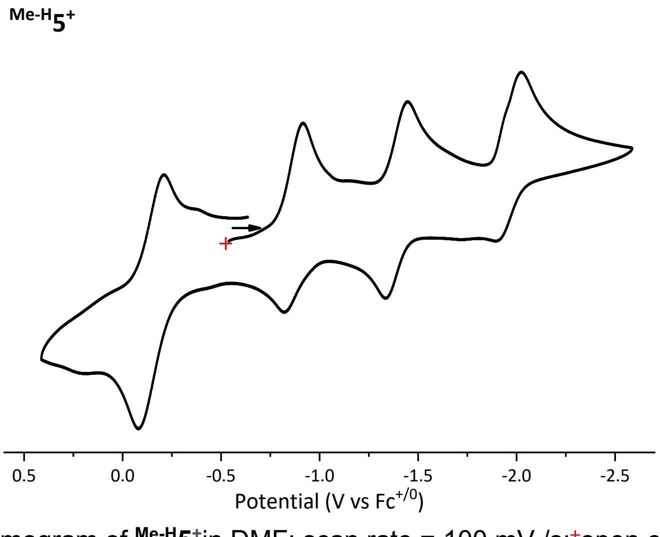

Figure S8. Cyclic voltammogram of **$^{Me\text{-}H}$5$^+$** in DMF; scan rate = 100 mV /s; +open circuit potential.

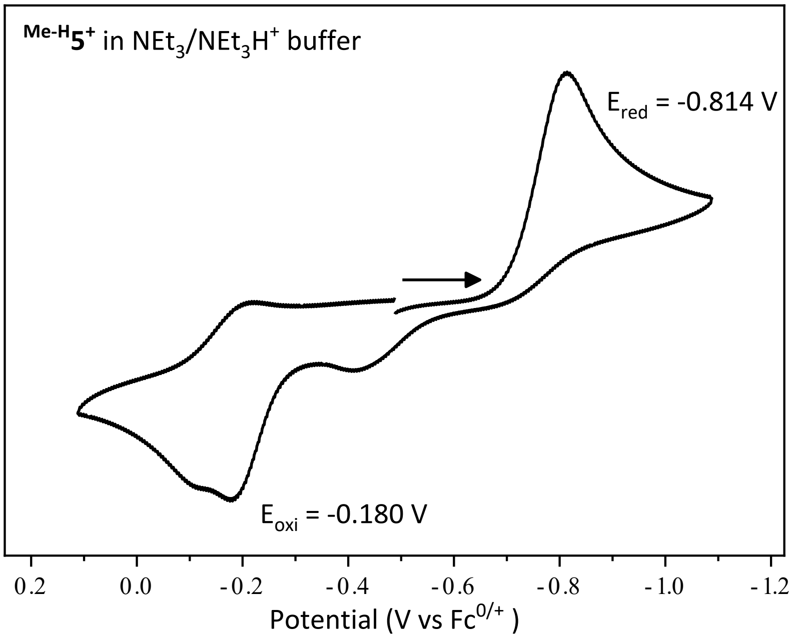

Figure S9. Cyclic voltammogram of $^{Me\text{-}H}\mathbf{5}^{+}$ in DMF in the presence of buffer; scan rate = 100 mV /s.

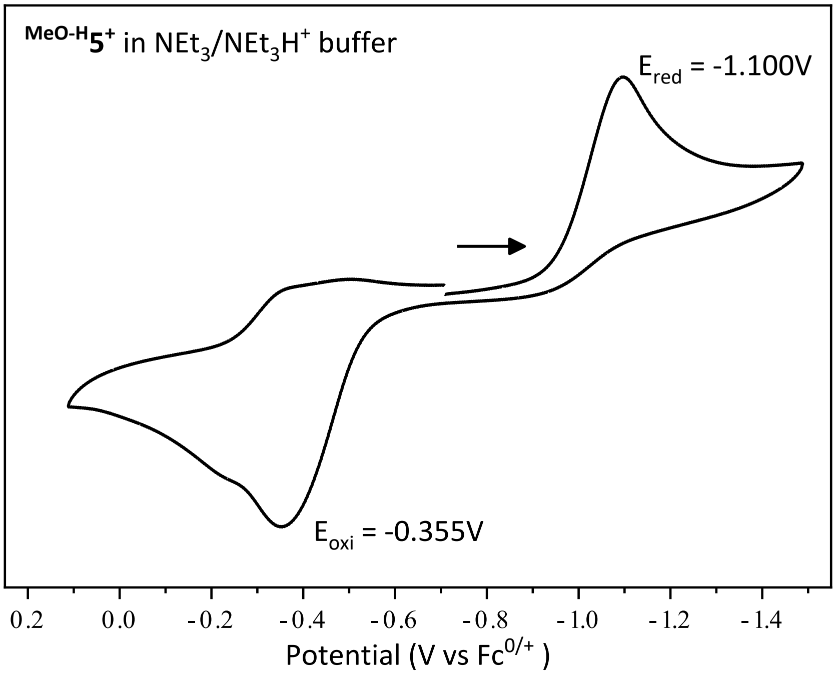

Figure S10. Cyclic voltammogram of $^{MeO\text{-}H}\mathbf{5}^{+}$ in DMF in the presence of buffer; scan rate = 100 mV /s.

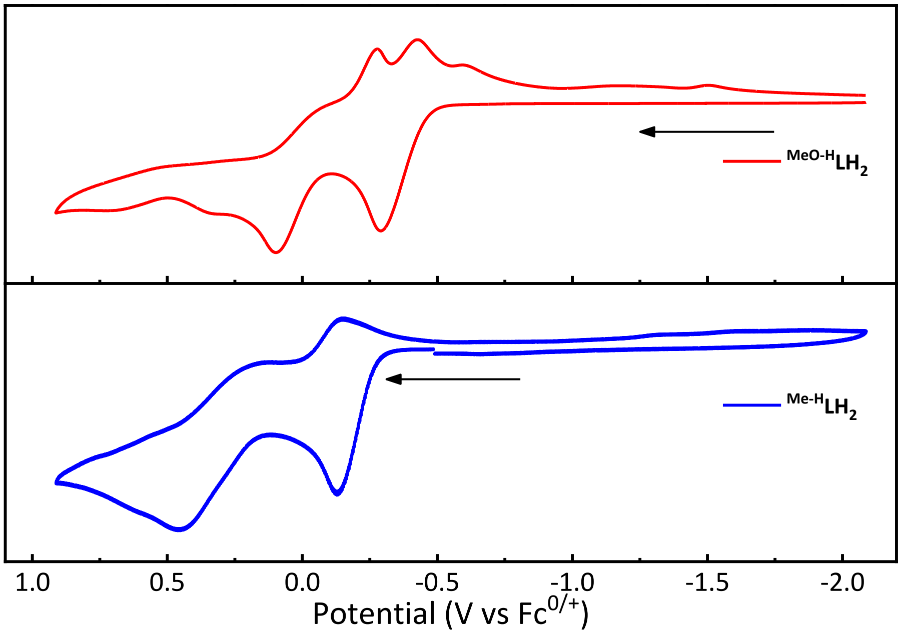

Figure S11. Cyclic voltammogram of $^{MeO-H}$**LH**$_2$ and $^{Me-H}$**LH**$_2$ in DMF; scan rate = 100 mV /s.

4. OCP measurements of PCET substrates and Cu ECPBs.

General procedure: In the glovebox, 3 mL of DMF solution containing 100 mM $[Bu_4N]PF_6$, 50 mM NEt_3H^+/NEt_3 (generated using 100 mM NEt_3 and 50 mM DMF-H^+ OTf^-) or pyridinium/pyridine buffer, 1 mM PCET reductant (e.g., 1,4-H_2Q) and 0.5 mM PCET oxidant (e.g., 1,4-BQ) was prepared. The open-circuit potential was allowed to equilibrate for around 5 minutes or until the potential was changing at a rate less than 0.005 mV/s. Under Ar flow, a desired amount of stock solution (20 mM) was titrated into the sample, and open-circuit potential was again measured. The process was repeated until the ratio of PCET substates changed from 2:1 to 1:2. After each OCP measurement, Fc (in DMF) was added as reference before a cyclic voltammetry (CV) measurement was carried out.

Calculation of standard potential and BDFEs:

$$E^o(XH_n/X, \text{V vs } H_2) = E^{o'}_{ocp}(XH_n/X, \text{V vs } Fc^{+/0}) + 0.059[pK_a(HA)] - E^o(H^+/H_2, \text{V vs } Fc^{0/+})$$

In the equation above, $E^{o'}_{ocp}(XH_n/X, \text{V vs } Fc^{+/0})$ is determined by OCP measurement and CV of Fc reference; pK_a(HA) is the pK_a of the selected acid/base buffer in DMF; $E^o(H^+/H_2, \text{V vs } Fc^{0/+})$ is the standard hydrogen potential vs $Fc^{0/+}$ in DMF (-0.662 V[4]).

$$BDFE_{avg}(XH_n/X) = 23.06E^o(XH_n/X, \text{V vs } H_2) + \Delta G^o(\tfrac{1}{2}H_2(g)/H^\bullet_{1M})$$

$\Delta G^o(\tfrac{1}{2}H_2(g)/H^\bullet_{1M})$ is the free energy (52.3 kcal/mol[4]) to convert $\tfrac{1}{2}H_2$ (g) to $H^\bullet_{1M}$ in DMF.

Error analysis: The standard potentials and BDFE values calculated from equations above are influenced by the pK_a values of the buffer in DMF. Change in the buffer identity leads to variance in the obtained BDFEs. For example, BDFE of 2,6-dichloro-1,4-hydroquinone should be slightly higher than that of 1,4-hydroquinone, but OCP measurement of 2,6-dichloro-1,4-hydroquinone in pyridinium/pyridine buffer (not compatible in in NEt_3H^+/NEt_3 buffer) gave a BDFE at 68.5 kcal/mol, which is 0.1 kcal/mol lower than BDFE of 1,4-hydroquinone measured in NEt_3H^+/NEt_3 buffer. Here we measured OCP of 1,4-hydroquinone in both buffers, and a 0.4 kcal/mol difference in BDFE was noticed (see below).

1,4-H_2Q/1,4-BQ

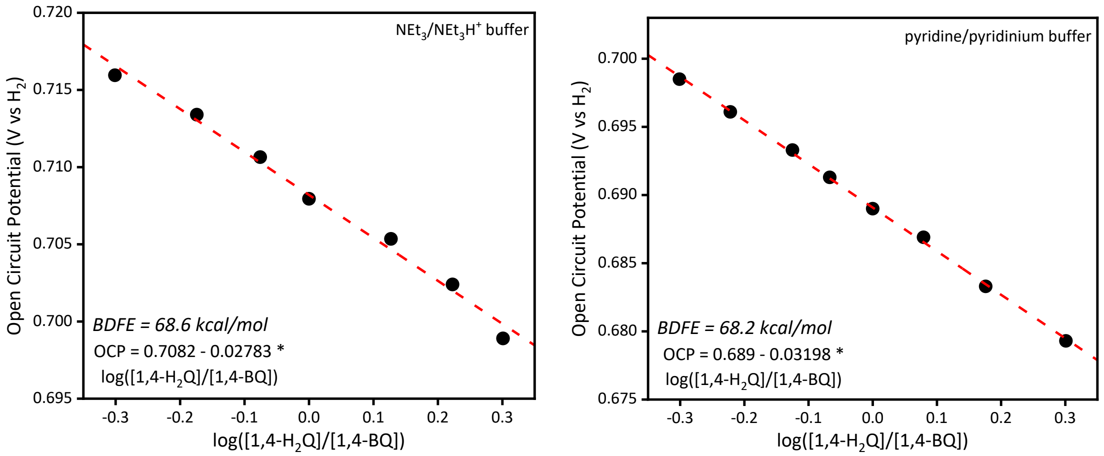

Figure S12. Open-circuit potential at different ratios of 1,4-H_2Q/1,4-BQ, plotted against the log ratios of the substrates. Data collected in (left) NEt_3/NEt_3H^+ buffer and (right) pyridine/pyridinium buffer.

To accurately study the reactivity of Cu-based ECPBs with all the PCET substrates, in this paper we use the standard potentials and BDFEs in <u>NEt_3H^+/NEt_3 buffer</u>.

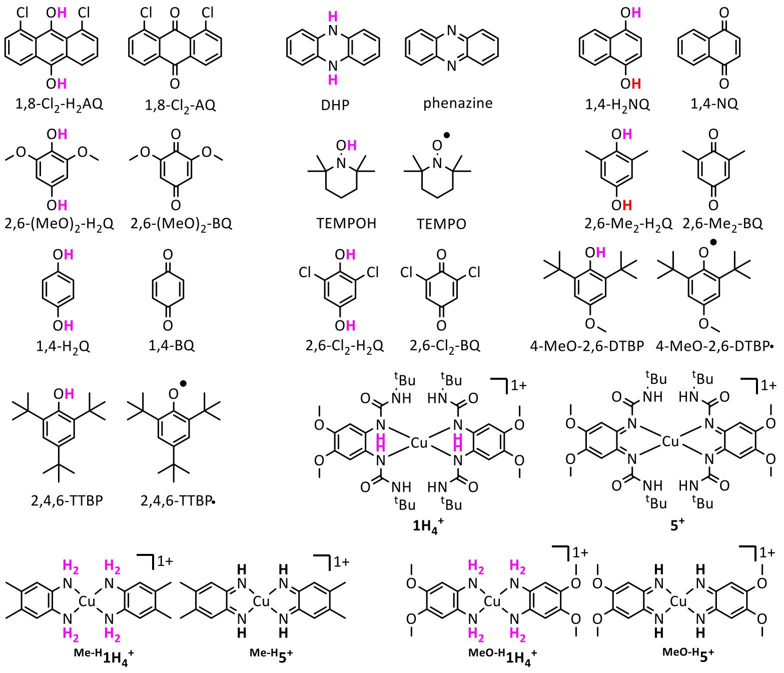

Figure S13. Structures of all substrates used in this paper, with names or abbreviations listed below. Relevant H atom(s) shown in red.

Table S3. Standard potentials and BDFEs of Cu-based ECPBs and PCET couples in DMF.

PCET couples	E^o (V vs H_2)	BDFE (kcal/mol)
1,8-Cl_2-H_2AQ/1,8-Cl_2-AQ	0.169	56.2
DHP/phenazine	0.269	58.5
$^{MeO-H}$**1**H_4^+/ $^{MeO-H}$**5**$^+$	0.486	63.5
+1,4-H_2NQ/1,4-NQ	0.500	63.8
2,6-(MeO)$_2$-H_2Q/2,6-(MeO)$_2$-BQ	0.514	64.1
TEMPOH/TEMPO[a]	0.582	65.7
2,6-Me_2-H_2Q/2,6-Me_2-BQ	0.604	66.2
$^{Me-H}$**1**H_4^+/ $^{Me-H}$**5**$^+$	0.626	66.7
1,4-H_2Q/1,4-BQ[b]	0.708	68.6
2,6-Cl_2-H_2Q/2,6-Cl_2-BQ	0.724	68.9
**1**H_4^+/**5**$^+$[a]	0.780	70.3
4-MeO-2,6-DTBP/4-MeO-2,6-DTBP·	0.851	71.9
2,4,6-TTBP/2,4,6-TTBP·	0.987	75.1

[a]Measured in ref[5]; [b]Measured in pyridinium/pyridine buffer, the values used were converted to those in NEt_3H^+/NEt_3 buffer.

1,8-Cl_2-H_2AQ/1,8-Cl_2-AQ

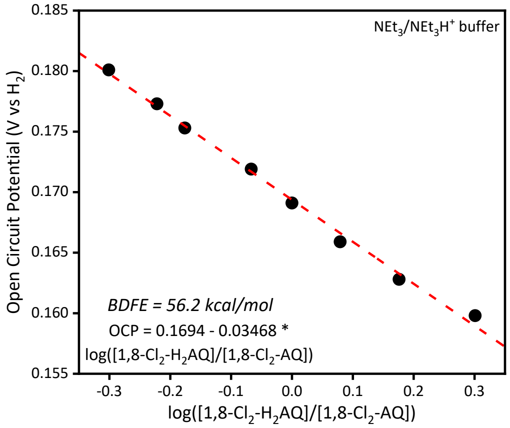

Figure S14. Open-circuit potential at different ratios of 1,8-Cl_2-H_2AQ/1,8-Cl_2-AQ, plotted against the log ratios of the substrates. Data collected in NEt_3/NEt_3H^+ buffer.

DHP/phenazine

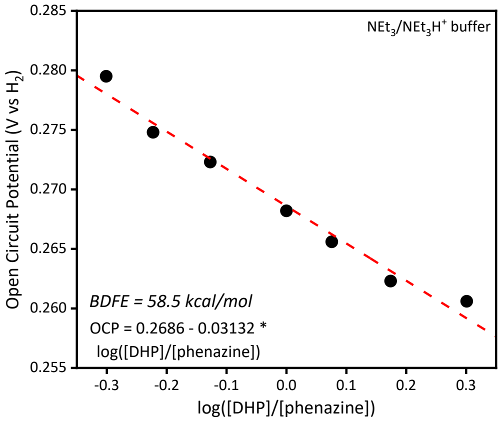

Figure S15. Open-circuit potential at different ratios of DHP/phenazine, plotted against the log ratios of the substrates. Data collected in NEt_3/NEt_3H^+ buffer.

$^{MeO\text{-}H}$1H_4^+/$^{MeO\text{-}H}$5$^+$

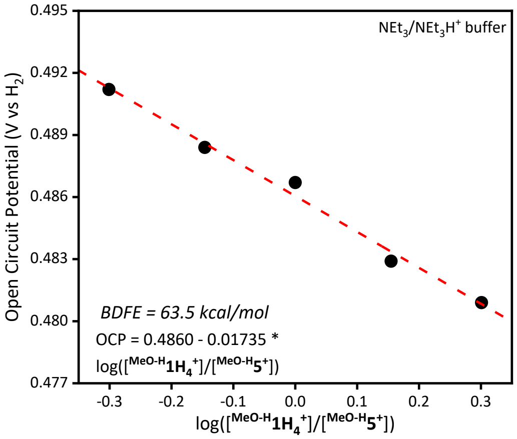

Figure S16. Open-circuit potential at different ratios of **$^{MeO\text{-}H}$1H_4^+/$^{MeO\text{-}H}$5$^+$**, plotted against the log ratios of the substrates. Data collected in NEt_3/NEt_3H^+ buffer.

1,4-H_2NQ/1,4-NQ

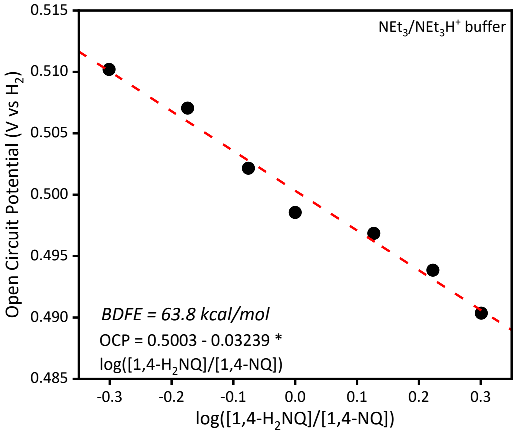

Figure S17. Open-circuit potential at different ratios of 1,4-H_2NQ/1,4-NQ, plotted against the log ratios of the substrates. Data collected in NEt_3/NEt_3H^+ buffer.

2,6-$(MeO)_2$-H_2Q/2,6-$(MeO)_2$-BQ

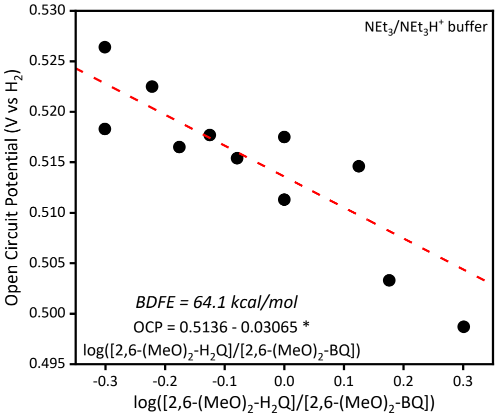

Figure S18. Open-circuit potential at different ratios of 2,6-$(MeO)_2$-H_2Q/2,6-$(MeO)_2$-BQ, plotted against the log ratios of the substrates. Data collected in NEt_3/NEt_3H^+ buffer.

2,6-Me_2-H_2Q/2,6-Me_2-BQ

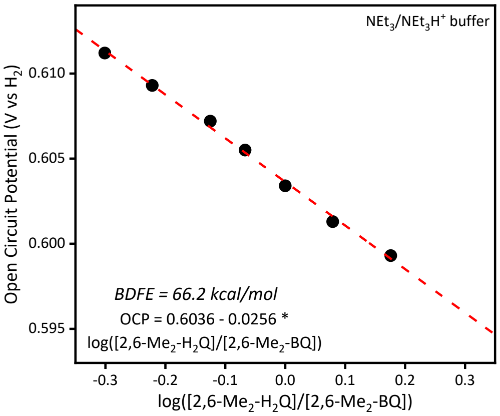

Figure S19. Open-circuit potential at different ratios of 2,6-Me_2-H_2Q/2,6-Me_2-BQ, plotted against the log ratios of the substrates. Data collected in NEt_3/NEt_3H^+ buffer.

$^{Me\text{-}H}$1H$_4^+$/$^{Me\text{-}H}$5$^+$

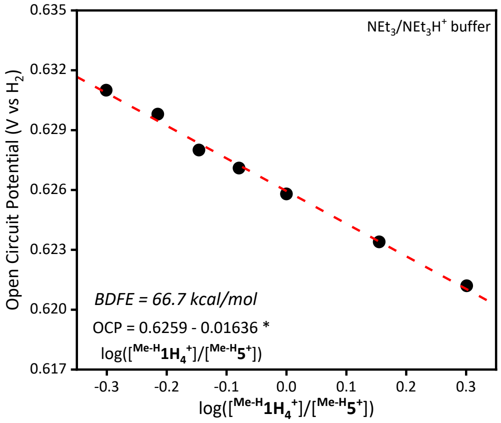

Figure S20. Open-circuit potential at different ratios of **$^{Me\text{-}H}$1H$_4^+$/$^{Me\text{-}H}$5$^+$**, plotted against the log ratios of the substrates. Data collected in NEt_3/NEt_3H^+ buffer.

2,6-Cl$_2$-H$_2$Q/2,6-Cl$_2$-BQ

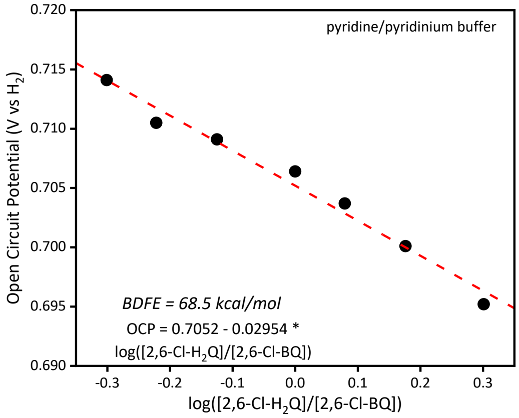

Figure S21. Open-circuit potential at different ratios of 2,6-Cl$_2$-H$_2$Q/2,6-Cl$_2$-BQ, plotted against the log ratios of the substrates. Data collected in pyridine/pyridinium buffer.

4-MeO-2,6-DTBP/4-MeO-2,6-DTBP·

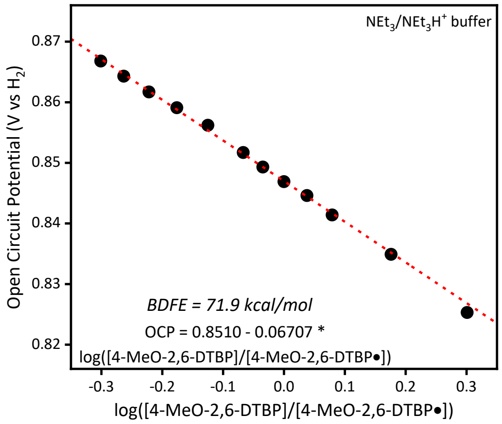

Figure S22. Open-circuit potential at different ratios of 4-MeO-2,6-DTBP/4-MeO-2,6-DTBP·, plotted against the log ratios of the substrates. Data collected in NEt_3/NEt_3H^+ buffer.

2,4,6-TTBP/2,4,6-TTBP·

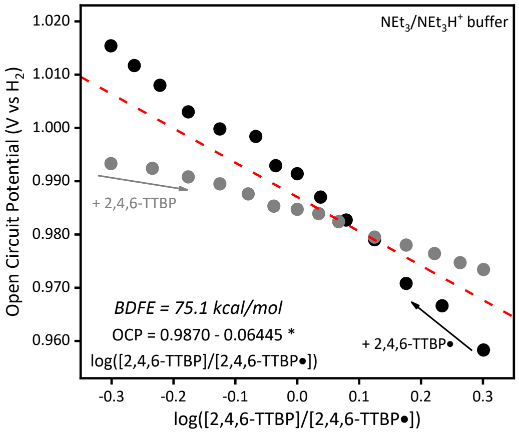

Figure S23. Open-circuit potential at different ratios of 2,4,6-TTBP/2,4,6-TTBP·, plotted against the log ratios of the substrates. Data collected in NEt_3/NEt_3H^+ buffer. Note: in this measurement errors from reference electrode drift[4] were eliminated by fitting the data from titrations in both directions.

5. Reactivity and of ECPBs toward PCET substrates.

General procedures:

NMR experiments: Reactions between 1 mM $^{\mathbf{MeO-H}}\mathbf{5^+}$/$^{\mathbf{Me-H}}\mathbf{5^+}$/$\mathbf{5^+}$ and stoichiometric amounts of PCET substrates ($^{\mathbf{X-R}}\mathbf{5^+}$ + m XH_n → $^{\mathbf{X-R}}\mathbf{1H_4^+}$ + m X, m, n = 1, 2) were performed at 1 mL scale. In the glovebox, 0.025 mL of 40 mM $^{\mathbf{MeO-H}}\mathbf{5^+}$/$^{\mathbf{Me-H}}\mathbf{5^+}$/$\mathbf{5^+}$ solution, 0.025 mL 40 mM internal standard (hexamethylbenzene or 1,3,5-trimethoxybenzene), and 0.025 mL of 80/160 mM PCET substrates were added into 0.925 mL DMF-d_7 separately, mixed and transferred into a 7-inch, 5-mm o.d. NMR tube. NMRs spectra were recorded after 10 minutes (immediately the samples were prepared) and until reactions reached completion as determined. Reaction between 1 mM $^{\mathbf{MeO-H}}\mathbf{1H_4^+}$ and 2 mM 1,4-NQ was conducted in the same fashion, just using 0.025 mL 40 mM **[Cu(MeCN)$_4$]PF$_6$** and 0.025 mL 80 mM $^{\mathbf{MeO-H}}\mathbf{LH_2}$ instead of $^{\mathbf{MeO-H}}\mathbf{5^+}$.

UV-Vis experiments: Reactions between 1 mM $^{\mathbf{MeO-H}}\mathbf{5^+}$/$^{\mathbf{Me-H}}\mathbf{5^+}$/$\mathbf{5^+}$ and stoichiometric PCET substrates ($^{\mathbf{X-R}}\mathbf{5^+}$ + m XH_n → $^{\mathbf{X-R}}\mathbf{1H_4^+}$ **+** m X, m, n = 1, 2) were performed at 1 mL scale. 0.2 mL of 5 mM $^{\mathbf{MeO-H}}\mathbf{5^+}$/$^{\mathbf{Me-H}}\mathbf{5^+}$/$\mathbf{5^+}$ solution and 0.1 mL of 20/40 mM PCET substrates were added into 0.7 mL DMF in a 2-mL vial. The reactions were kept in the glovebox for 1–8 hours. At the end of reactions, the solutions were diluted to 0.05 mM (0.15 mL into 2.85 mL DMF) into a 10 mm path quartz cell, after which UV-vis spectra were taken and concentration of $^{\mathbf{MeO-H}}\mathbf{5^+}$/$^{\mathbf{Me-H}}\mathbf{5^+}$/$\mathbf{5^+}$ were calculated. Blank experiments (containing no PCET substrates) were also prepared and measured for comparison. The reactions between 1 mM $^{\mathbf{MeO-H}}\mathbf{1H_4^+}$/$^{\mathbf{Me-H}}\mathbf{1H_4^+}$/$\mathbf{1H_4^+}$ and PCET substrates ($^{\mathbf{X-R}}\mathbf{1H_4^+}$ + m X → $^{\mathbf{X-R}}\mathbf{5^+}$ + m XH_n , m, n = 1, 2) were carried out in the same fashion, in which $^{\mathbf{X-R}}\mathbf{1H_4}$ were generated in-situ by mixing $^{\mathbf{X-R}}\mathbf{LH_2}$ and **[Cu(MeCN)$_4$]PF$_6$** at 2:1 ratio.

Calculation of the equilibrium constants from NMR experiments:

$$K_{eq} = [^{X\text{-}R}\mathbf{1H_4^+}][X]^m/([^{X\text{-}R}\mathbf{5^+}][XH_n]^m)$$

Calculation of the equilibrium constants from UV-vis experiments:

$$^{X\text{-}R}5^+ \;+\; 2\,XH_2 \;\xrightleftharpoons{K_{eq.}}\; ^{X\text{-}R}1H_4^{\;+} \;+\; 2\,X$$

	$^{X-R}5^+$	2 XH_2	$^{X-R}1H_4^+$	2 X
Initial:	1 mM	2 mM	0	0
End:	n	2n	1 - n	2 - 2n

Equilibrium constant ($K_{eq.}$) can be calculated from the given equation:

$$K_{eq.} = \frac{(1-n)\times(2-2n)^2}{n\times(2n)^2} = \frac{(1-n)^3}{n^3}$$

In the equation above, n is the concentration of $^{\mathbf{X-R}}\mathbf{5^+}$ determined by UV-vis.

Calculation of free energies (ΔG in kcal/mol):

$$\Delta G_{calc.} = -RT\ln K_{eq}$$
$$R = 1.9872\ \text{cal/(K mol)};\ T = 293.15\ \text{K}$$

Table S4. Reactivity of oxidized Cu-based ECPBs ($^{X-R}$**5**$^{+}$) towards PCET reagents in DMF/DMF-d_7 at room temperature.

PCET couples	E^o (V)	$^{MeO-H}$**5**$^{+}$→$^{MeO-H}$**1**H_4^+		$^{Me-H}$**5**$^{+}$→$^{Me-H}$**1**H_4^+		**5**$^{+}$→**1**H_4^+	
		ΔG	*Reaction*	*ΔG*	*Reaction*	*ΔG*	*Reaction*
1,8-Cl_2-H_2AQ → 1,8-Cl_2-AQ	0.169	-29.2	√	-42.0	√	-56.4	√
DHP → phenazine	0.269	-20.0	√	-32.8	√	-47.2	√
1,4-H_2NQ → 1,4-NQ	0.500	1.2	K_{eq} = 0.46	-11.6	√	-26.0	√
2,6-$(MeO)_2$-H_2Q → 2,6-$(MeO)_2$-BQ	0.514	2.4	K_{eq} = 0.08	-10.4	√	-24.8	√
TEMPOH → TEMPO[a]	0.582	8.8	×	-4.0	$K_{eq} = 10^4$	-18.4	√
2,6-Me_2-H_2Q → 2,6-Me_2-BQ[b]	0.592	10.0	×	-2.8	K_{eq} = 7.0	-17.2	√
1,4-H_2Q → 1,4-BQ	0.708	20.4	×	7.6	×	-6.8	√
2,6-Cl_2-H_2Q → 2,6-Cl_2-BQ	0.724	21.6	×	8.8	×	-5.6	K_{eq} = 55
4-MeO-2,6-DTBP → 4-MeO-2,6-DTBP·	0.851	33.6	×	20.8	×	6.4	$K_{eq} = 10^{-6}$
2,4,6-TTBP → 2,4,6-TTBP·	0.987	46.4	×	33.6	×	19.2	×

Table S5. Equilibrium constants and free energies for reactions of $^{X-R}$**5**$^{+}$/$^{X-R}$**1**H_4^+ with PCET couples (X/XH_n).

PCET substrate	E^o (V)	BDFE (kcal/mol)	*Equilibrium Constant*	ΔG_{OCP}	$\Delta G_{exp.}$
$^{MeO-H}$**5**$^{+}$ → $^{MeO-H}$**1**H_4^+					
1,4-H_2NQ → 1,4-NQ	0.500	63.8	K_{eq} = 0.46	1.2	0.5
2,6-$(MeO)_2$-H_2Q → 2,6-$(MeO)_2$-BQ	0.514	64.1	K_{eq} = 0.08	2.4	1.5
$^{Me-H}$**5**$^{+}$ → $^{Me-H}$**1**H_4^+					
TEMPOH → TEMPO[a]	0.582	65.7	$K_{eq} = 10^4$	-4.0	-5.5
2,6-Me_2-H_2Q → 2,6-Me_2-BQ	0.592	66.0	K_{eq} = 7.0	-2.8	-1.1
**5**$^{+}$ → **1**H_4^+					
2,6-Cl_2-H_2Q → 2,6-Cl_2-BQ	0.724	68.9	K_{eq} = 55	-5.6	-2.3
4-MeO-2,6-DTBP → 4-MeO-2,6-DTBP·	0.851	71.9	$K_{eq} = 10^{-6}$	6.4	8.0

5.1 Reactivity determined by NMR:

$^{MeO\text{-}H}5^+$ + 1,8-Cl_2-H_2AQ

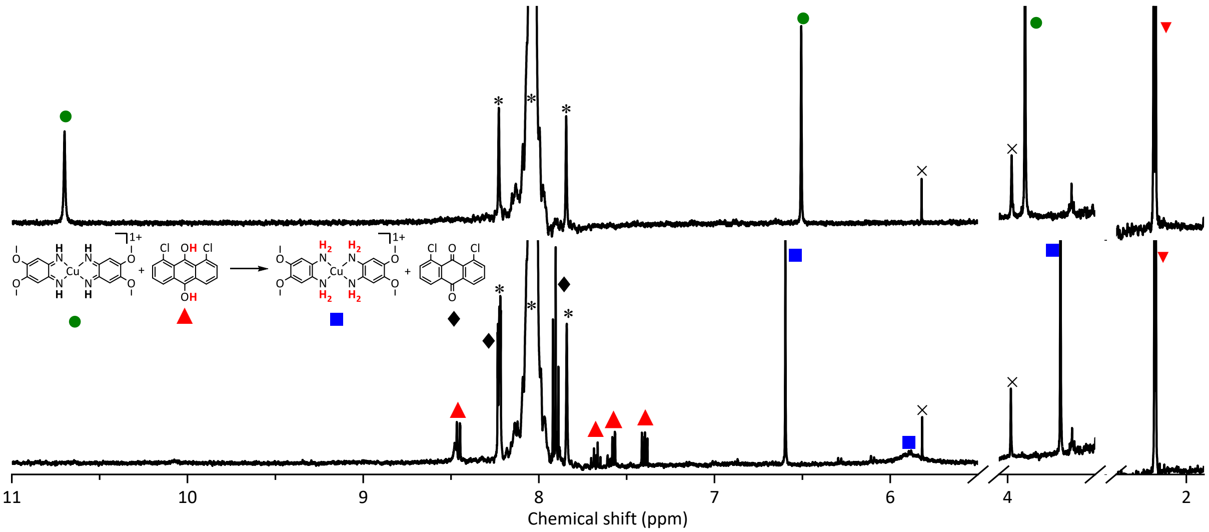

Figure S24. NMR spectra of $^{MeO\text{-}H}5^+$ + 1,8-Cl_2-H_2AQ in DMF-d_7 at room temperature (top: $^{MeO\text{-}H}5^+$ in DMF-d_7; bottom: 10 minutes after reaction with ~2.5 equivalent 1,8-Cl_2-H_2AQ). Note: peaks are assigned as noted in the inset; ▼hexamethylbenzene (1 mM, internal standard); *DMF peaks; ×unknown impurities (concentration didn't change).

$^{MeO\text{-}H}5^+$ + 2,6-$(MeO)_2$-H_2Q

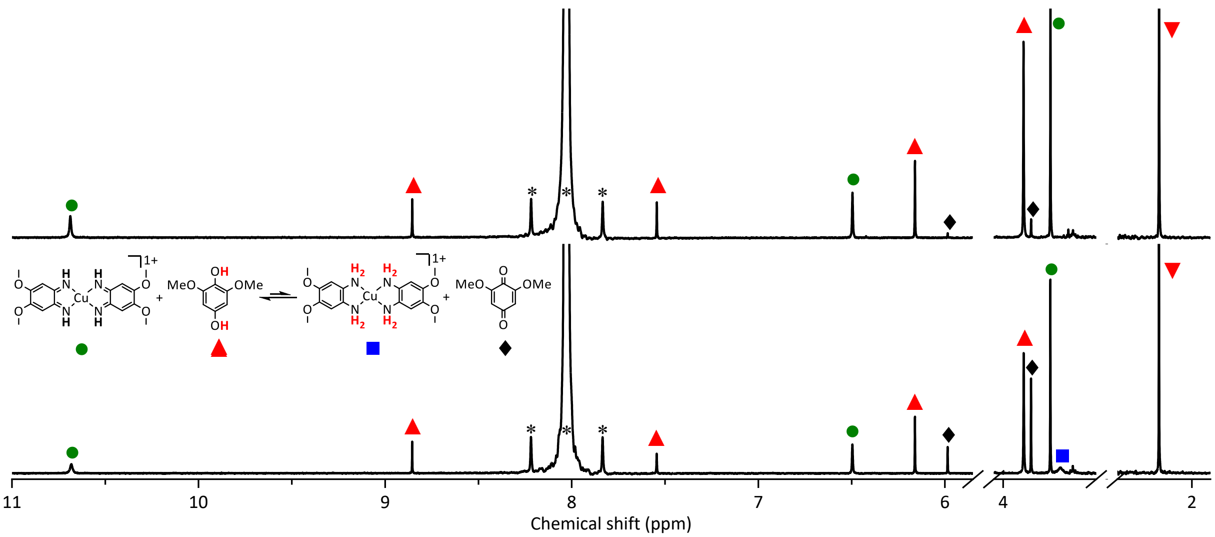

Figure S25. NMR spectra of $^{MeO\text{-}H}5^+$ + 2,6-$(MeO)_2$-H_2Q in DMF-d_7 at room temperature (top: 10 minutes after reaction with ~2 equivalent 2,6-$(MeO)_2$-H_2Q; bottom: after 24 h). Note: peaks are assigned as noted in the inset; ▼hexamethylbenzene (1 mM, internal standard); *DMF peaks.

$^{MeO\text{-}H}$5$^+$ + 1,4-H$_2$NQ

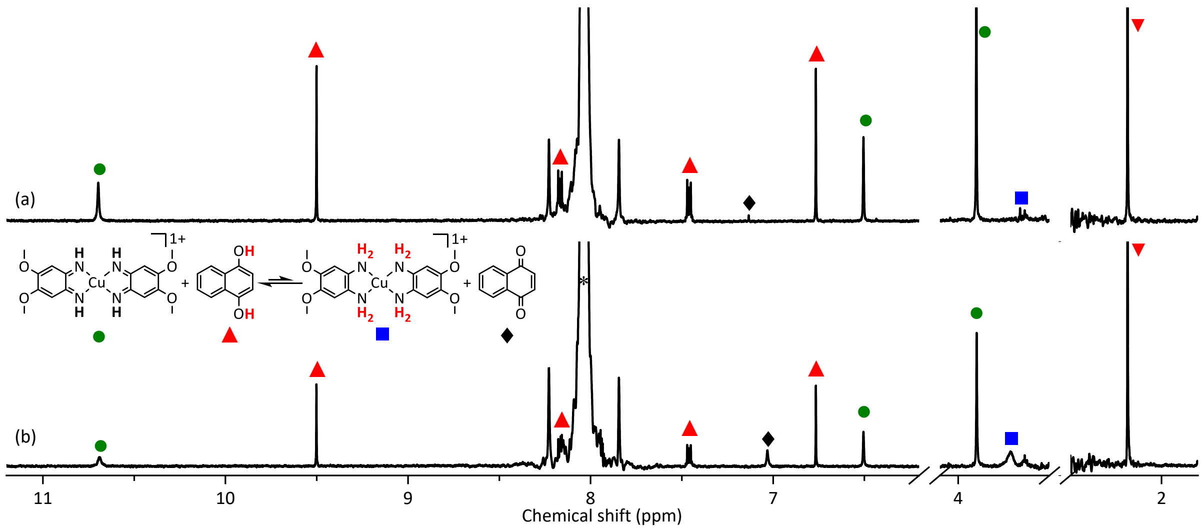

Figure S26. NMR spectra of $^{MeO\text{-}H}$5$^+$ + 1,4-H$_2$NQ in DMF-d$_7$ at room temperature (top: 10 minutes after reaction with ~2 equivalent 1,4-H$_2$NQ; bottom: after 24 h). Note: peaks are assigned as noted in the inset; ▼hexamethylbenzene (1 mM, internal standard); *DMF peaks.

$^{Me\text{-}H}$5$^+$ + TEMPOH

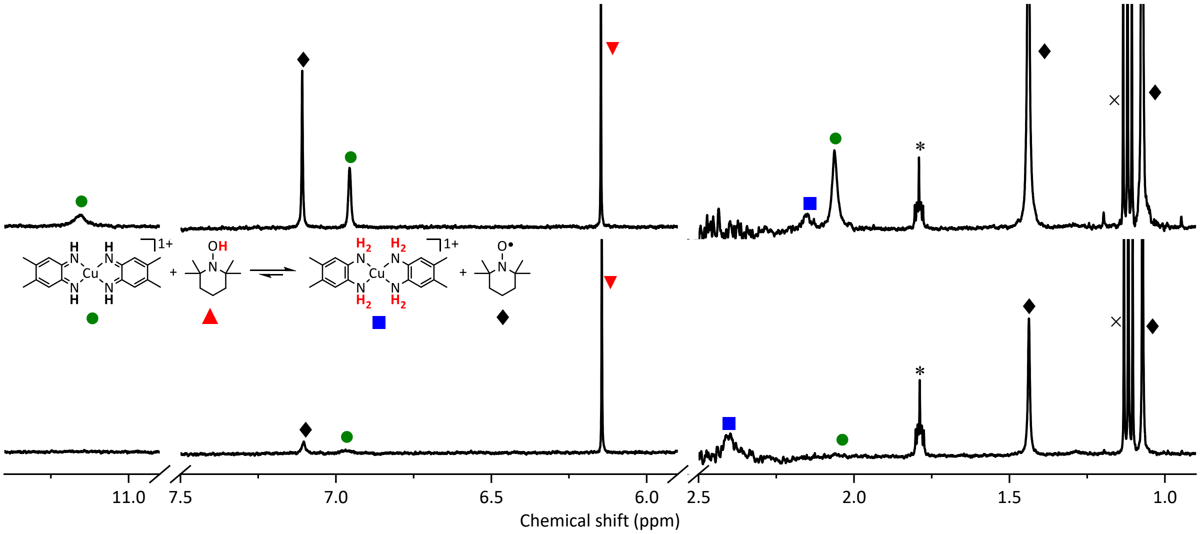

Figure S27. NMR spectra of $^{Me\text{-}H}$5$^+$ + TEMPOH in DMF-d$_7$ at room temperature (top: 10 minutes after reaction with ~4 equivalent TEMPOH; bottom: after 12 hours). Note: peaks are assigned as noted in the inset; ▼1,3,5-trimethoxybenzene (1 mM, internal standard); *Et$_2$O; change in the concentration of TEMPOH was used as concentration of TEMPO at equilibrium.

$^{\text{Me-H}}\mathbf{5}^{+}$ + 2,6-Me$_2$-H$_2$Q

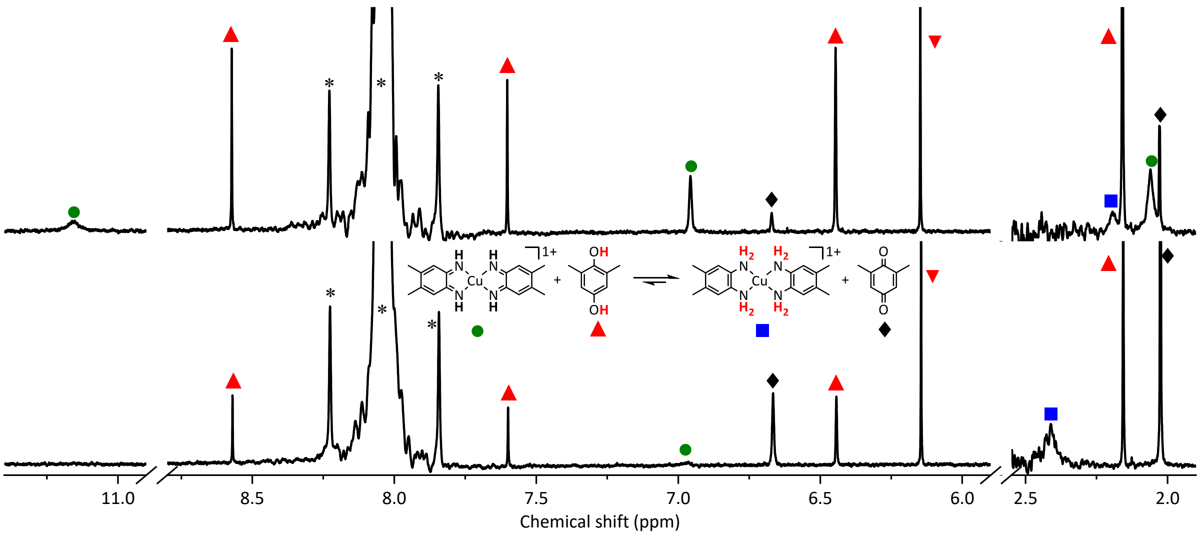

Figure S28. NMR spectra of $^{\text{Me-H}}\mathbf{5}^{+}$ + 2,6-Me$_2$-H$_2$Q in DMF-d$_7$ at room temperature (top: 10 minutes after reaction with ~4 equivalent TEMPOH; bottom: after 12 hours). Note: peaks are assigned as noted in the inset; ▼1,3,5-trimethoxybenzene (1 mM, internal standard); *DMF peaks.

$\mathbf{5}^{+}$ + 2,6-Cl$_2$-H$_2$Q

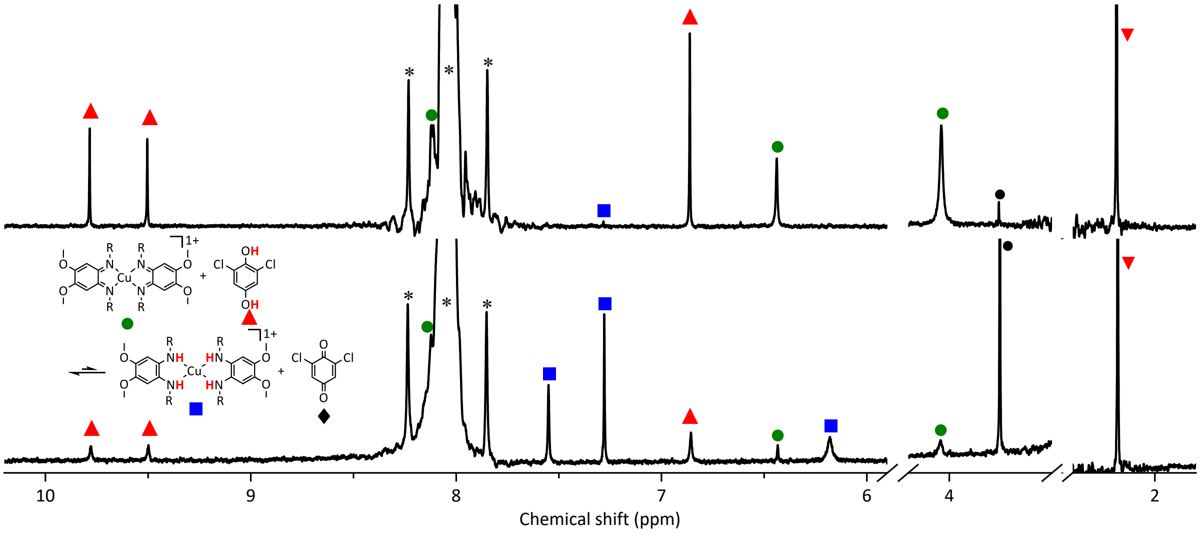

Figure S29. NMR spectra of $\mathbf{5}^{+}$ + 2,6-Cl$_2$-H$_2$Q in DMF-d$_7$ at room temperature (top: 10 minutes after reaction with ~2 equivalent 2,6-Cl$_2$-H$_2$Q; bottom: after 24 h). Note: peaks are assigned as noted in the inset; ▼hexamethylbenzene (1 mM, internal standard); *DMF peaks; 2,6-Cl$_2$-BQ peak overlap with DMF peak at ~ 8.00 ppm, change in the concentration of 2,6-Cl$_2$-H$_2$Q was used as concentration of 2,6-Cl$_2$-BQ at equilibrium.

5⁺ + 4-MeO-2,6-DTBP

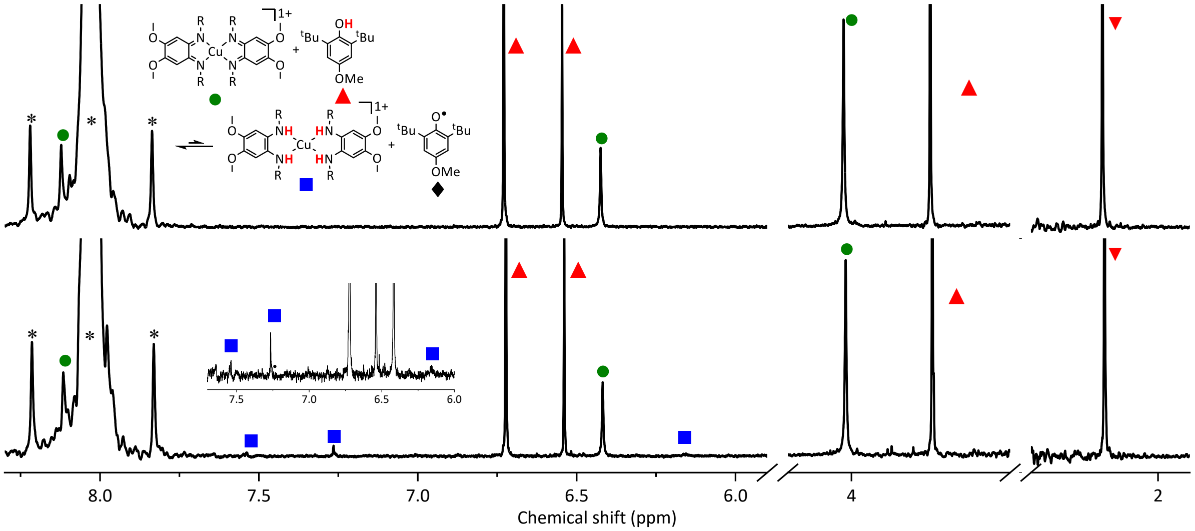

Figure S30. NMR spectra of **5⁺** + 4-MeO-2,6-DTBP in DMF-d7 at room temperature (top: 10 minutes after reaction with ~4 equivalent 4-MeO-2,6-DTBP; bottom: after 24 h). Note: peaks are assigned as noted in the inset; ▾hexamethylbenzene (1 mM, internal standard); *DMF peaks; concentration of $^{\mathbf{MeO\text{-}tBu}}\mathbf{1H_4^+}$ was used to calculate the concentration of 4-MeO-2,6-DTBP radical at equilibrium; 4-MeO-2,6-DTBP radical were observed and quantified by UV-vis ([radical] = 0.17 mM), giving approximately the same result as calculated above.

5.2 Reactivity determined by UV-vis:

$^{MeO\text{-}H}$*5*$^{+}$ *+ PCET reagents*

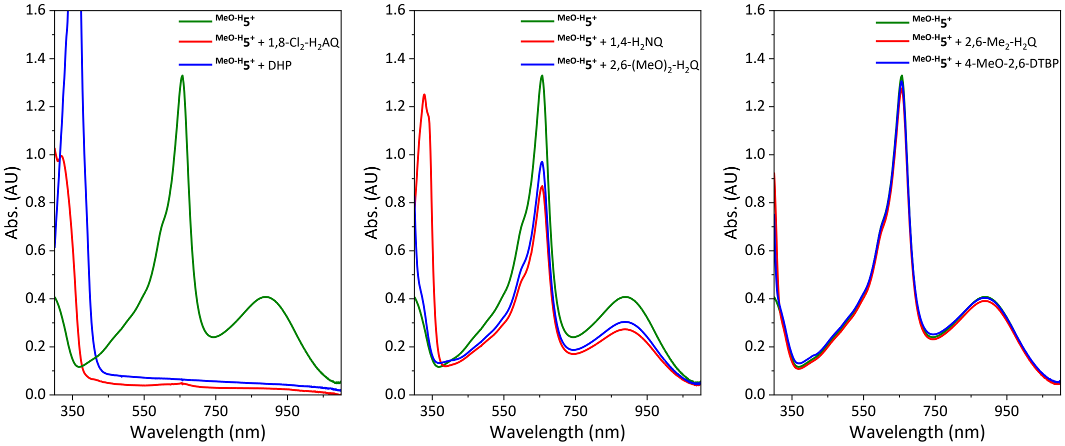

Figure S31. Reactions of $^{MeO\text{-}H}$**5**$^{+}$ towards PCET substrates with lower BDFEs (left, fully react), similar BDFEs (middle, equilibrate) and higher BDFEs (right, not react). Note: other substrates with higher BDFEs listed in table S4 are not shown for clarity.

$^{Me\text{-}H}$*5*$^{+}$ *+ PCET reagents*

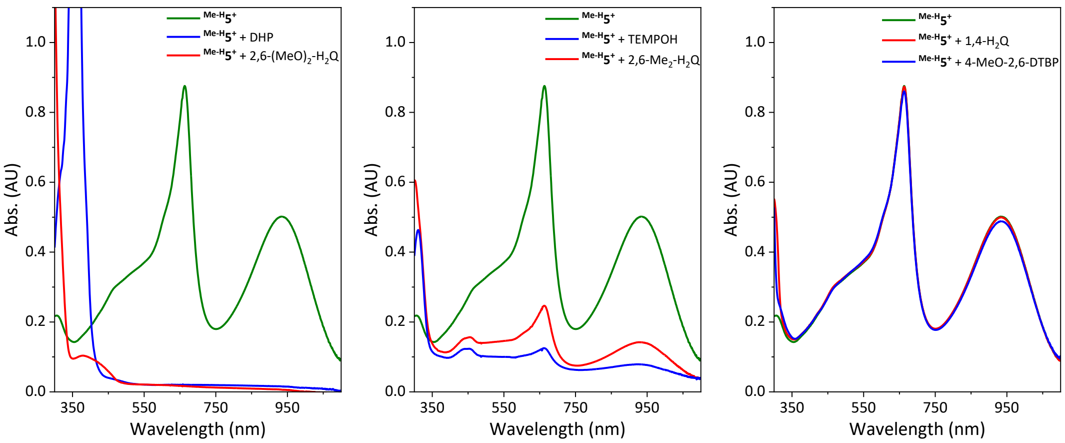

Figure S32. Reactions of $^{Me\text{-}H}$**5**$^{+}$ towards PCET substrates with lower BDFEs (left, fully react), similar BDFEs (middle, equilibrate) and higher BDFEs (right, not react). Note: other substrates with lower BDFEs or higher BDFEs listed in table S4 are not shown for clarity.

5^+ + PCET reagents

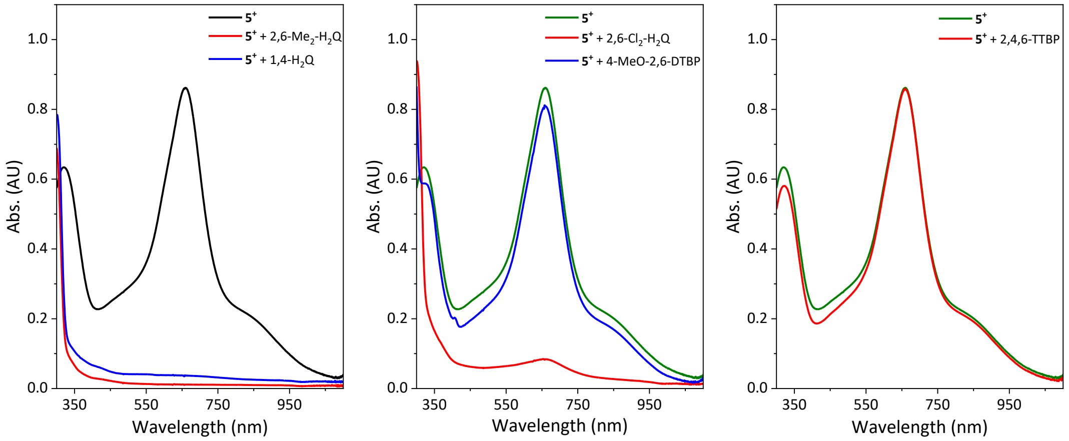

Figure S33. Reactions of $\mathbf{5^+}$ towards PCET substrates with lower BDFEs (left, fully react), similar BDFEs (middle, equilibrate) and higher BDFEs (right, not react). Note: other substrates with lower BDFEs listed in table S4 are not shown for clarity; formation of 4-MeO-2,6-DTBP radical was observed at 408 nm by UV-vis (middle, blue spectrum); no 2,4,6-TTBP radical was observed at 403 nm by UV-vis (right, red spectrum).

6. Decoupled $4H^+/4e^-$ reduction/dehydrogenation reactivity

General procedure: In the glovebox, 1 mL DMF-d_7 solution containing 10 mM 1,3,5-trimethoxybenzene, ~20 mM $^{cat}LH_4$ and 0.5 equiv of $[Cu^I(CH_3CN)_4]PF_6$ was prepared. After the first NMR spectrum was recorded, O_2 was bubbled into the solution for 15 seconds. Then NMR measurements were recorded over time. After full consumption of ^{PR}ECPB the solution was degassed, transferred into the glovebox and reacted with slightly excess 2,6-$(MeO)_2$-H_2Q. After completion of the reaction (regeneration of complex $\mathbf{1H_4^+}$), the solution was once again reacted with O_2. These oxidative and reductive cycles were repeated multiply times.

Table S6. Quantification of NMR quantification of the decoupled reaction of **ECPB** ($\mathbf{1H_4^+/5^+}$) with O_2 and 2,6-$(MeO)_2$-H_2Q at room temperature.

Cycles	$[1H_4^+]$ (mM)	$[5^+]$ (mM)	Mass Balance
Start	9.00	0.00	100.0%
Oxidation 1	0.63	8.13	97.2%
Reduction 1	8.75	0.00	97.2%
Oxidation 2	0.08	7.50	84.2%
Reduction 2	7.50	0.00	83.3%
Oxidation 3	0.20	5.83	66.9%
Reduction 3	6.05	0.00	67.2%
Oxidation 4	0.68	4.50	57.5%
Reduction 4	5.23	0.00	58.1%

Table S7. Quantification of NMR quantification of the decoupled reaction of **ECPB** ($\mathbf{^{Me\text{-}H}1H_4^+/\ ^{Me\text{-}H}5^+}$) with O_2 and 2,6-$(MeO)_2$-H_2Q at room temperature.

Cycles	$[^{Me\text{-}H}1H_4^+]$ (mM)	$[^{Me\text{-}H}5^+]$ (mM)	Mass Balance
Start	12.50	0.00	100.0%
Oxidation 1	0.00	11.75	94.0%
Reduction 1	11.48	0.00	91.9%
Oxidation 2	0.00	11.13	89.0%
Reduction 2	11.00	0.00	88.0%
Oxidation 3	0.00	10.08	80.6%
Reduction 3	10.08	0.00	80.7%
Oxidation 4	0.00	9.20	73.6%
Reduction 4	9.08	0.00	72.7%
Oxidation 5	0.00	8.38	67.0%
Reduction 5	8.28	0.00	66.2%
Oxidation 6	0.00	7.88	63.0%
Reduction 6	7.83	0.00	62.7%
Oxidation 7	0.00	7.53	60.2%
Reduction 7	7.50	0.00	60.0%
Oxidation 8	0.00	7.33	58.6%
Reduction 8	7.25	0.00	58.0%

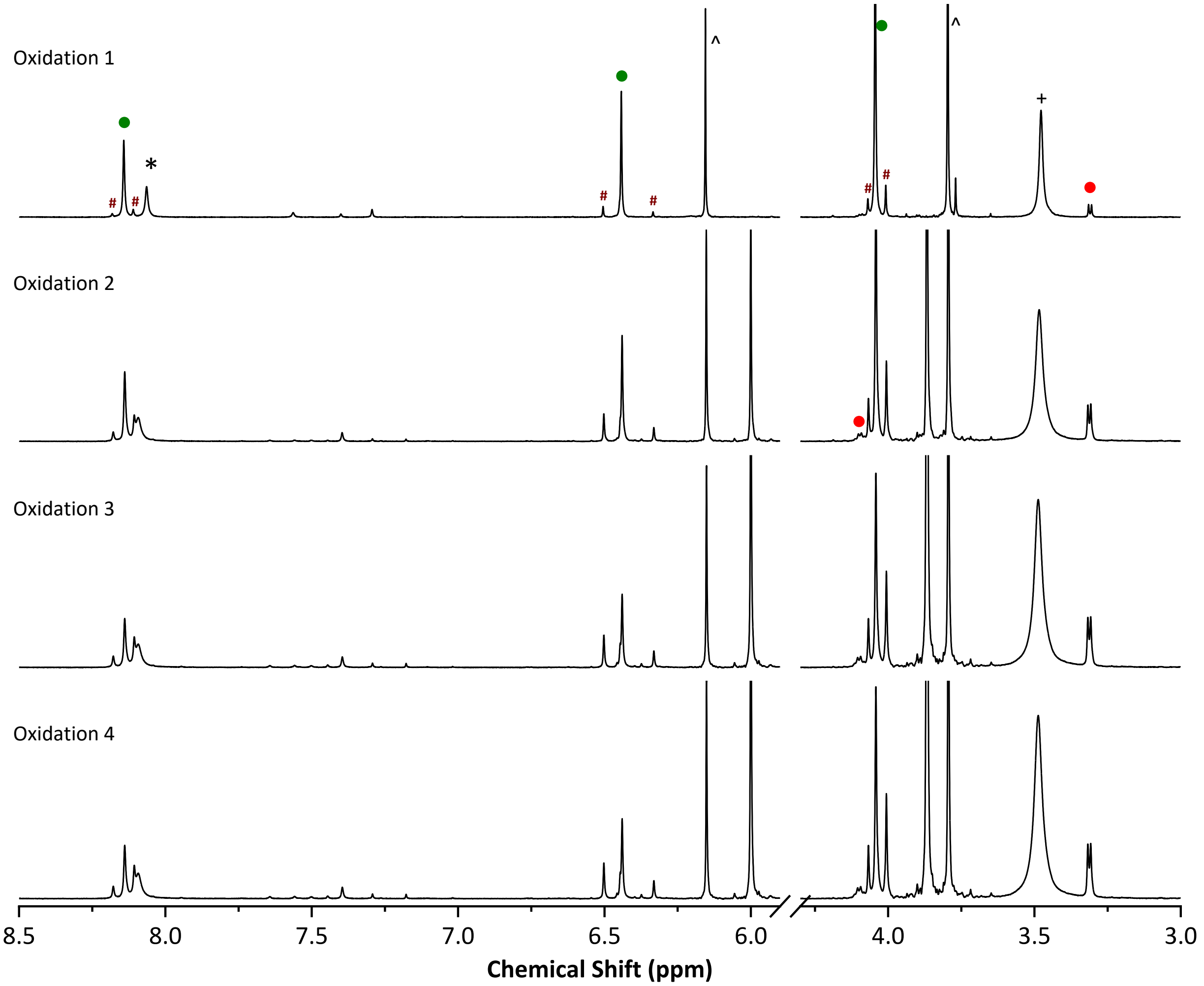

Figure S34. NMR spectra of the decoupled reaction of **ECPB** (**$1H_4^+$**/ **5^+**) with O_2 and 2,6-(MeO)$_2$-H_2Q after each oxidation in DMF-d$_7$ at room temperature. Green dot: **5^+**; red dot: MeOH (NMR in DMF-d$_7$: 3.30 ppm, d, 3H, CH_3; 4.10 ppm, q, 1H, O-H) ^: internal standard, 1,3,5-trimethoxybenzene; #: degradation product of **5^+** with lower symmetry; +: H_2O/HDO; *: DMF; peaks at 6.00 and 3.79 ppm belong to 2,6-(MeO)$_2$-PhOH.

7. BDFE analysis

7.1 pK_a determination for the protonation of $^{X\text{-}R}$4 to $^{X\text{-}R}$4H$^+$ (followed by disproportionation)

General procedure: In the glovebox, 3 mL of $^{X\text{-}R}$**5$^+$** solution (0.0625 mM) in DMF were placed in a 10 mm path quartz cell capped with a rubber septum. Monitored by UV-vis at -40 °C under Ar, $^{X\text{-}R}$**4** was generated by reduction of $^{X\text{-}R}$**5$^+$** by ~1 equiv cobaltocene, followed by addition of 50 equiv proton sources with known pK_a values (in table S8). Protonation of $^{X\text{-}R}$**4** to $^{X\text{-}R}$**4H$^+$** was monitored by UV-vis.

Table S8. Summary of the pK_a of acids used in the section and their reactivity with $^{X\text{-}R}$**4**.

Acid	pK_a (in DMF)	$^{MeO\text{-}H}$4→$^{MeO\text{-}H}$4H$^+$	$^{Me\text{-}H}$4→$^{Me\text{-}H}$4H$^+$	4→4H$^+$
pyridinium	3.3[6]	React	React	React
3-Cl-PhOH	16.3[7]	n.d.	n.d.	React
4-Cl-PhOH	16.8[7]	React	React	Not React
PhOH	18.4[7]	n.d.	React	n.d.
4-F-PhOH	18.8[a]	n.d.	React	Not React
4-MeO-PhOH	19.9[a]	n.d.	Partially React	Not React
diphenylurea	20.6[7]	React	Partially React	n.d.
fluorene	23.3[a]	Partially React	Not React	n.d.
CF_3CH_2OH	24.2[a]	Partially React	Not React	n.d.
CH_3CH_2OH	30.2[a]	Not React	n.d.	n.d.

[a]pK_a values were converted from DMSO[7] (pK_a(DMF) = 0.96pK_a(DMSO) + 1.6); N.D.: not determined.

Reduction of $^{\text{MeO-H}}$5^{+} and protonation of $^{\text{MeO-H}}$4

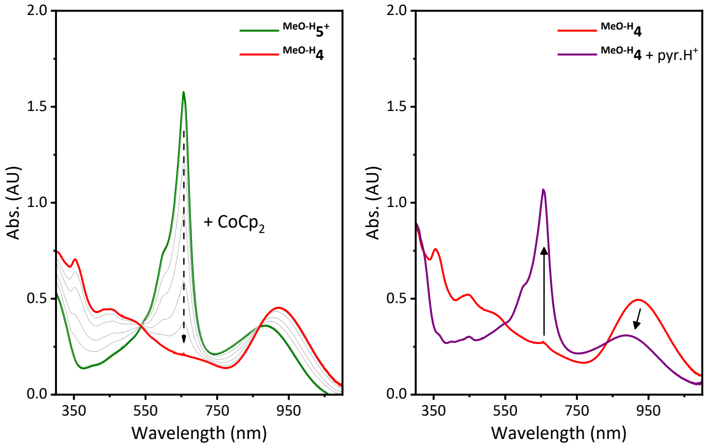

Figure S35. UV-vis spectra of reduction of $^{\text{MeO-H}}$**5**$^{+}$ to $^{\text{MeO-H}}$**4** using stoichiometric amount of $CoCp_2$ (left, 1.2 equiv $CoCp_2$ was used to reduce $^{\text{MeO-H}}$**5**$^{+}$ to $^{\text{MeO-H}}$**4**) and protonation of $^{\text{MeO-H}}$**4** using 1 equiv pyridinium triflate (right, protonation was followed by fast disproportionation to regenerate ~ 0.75 equivalent $^{\text{MeO-H}}$**5**$^{+}$).

Protonation of $^{\text{MeO-H}}$4 using different proton sources

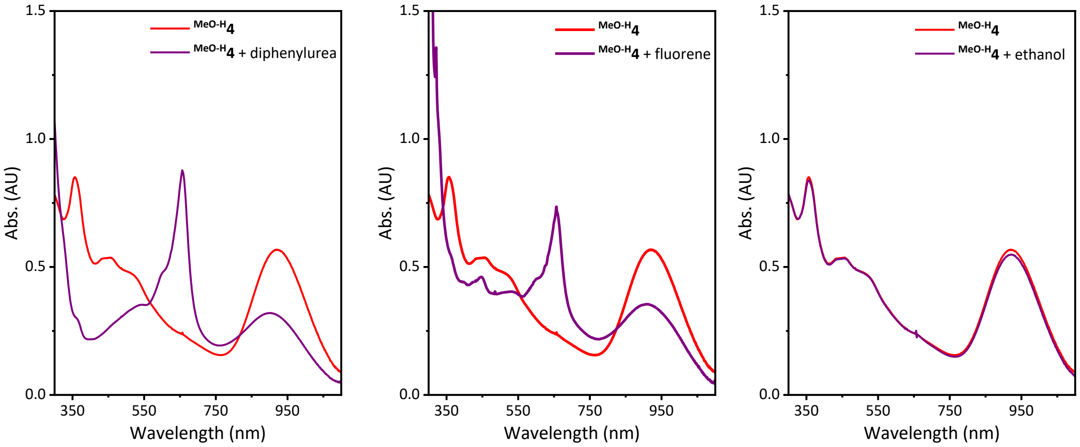

Figure S36. Protonation of $^{\text{MeO-H}}$**4** using acids with known pK_a values (left: diphenylurea, pK_a = 20.6; middle: fluorene, pK_a = 23.2; right: ethanol, pK_a = 30.2) in DMF at -40°C. Note: $^{\text{MeO-H}}$**4** was protonated by diphenylurea, partially protonated by fluorene, and not protonated by ethanol, indicating the pK_a of protonation of $^{\text{MeO-H}}$**4** to $^{\text{MeO-H}}$**4H**$^{+}$ is around 20.

Reduction of $^{Me\text{-}H}5^{+}$ and protonation of $^{Me\text{-}H}4$

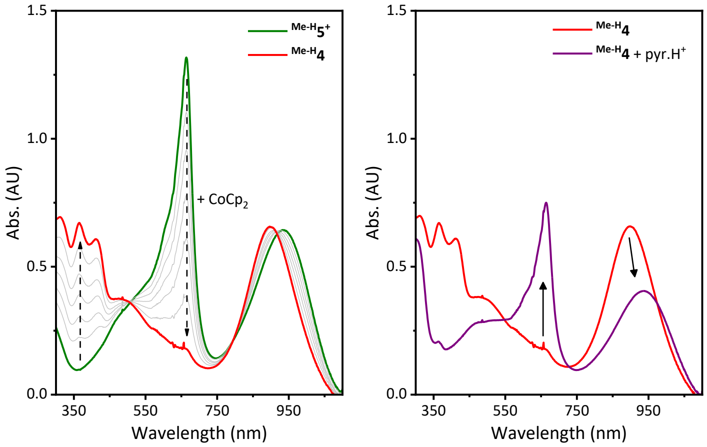

Figure S37. UV-vis spectra of reduction of $^{Me\text{-}H}\mathbf{5}^{+}$ to $^{Me\text{-}H}\mathbf{4}$ using stoichiometric amount of $CoCp_2$ (left, 1.2 equiv $CoCp_2$ was used to reduce $^{Me\text{-}H}\mathbf{5}^{+}$ to $^{Me\text{-}H}\mathbf{4}$) and protonation of $^{Me\text{-}H}\mathbf{4}$ using 1 equiv pyridinium triflate (right, protonation was followed by fast disproportionation to regenerate ~ 0.75 equivalent $^{Me\text{-}H}\mathbf{5}^{+}$).

Protonation of $^{Me\text{-}H}4$ using different proton sources

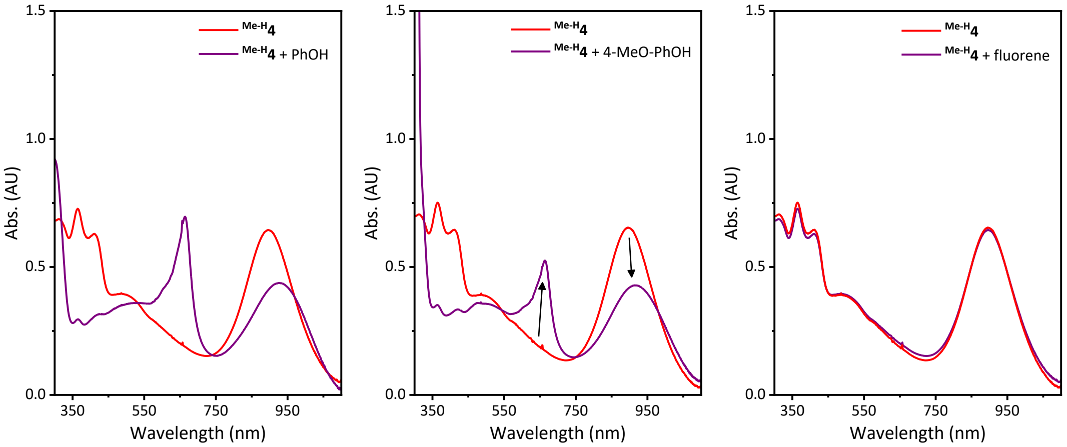

Figure S38. Protonation of $^{Me\text{-}H}\mathbf{4}$ using acids with known pK_a values (left: PhOH, pK_a = 18.4; middle: 4-MeO-PhOH, pK_a = 19.8; right: fluorene, pK_a = 22.6) in DMF at -40°C. Note: $^{Me\text{-}H}\mathbf{4}$ was protonated by PhOH, partially protonated by 4-MeO-PhOH, and not protonated by fluorene, indicating the pK_a of protonation of $^{Me\text{-}H}\mathbf{4}$ to $^{Me\text{-}H}\mathbf{4H}^{+}$ is around 18.

7.2. BDFE calculation of 1H$^+$/1e$^-$ reduction of $^{X\text{-}R}$5$^+$ to $^{X\text{-}R}$4H$^+$:

BDFE of this 1H$^+$/1e$^-$ step was calculated by Bordwell equation:

$$\mathbf{BDFE = 23.06E_{1/2} + 1.37p\mathit{K}_a + 67.6}$$

$E_{1/2}$ was determined by cyclic voltammetry (see section 3), and pK_a was determined above.

BDFE ($^{MeO\text{-}H}$5$^+$/$^{MeO\text{-}H}$4H$^+$) = 23.06 x (-1.18) + 1.37 x 20 + 67.6 = **67.8 kcal/mol**
BDFE ($^{Me\text{-}H}$5$^+$/$^{Me\text{-}H}$4H$^+$) = 23.06 x (-0.87) + 1.37 x 18 + 67.6 = **72.2 kcal/mol**

7.3. Calculation for free energies of disproportionation and ligand-exchange reactions.

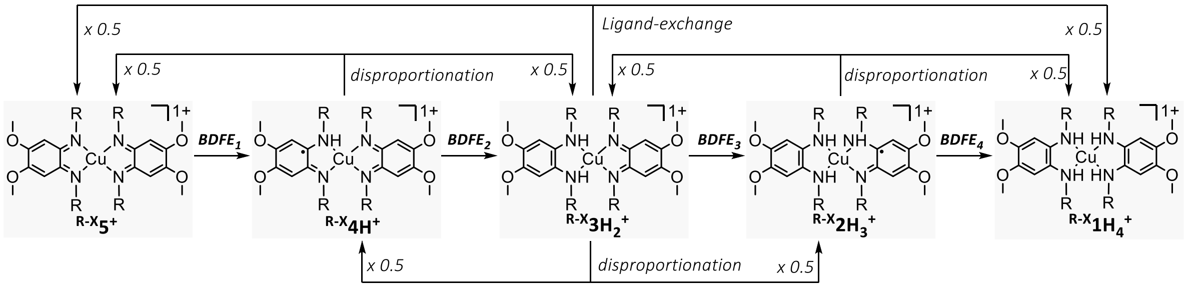

Figure S39. Disproportionation and ligand-exchange reactions of ECPB system.

Disproportionation reactions

$^{R\text{-}X}4H^+ \longrightarrow 0.5\ ^{R\text{-}X}5^+ + 0.5\ ^{R\text{-}X}3H_2^+$

$^{R\text{-}X}3H_2^+ \longrightarrow 0.5\ ^{R\text{-}X}4H^+ + 0.5\ ^{R\text{-}X}2H_3^+$

$^{R\text{-}X}2H_3^+ \longrightarrow 0.5\ ^{R\text{-}X}3H_2^+ + 0.5\ ^{R\text{-}X}1H_4^+$

$0.5\ ^{R\text{-}X}4H^+ + 0.5\ ^{R\text{-}X}2H_3^+ \longrightarrow 0.5\ ^{R\text{-}X}5^+ + 0.5\ ^{R\text{-}X}1H_4^+$ $\quad \Delta G^0_{disp} = 0.5\ (BDFE_1 - BDFE_4)$

Ligand-exchange reaction

$^{R\text{-}X}3H_2^+ \longrightarrow 0.5\ ^{R\text{-}X}5^+ + 0.5\ ^{R\text{-}X}1H_4^+$ $\quad \Delta G^0_{LE} = 0.5\ (BDFE_1 + BDFE_2 - BDFE_3 - BDFE_4)$

Figure S40. Calculation for free energies of disproportionation and ligand-exchange reactions of ECPB system.

8. Ligand exchange reactions

8.1 Synthesis of $^{CD3O\text{-}tBu}LH_2$

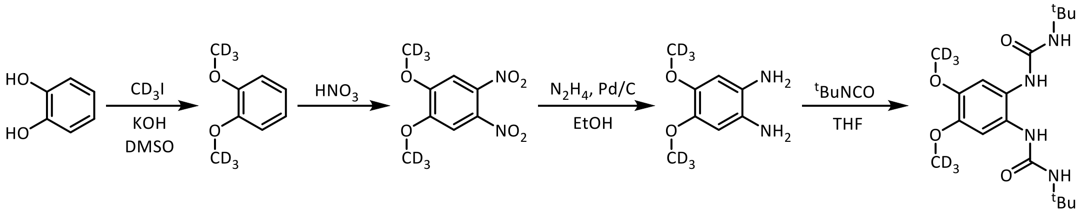

1,2-bis(methoxy-d$_3$)benzene: To a solution of 1,2-dihydroxybenzene (1.5 g, 13.6 mmol) in anhydrous DMSO (10 mL) at room temperature was added KOH (3.06 g, 54.5 mmol) followed by methyl iodide-d$_3$ (2.12 mL, 34.1 mmol) .After being stirred at room temperature overnight., the reaction mixture was diluted with water (40 mL) and extracted with CH_2Cl_2 (4 x 30 mL). The combined organic layers were washed with water (3 x 50 mL), dried over Na_2SO_4, filtered and concentrated under reduced pressure. The residue was dried (vacuum oven) to give yellow oil (2.00 g, containing DCM and H_2O). **^{1}H-NMR(CDCl$_3$)**: 6.67-6.74 ppm (m, 2H, Ph-**H**). The product was directly used in the next step without futher purification.

1,2-bis(methoxy-d$_3$)-4,5-dinitrobenzene: Into a stirring concentrated nitric acid, 2 g of 1,2-bis(methoxy-d$_3$)benzene was added dropwise at 0 ^{o}C. Then the reaction was warmed to room temperature and heated at 80o. After being stirred for 2 hours, the reaction was quenched by precipitation with cold water. The precipitate was collected by filtration, washed with DI water, and dried under reduced pressure, giving the product as faint yellow powder (1.65 g) ^{1}H-NMR(CDCl$_3$): 7.28 ppm (s, 2H, Ph-**H**).

4,5-bis(methoxy-d$_3$)-1,2-phenylenediamine: A 50-mL-round-bottom-flask was charged with 1,2-bis(methoxy-d$_3$)-4,5-dinitrobenzene (1.65 g) and Pd/C under N2 flow, then 20 mL of EtOH and N_2H_4 (1.5 mL) were separately added. The reaction was later elevated to 70 oC and kept for 2 hours (or the solution was no longer yellow) before filtered through celite. The filtrate was reduced to around 4 mL and then stored in freezer overnight. The solid was collected by filtration, washed with cold EtOH and dried under vacuum, giving product as light brown flake (800 mg, 66%). **^{1}H-NMR(DMSO-d$_6$)**: 6.27 ppm (s, 2H, Ph-**H**), 4.08 ppm (s, 4H, N-**H**).

$^{CD3O}LH_2$ (1,1'-(4,5-bis(methoxy-d$_3$)-1,2-phenylene)bis(3-(tert-butyl)urea)): In the glovebox, to a stirring THF solution(3 mL) of 4,5-bis(methoxy-d3)-1,2-phenylenediamine (100 mg, 0.58 mmol) was slowly added ^{t}BuNCO (150 mg, 1.5 mmol) over 1 min. The reaction was stirred for 1 day before being dried. The residue was washed with Et_2O and dried under vacuum to yield white solid (180 mg, 83%). **^{1}H-NMR(DMSO-d$_6$)**: 7.34 ppm (s, 2H, N-**H**), 7.13 ppm (s, 2H, Ph-**H**), 6.16 ppm (s, 2H, N-**H**), 1.29 ppm (s, 18H,tBu-**H**); **^{1}H-NMR(DMF-d$_7$)**: 7.59 ppm (s, 2H, N-**H**), 7.29 ppm (s, 2H, Ph-**H**), 6.21 ppm (s, 2H, N-**H**), 1.34 ppm (s, 18H,tBu-**H**). All NMR peaks match the corresponding peaks of the non-deuterated compound (in both solvents).

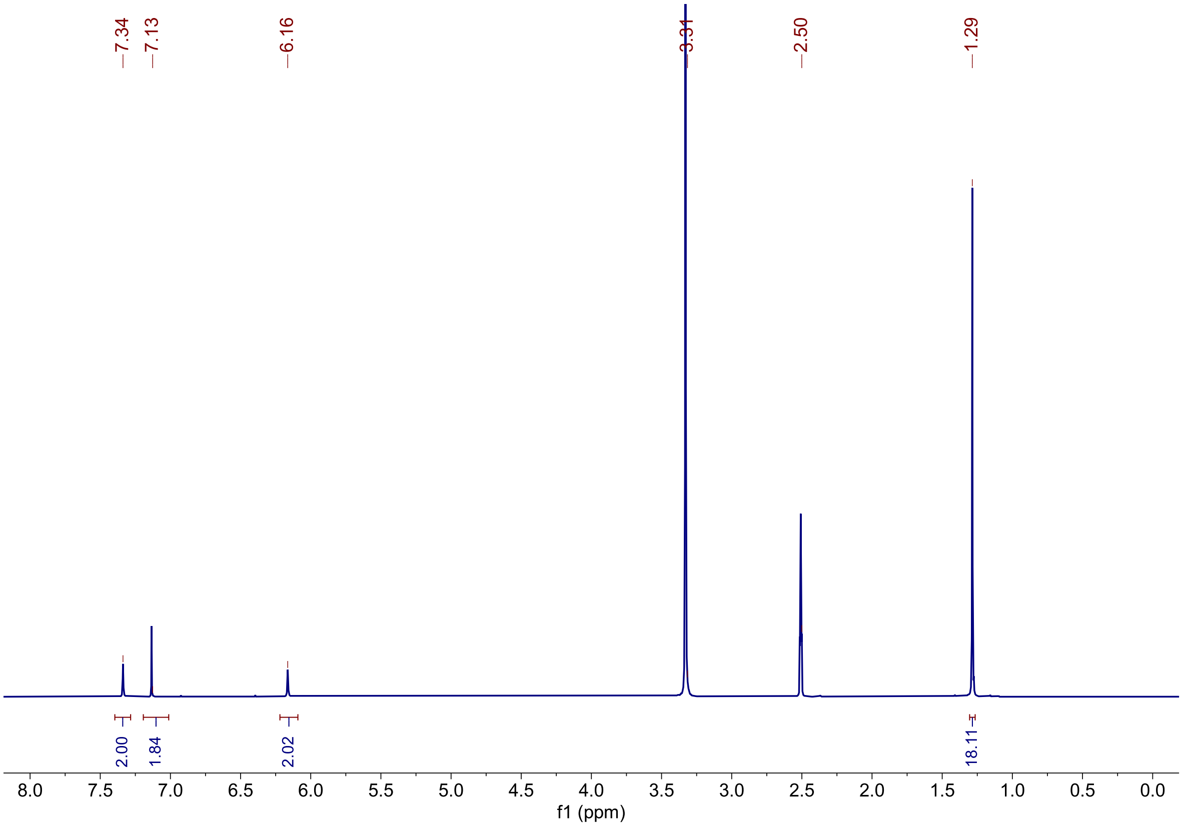

Figure S41. NMR spectrum of $^{CD3O\text{-}tBu}$**LH$_2$** in DMSO-d_6.

8.2. Isoergonic ligand-exchange reaction between 5^+ and $^{D}1H_4^+$

General procedure: In the glovebox, 1 mL DMF-d_7 solution containing 4 mM 1,3,5-trimethoxybenzene, 9 mM $^{CD3O\text{-}tBu}LH_4$, 4.5 mM $[Cu^I(CH_3CN)_4]PF_6$ was prepared. After the first NMR spectrum was recorded, the NMR tube was transferred into the glovebox, and equimolar of **5⁺** was added into the solution. Then NMR spectra were recorded over time until equilibrium was reached. During the measurement, the integration of aromatic peaks of **5⁺** and **1H₄⁺** stayed the same, MeO peak of **5⁺** (4.05 ppm) decreased, and MeO peak (3.76 ppm) of **1H₄⁺** increased.

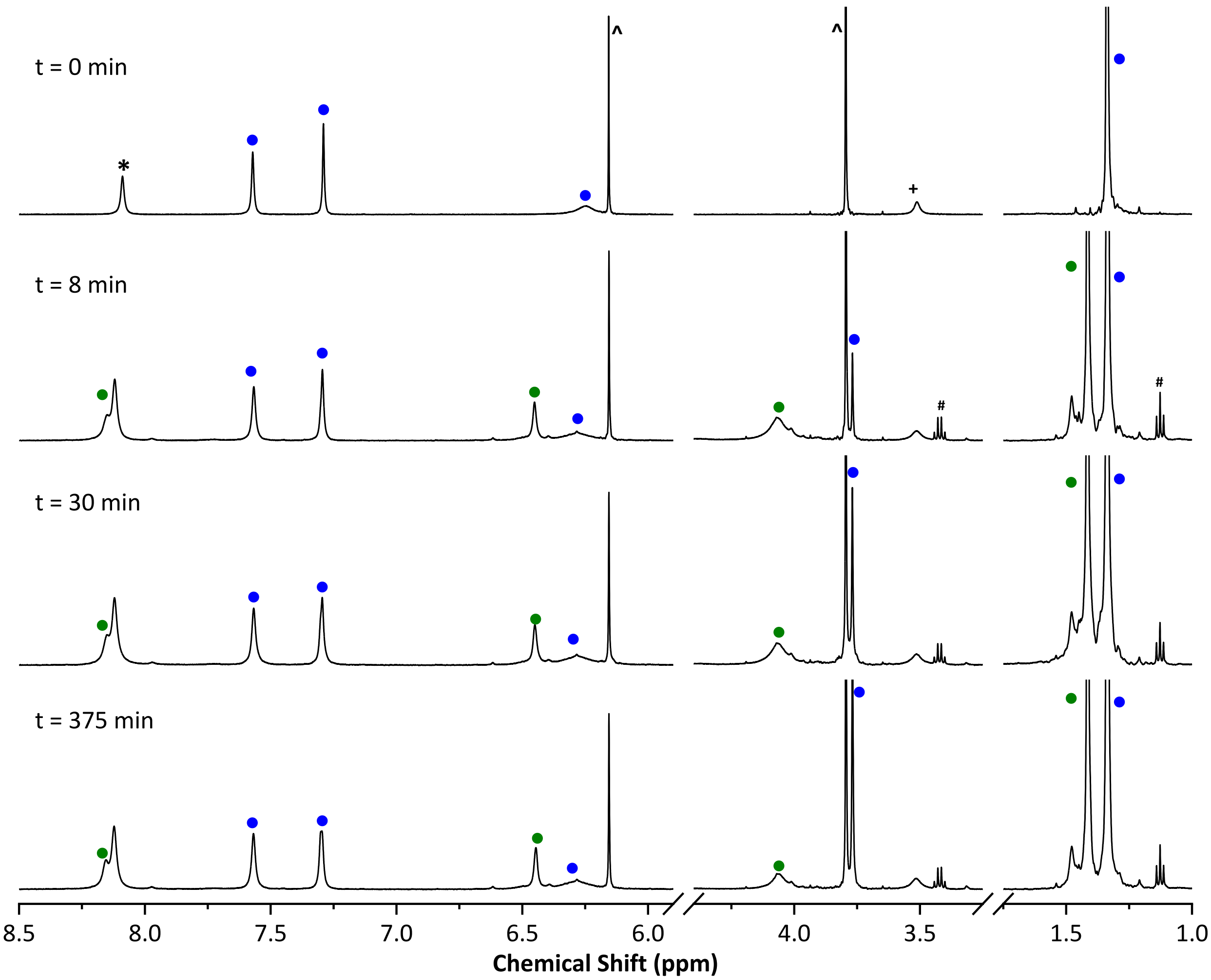

Figure S42. Selected NMR spectra of the reaction between **5⁺** and **^{D}1H₄⁺** in DMF-d_7 at room temperature. Green dot: **5⁺**; blue dot: **1H₄⁺**; ^: internal standard, 1,3,5-trimethoxybenzene; #: Et_2O; +: H_2O/HDO; *: DMF. Note: in this experiment, the amount of **5⁺** added into the reaction was quantified as 7.5 mM).

Table S9. NMR quantification of Isoergonic Ligand-exchange reaction between **5^+** and **$^{D}1H_4^+$**. Integration of the corresponding MeO peaks was used for quantification of [5^+] and [$1H_4^+$].

t (min)	[$1H_4^+$] (mM)	[5^+] (mM)	[$1H_4^+$] + [5^+] (mM)
8	0.94	3.88	4.82
12	1.16	3.63	4.80
23	1.68	3.18	4.86
30	1.94	2.93	4.87
60	2.38	2.38	4.76
75	2.50	2.23	4.73
115	2.72	2.01	4.73
180	2.80	1.92	4.73
260	2.84	1.89	4.73
310	2.85	1.87	4.72
375	2.87	1.87	4.73
800	2.87	1.86	4.73

Calculation of the reaction rate: The *k* value for the ligand-exchange reaction was calculated using the initial rates method, monitoring the change in [**5^+**] from 0 to 30 minutes (note: initial rates assumes that d[[**5^+**]/dt ~ Δ[**5^+**]/Δt):

$$r_{ini} = -\frac{d[5^+]}{dt} = \frac{d[1H_4^+]}{dt}$$

Assuming the reaction is second rate on 5^+ and $1H_4^+$:

$$r_{ini} = k \cdot [5^+] \cdot [1H_4^+]$$

Then:

$$k = \frac{-r_{ini}}{[5^+] \cdot [1H_4^+]}$$

$$= \frac{\mathrm{d}[5^+]}{dt \cdot [5^+] \cdot [1H_4^+]}$$

$$= \frac{-(2.93 - 5.00) \times 10^{-3}\, M}{1800\, s \times 5 \times 10^{-3}\, M \times 5 \times 10^{-3}\, M}$$

$$= 0.046\, M^{-1}\, s^{-1}$$

8.3. Isoergonic PCET reaction between 5^+ and 4-MeO-2,6-DTBP

General procedure: In the glovebox, 1 mL DMF-d_7 solution containing 10 mM 1,3,5-trimethoxybenzen and 10 mM 5+ was prepared. After the first NMR spectrum was recorded, the NMR tube was transferred into the glovebox, and 0.320 mmol of 4-MeO-2,6-DTBP was added into the solution. Then NMR spectrums were recorded over time until equilibrium was reached.

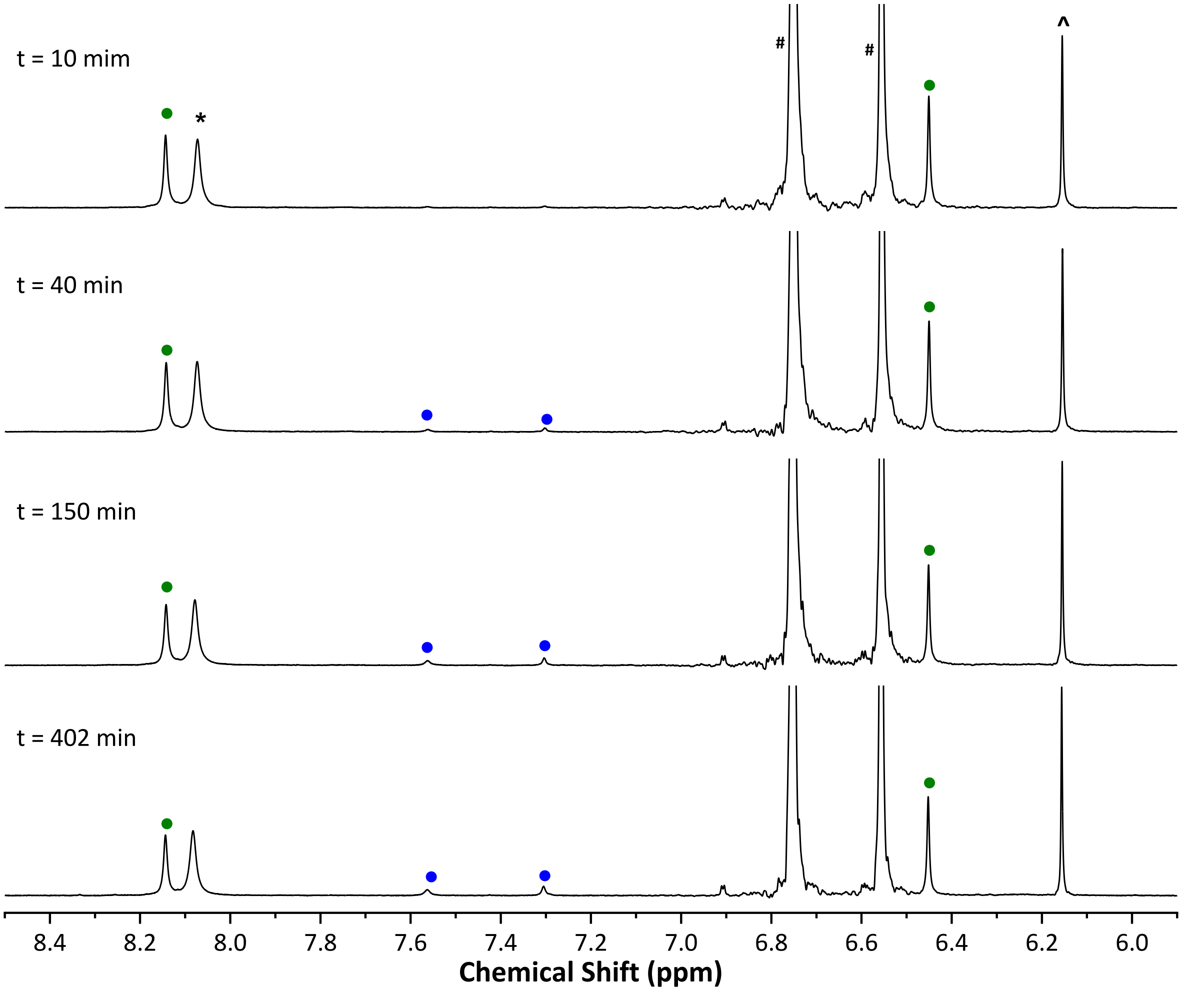

Figure S43. Selected NMR spectra of the reaction between **5^+** (10 mM) and 4-MeO-2,6-DTBP (320 mM) in DMF-d_7 at room temperature. Green dot: **5^+**; blue dot: **$1H_4^+$**; ^: internal standard, 1,3,5-trimethoxybenzene (10 mM); #: 4-MeO-2,6-DTBP; *: DMF.

Table S10. NMR quantification of reaction between **5⁺** (10 mM) and 4-MeO-2,6-DTBP (320 mM) in DMF-d_7.

t (min)	$[1H_4^+]$ (mM)	$[5^+]$ (mM)
0	0.00	10.02
10	0.15	9.91
15	0.20	9.88
20	0.24	9.75
25	0.30	9.66
40	0.32	9.57
58	0.56	9.41
73	0.73	9.29
109	0.84	9.14
150	0.85	9.14
187	0.96	9.04
247	1.05	8.83
331	1.06	8.64
402	1.13	8.58

Calculation of the reaction rate: The *k* value for the PCET reaction was calculated using the initial rates method, by monitoring the change in $[5^+]$ over 25 minutes (note: initial rates assumes that d[[$\mathbf{5^+}$]/dt ~ Δ[$\mathbf{5^+}$]/Δt):

$$r_{ini} = -\frac{d[5^+]}{dt}$$

Assuming the reaction is second rate on $\mathbf{5^+}$ and 4-MeO-2,6-DTBP:

$$r_{ini} = k \cdot [5^+] \cdot [PhOH]$$

Then:

$$k = \frac{-r_{ini}}{[5^+] \cdot [PHOH]}$$

$$= \frac{-d[5^+]}{dt \cdot [5^+] \cdot [PhOH]}$$

$$= \frac{-(10.02 - 9.66) \times 10^{-3}\, M}{1800\, s \times 10 \times 10^{-3}\, M \times 3.2 \times 10^{-1}\, M}$$

$$= 0.0000625\, M^{-1}\, s^{-1}$$

9. DFT calculations

All DFT calculations were performed with the Amsterdam Density Functional (ADF)[8, 9] and QUILD[10] programs, and were performed using the unrestricted Kohn-Sham scheme. Molecular orbitals were expanded in an uncontracted set of Slater type orbitals (STOs) of triple-ζ quality with double polarization functions (TZ2P).[11] Core electrons were not treated explicitly during the geometry optimizations (frozen core approximation[9]). An auxiliary set of s, p, d, f, and g STOs was used to fit the molecular density and to represent the Coulomb and exchange potentials accurately for each SCF cycle.
Geometries of all possible spin states were optimized with the QUILD[10] program using adapted delocalized coordinates until the maximum gradient component was less than 10^{-4} a.u. Energies, gradients and Hessians[12] (for vibrational frequencies) were calculated using S12g,[13] in all cases by including solvation effects through the COSMO[14] dielectric continuum model with appropriate parameters for the solvents.[15] For computing Gibbs free energies, all small frequencies were raised to 100 cm^{-1} in order to compensate for the breakdown of the harmonic oscillator model.[16] Scalar relativistic corrections have been included self-consistently in all calculations by using the zeroth-order regular approximation (ZORA)[17]. The geometry optimizations and frequency calculations have been performed with a Becke grid of VeryGood and Normal quality, respectively.
All computational data will be uploaded onto the IOCHEM-BD platform (www.iochem-bd.org)[18] to facilitate data exchange and dissemination, according to the FAIR principles[19] of OpenData sharing.

Table S11. Spin states ($<S^2>$) and relative energies (Kcal/mol) for the complexes **5^+**, **4**, **$4H^+$**, **3^-**, **$3H_2^+$**, **2^{2-}** , **$2H_3^+$** and **$1H_4^+$**.

Complex	$<S^2>$ (unpaired electrons)	E^{rel} (kcal·mol^{-1})
**5^+**	**0.00**	**0.0**
	2.00	18.6
**4**	**0.75**	**0.0**
	3.76	10.1
**$4H^+$**	**0.75**	**0.0**
	3.76	22.2
**3^-**	**0.00**	1.7
	2.00	**0.0**
**$3H_2^+$**	**0.00**	**0.0**
	2.00	16.5
**$3H_2^+$ (B)**	0.00	10.8
	2.00	8.5
**2^{2-}**	**0.75**	**0.0**
	3.76	63.6
**$2H_3^+$**	**0.75**	0.0
	3.76	70.7
**$1H_4^+$**	**0.00**	**0.0**
	2.00	67.8
**$1H_4^+$ (B)**	0.00	19.1

Note: For complex **3^-**, our computations suggest that the triplet state (S = 1) is slightly lower in energy than the singlet state (S = 0). However, EPR measurements indicate that complex **3** is diamagnetic (EPR silent in parallel and perpendicular mode). We carried out the single point calculations for complex **3** using two other basis sets (OBPE/TZ2P and SSB-D/TZ2P) and similar results were obtained (i.e. the triplet state had slightly lower energy than the single state, 2 kcal/mol).

Computed redox potentials

For the calculation of the redox potentials, we follow the protocol by Namazian, Lin, and Coote,[20] who computed the absolute redox potential of the ferrocene/ferrocenium couple in different solvents with several computational methods. The absolute reduction potential at S12g/TZ2P in acetonitrile is then 4.838 eV (4.876 eV directly from Fc vs. Fc^+, and a correction term of -0.0376 eV for the Gibbs free energy of an electronfrom Fermi Dirac statistics of the "electron convention"), which is reasonably close to the "experimental" value of 4.980 eV.[21] Recently we have used this protocol to correctly predict the redox potential of a $Fe^{III/IV}$ cyanide complex.[22]

Applying the same protocol to the $\mathbf{5^+/4}$, $\mathbf{4/3^-}$ and $\mathbf{3^-/2^{2-}}$ couples leads to absolute reduction potentials of 4.16, 3.80 and 3.41 eV, respectively, which should be compared to that of the Fc/Fc^+ couple (4.838 eV) to give the final redox potentials of -0.67, -1.03 and -1.42 eV, respectively.

Table S12. Summary of the computed $E_{1/2}$ for the $\mathbf{5^+/4}$, $\mathbf{4/3^-}$ and $\mathbf{3^-/2^{2-}}$ couples.

	E^{elec} (kcal·mol^{-1})	ΔGibbs (kcal·mol^{-1})	Gibbs (kcal·mol^{-1})	Gibbs (eV)	$E^{1/2}$ (eV)	$E^{1/2, a}$ (eV)
$\mathbf{5^+}$	-15194.770	507.491	-14687.279	4.20	4.16	-0.67
**4**	-15290.320	506.163	-14784.157	3.84	3.80	-1.03
$\mathbf{3^-}$	-15378.140	505.332	-14872.808	3.45	3.41	-1.42
$\mathbf{2^{2-}}$	-15457.840	505.404	-14952.436			

^{a} relative to Fc^+/Fc^0

Computed Bond Dissociation Free Energy (BDFE) and pKa

The C–H bond strength can be obtained from experiment (PT, ET) as described by Mayer, Borovik and others (see references in the main text). An extended detailed description and reference values can be found in "Comprehensive Handbook of Chemical Bond Energies", Yu-Ran Luo, CRC Press 2007 (ISBN 9780429128684). Computationally, it amounts to the homolytic cleavage of the C–H bond, typically within solution for the BDFE (bond dissociation free energy) and in the gas-phase for the BDE (bond dissociation enthalpy). Within computational chemistry, the solvent is typically taken into account through a dielectric continuum method (COSMO, PCM, SM12, SMD, ..).

$$R - C - H \rightarrow R - C^{\bullet} + H^{\bullet} \quad (1)$$

The energy required for this breaking is then the C–H bond strength, and could be indicated in (i) electronic energy, (ii) electronic energy plus zero-point vibrational energy (ΔH^{0K}), (iii) enthalpy at room temperature ($\Delta H^{298.15K}$), or the Gibbs free energy ($\Delta G^{298.15K}$); obviously, the temperature used in the determination of the bond strength can be changed in the thermodynamics part, to be able to compare directly with experimental data if these were obtained at lower or higher temperature. For each of three species (RCH, $RC^{\bullet}$ and $H^{\bullet}$) we therefore the need the electronic energy and the thermodynamic properties (resulting from vibrational frequencies, except of course for the $H^{\bullet}$).
In similar fashion, the proton affinity and basicity for the binding of a proton H^+ can be computed, with the only difference that instead of homolytic cleavage of the C–H bond, now a heterolytic cleavage occurs.

$$B - H \rightarrow B^{-} + H^{+} \quad (2)$$

For the calculation of the pK_a we follow Mayer and co-workers[23] and Cramer and co-workers,[24] although we have not added explicit solvent molecules in our calculations here. With the size and type of the complexes used here, there is no straightforward rational approach of where these should be placed and hence a long-term molecular dynamics simulation should be performed, which is clearly beyond the reach of the current study. Kelly et al. reported in 2007 the solvation free energy for the proton in acetonitrile (-260.2 $kcal \cdot mol^{-1}$), which we have used here, after correction (1.9 $kcal \cdot mol^{-1}$) for the standard state. The Gibbs free energy needed for the proton is then the total electronic bonding energy (from ADF), plus the Gibbs free energy in the gas phase (-6.275 $kcal \cdot mol^{-1}$) plus the solvation free energy (-258.3 $kcal \cdot mol^{-1}$).

The pK_a is related to the free energy change (in solution) of eq. 2, according to $pK_a = \Delta G/RT\ ln(10)$, where *R* is the universal gas constant and T the temperature. The computed Gibbs free energy for eq. 2 was 6.42 $kcal \cdot mol^{-1}$, leading to a pK_a of 4.7.

Table S13. Summary of the computed BDFE and pK_a.

**BDFE (kcal/mol)**	$\mathbf{5^{+}/4H^{+}}$	58.0
	$\mathbf{4H^{+}/3H_2^{+}}$	75.9
	$\mathbf{3H_2^{+}/2H_2^{+}}$	62.2
	$\mathbf{2H_3^{+}/1H_4^{+}}$	83.8
	$\mathbf{5^{+}/1H_4^{+}}$	70.0
	$\mathbf{^{MeO\text{-}H}5^{+}/^{MeO\text{-}H}1H_4^{+}}$	67.9
**p*K*a**	$\mathbf{4/4H^{+}}$	3.3

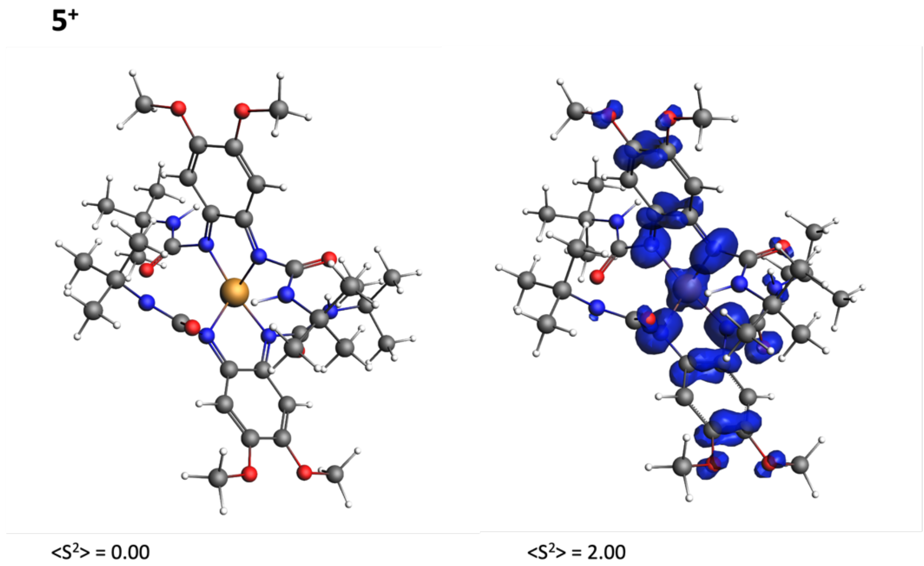

Figure S44. Spin density plots for $\mathbf{5^+}$ in different spin states.

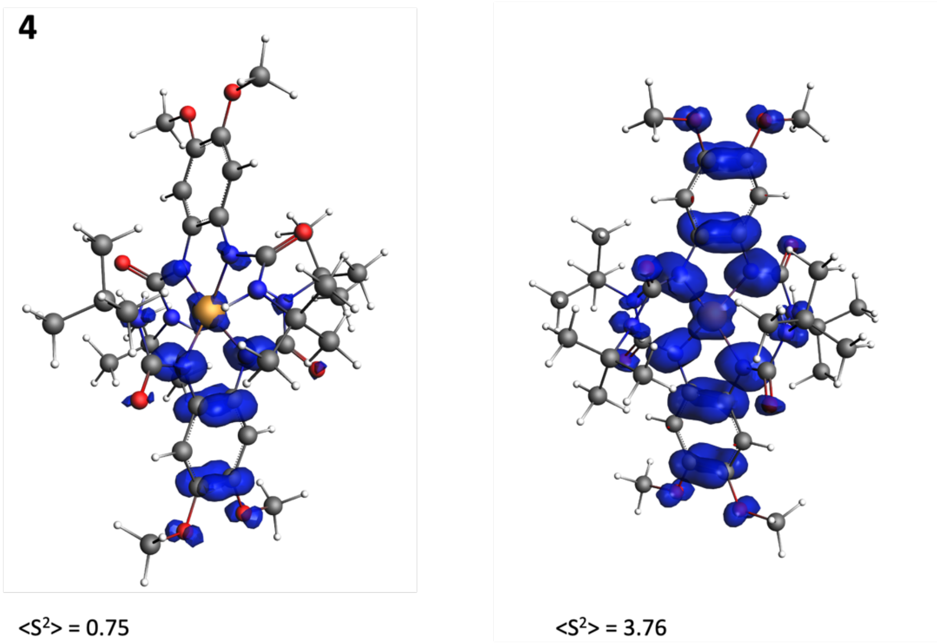

Figure S45. Spin density plots for **4** in different spin states.

4H⁺

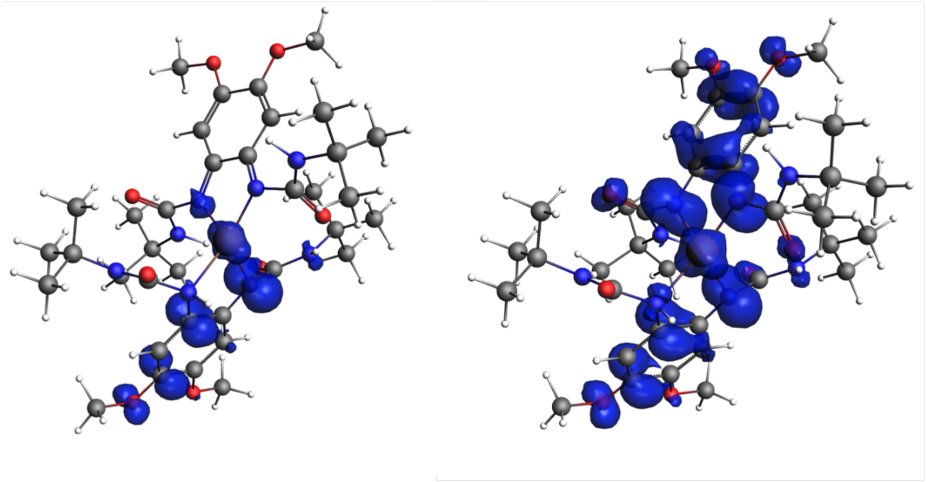

<S^2> = 0.75 <S^2> = 3.76

Figure S46. Spin density plots for **$4H^+$** in different spin states.

3⁻

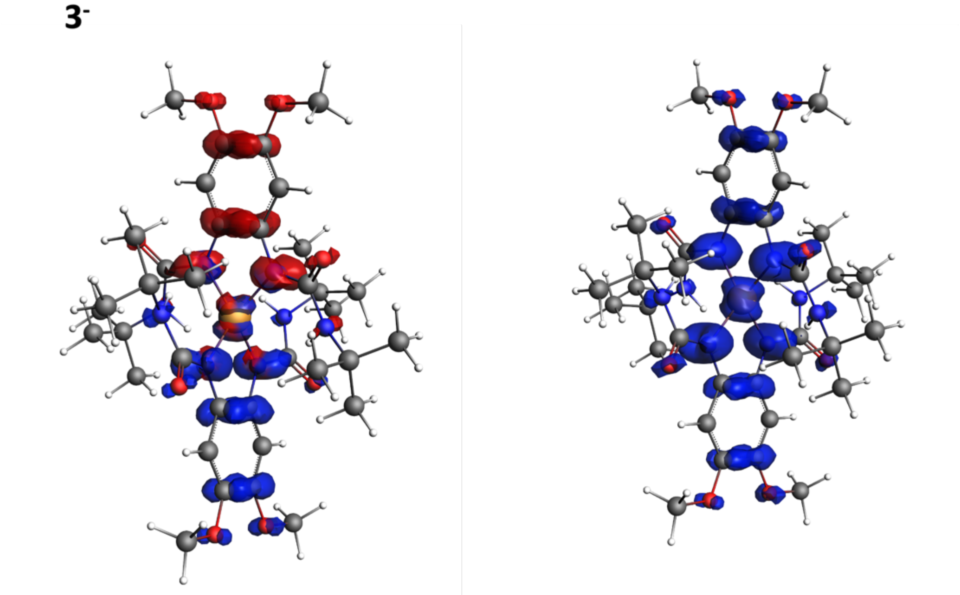

<S^2> = 0.00 <S^2> = 2.00

Figure S47. Spin density plots for **3^-** in different spin states.

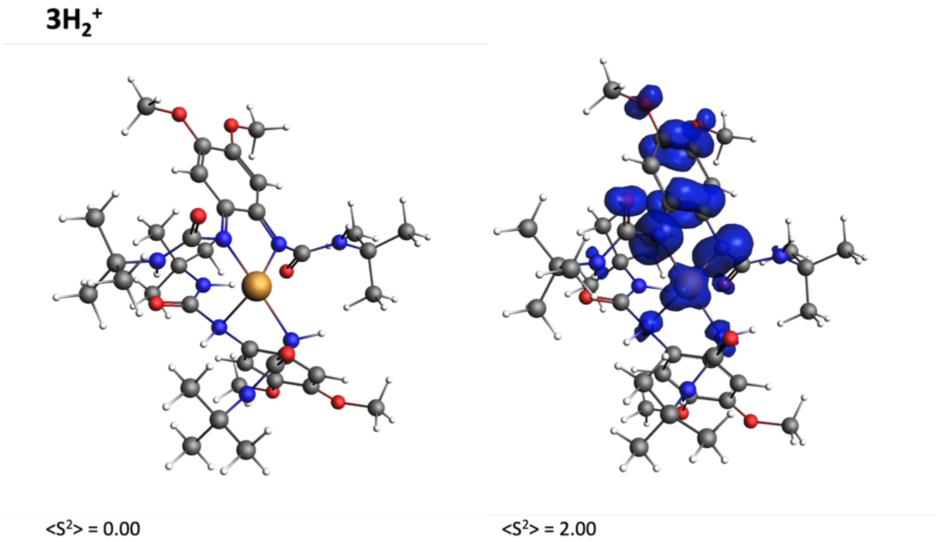

Figure S48. Spin density plots for $\mathbf{3H_2^+}$ in different spin states.

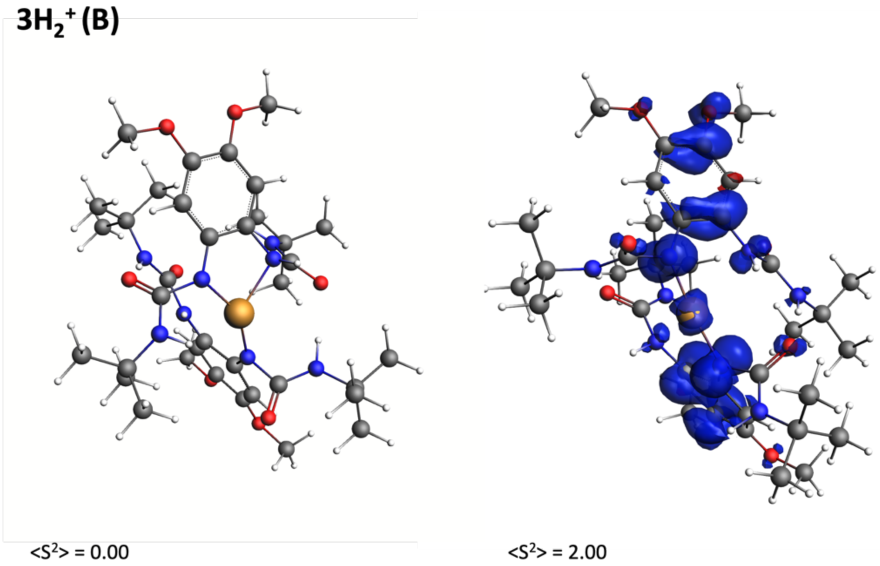

Figure S49. Spin density plots for $\mathbf{3H_2^+(B)}$ in different spin states.

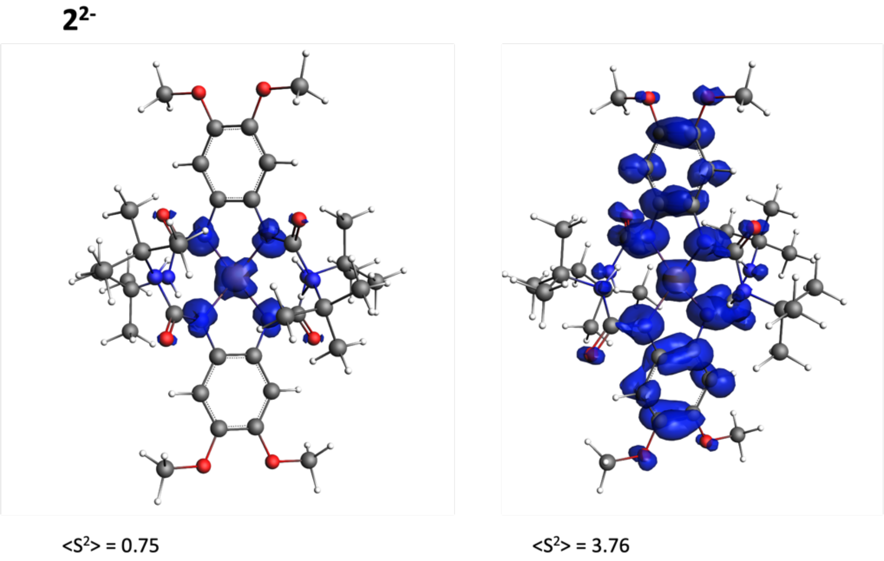

Figure S50. Spin density plots for $\mathbf{2^{2-}}$ in different spin states.

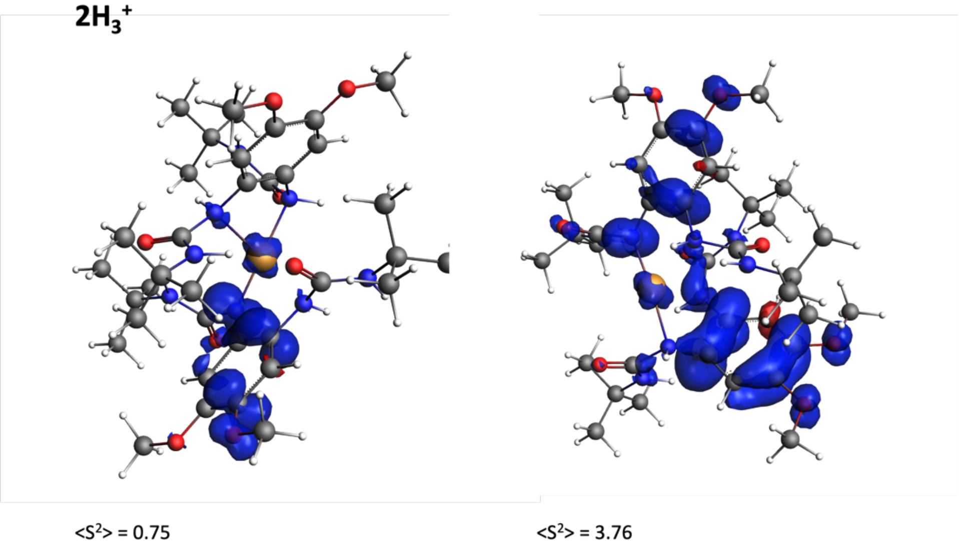

Figure S51. Spin density plots for $\mathbf{2H_3^+}$ in different spin states.

$1H_4^+$

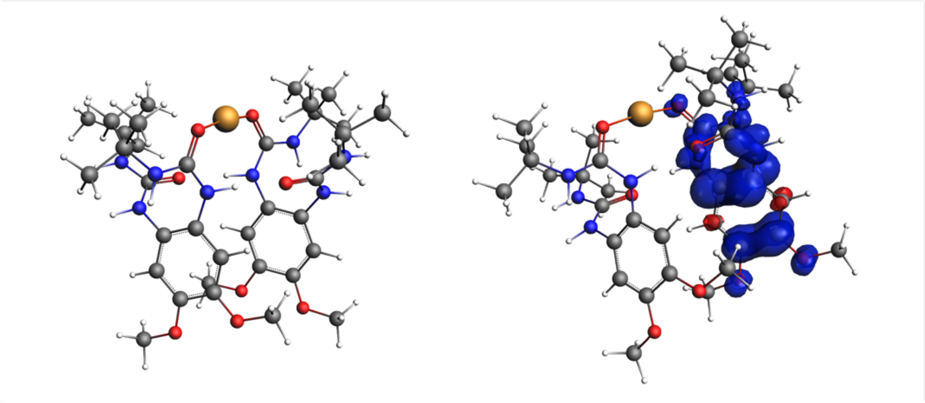

$<S^2> = 0.00$ $<S^2> = 2.00$

Figure S52. Spin density plots for **$1H_4^+$** in different spin states.

$1H_4^+$(B)

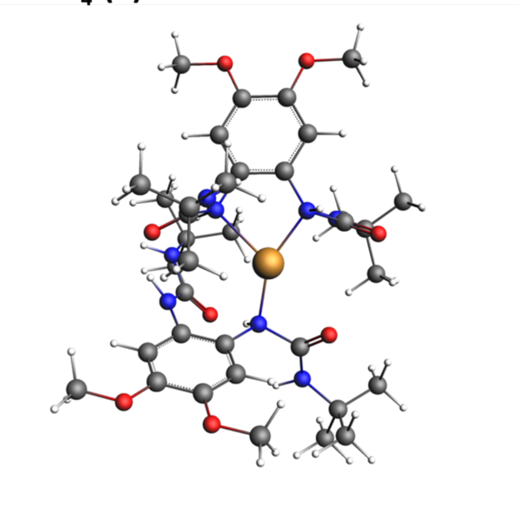

$<S^2> = 0.00$

Figure S53. Spin density plots for **$1H_4^+$** (B).

Table S14. Optimized Cartesian Coordinates for **5⁺** (<S^2> = 0.00).

Cu	0.030254	-0.776951	-0.446860
C	5.006724	-4.329163	-1.895983
H	5.637150	-3.980522	-1.069814
H	5.653289	-4.811033	-2.634467
H	4.308106	-5.081096	-1.512449
C	5.257972	-2.125819	-3.058693
H	4.741065	-1.264486	-3.489529
H	5.892515	-2.569665	-3.831926
H	5.908041	-1.778563	-2.247464
C	3.374897	-3.683170	-3.682298
H	2.657552	-4.419983	-3.306126
H	3.997005	-4.164335	-4.443584
H	2.823839	-2.865392	-4.152968
C	4.261260	-3.169053	-2.547153
C	2.621983	-1.511254	-1.620064
C	2.591947	-0.429630	0.506042
C	3.991990	-0.249962	0.594419
H	4.627946	-0.651169	-0.184140
C	4.539870	0.387034	1.675929
O	5.839936	0.595589	1.860952
C	3.685759	0.901026	2.754971
O	4.352513	1.457973	3.760348
C	2.323488	0.806660	2.663132
H	1.679568	1.215769	3.432902
C	1.731252	0.207496	1.526701
C	-0.571018	0.637578	2.112123
C	-2.379497	2.356764	2.342021
C	-3.542450	1.365553	2.242136
H	-3.733900	1.096837	1.198617
H	-4.451039	1.820656	2.648928
H	-3.334050	0.452183	2.804871
C	-2.103543	2.725825	3.800047
H	-1.863443	1.838303	4.390951
H	-2.988927	3.201468	4.233973
H	-1.267285	3.430051	3.865873
C	-2.701447	3.613062	1.536718
H	-1.878517	4.334536	1.585460
H	-3.599359	4.088338	1.941139
H	-2.881410	3.368615	0.484550
N	3.426426	-2.570271	-1.481664
H	3.466391	-2.998175	-0.565727
N	1.940702	-1.087920	-0.439241
N	0.455578	0.202368	1.232595
N	-1.164428	1.775829	1.729063
H	-0.828271	2.216193	0.873115
O	2.403384	-0.900278	-2.668207
O	-0.905806	-0.085466	3.055604
C	-4.051403	-0.727070	-1.065930
H	-4.477685	-1.657080	-0.711222
C	-4.854636	0.289254	-1.503017
O	-6.183403	0.260665	-1.533568
C	-4.265900	1.553576	-1.960957
O	-5.159277	2.477460	-2.298407
C	-2.906706	1.716497	-2.002101
H	-2.469639	2.647218	-2.337976
C	-2.045412	0.672159	-1.593471
C	-0.008157	1.929125	-1.631111
C	2.206414	2.874718	-2.295935
C	1.662213	4.133011	-2.975107
H	1.326263	3.904989	-3.992259
H	2.451812	4.889013	-3.033889
H	0.822829	4.550726	-2.414147
C	2.677468	3.189073	-0.871607
H	1.852772	3.555999	-0.255655
H	3.456952	3.957224	-0.900529
H	3.098584	2.295812	-0.399537
C	3.368803	2.311610	-3.110124
H	3.770221	1.401188	-2.652537
H	4.170591	3.053254	-3.164999
H	3.052717	2.070440	-4.130976
N	-2.235237	-2.549172	1.473714
H	-1.819810	-1.727403	1.903853
N	-1.790628	-1.405006	-0.491364
N	-0.726581	0.699877	-1.588599
N	1.168533	1.824952	-2.266521
H	1.447157	0.889903	-2.563804
O	-2.715023	-3.519692	-0.553940
O	-0.419909	2.932701	-1.036635
C	-2.459505	-3.082641	3.805880
H	-1.389562	-2.916448	3.971176
H	-2.811515	-3.817339	4.535220
H	-2.986070	-2.139300	3.988210
C	-1.957174	-4.900950	2.162262
H	-2.124192	-5.282013	1.151825
H	-2.293335	-5.658217	2.877815
H	-0.882766	-4.744206	2.305752
C	-4.227653	-3.800440	2.189600
H	-4.766974	-2.861539	2.357187
H	-4.601799	-4.542033	2.902436
H	-4.445368	-4.155434	1.178944
C	-2.724613	-3.598465	2.394209
C	-2.296946	-2.591682	0.135875
C	-2.644257	-0.567611	-1.049541
C	6.749978	0.144557	0.847503
H	6.536337	0.638441	-0.107761
H	6.692408	-0.944390	0.735921
H	7.740982	0.429474	1.199344
H	-6.615070	-1.122929	-0.029891
C	-6.846822	-0.930111	-1.084134
H	-6.556508	-1.788300	-1.699957
H	-7.911602	-0.729159	-1.199614
C	-4.685957	3.770395	-2.707081
H	-4.101459	4.234529	-1.904592
H	-5.583440	4.356998	-2.901705
H	-4.082436	3.690081	-3.617484
C	3.599436	1.983063	4.864284
H	4.341012	2.362598	5.566732
H	3.005520	1.191522	5.334414
H	2.948582	2.798942	4.529935

Table S15. Optimized Cartesian Coordinates for **5⁺** (<S^2> = 2.00).

Cu	-0.104860	0.791332	0.577391
C	5.250286	3.465508	2.403913
H	5.829325	3.145659	1.529722
H	5.954389	3.766893	3.184137
H	4.652397	4.341501	2.128281
C	5.220289	1.118402	3.274600
H	4.600633	0.283480	3.610785
H	5.914028	1.378663	4.079823
H	5.811868	0.793700	2.410903
C	3.555291	2.810222	4.128750
H	2.936099	3.673023	3.862116
H	4.236403	3.109011	4.931829
H	2.906291	2.015256	4.504001
C	4.364073	2.336575	2.920365
C	2.529633	1.023487	1.812196
C	2.400436	0.423556	-0.528228
C	3.773040	0.184612	-0.715073
H	4.465339	0.407598	0.085685
C	4.231997	-0.374910	-1.886558
O	5.502539	-0.667157	-2.134823
C	3.303665	-0.734028	-2.957024
O	3.876985	-1.267814	-4.033234
C	1.959853	-0.519487	-2.796741
H	1.258056	-0.778478	-3.581066
C	1.469893	0.093278	-1.621370
C	-0.838710	0.094862	-2.293955
C	-2.559788	-1.634587	-2.865770
C	-3.772955	-0.768953	-2.516585
H	-3.960952	-0.785485	-1.438596
H	-4.661081	-1.156790	-3.025428
H	-3.622732	0.268217	-2.827639
C	-2.304453	-1.639508	-4.373871
H	-2.136790	-0.627260	-4.749601
H	-3.170692	-2.062730	-4.892303
H	-1.429328	-2.253665	-4.613217
C	-2.781307	-3.062341	-2.373172
H	-1.917836	-3.697223	-2.599937
H	-3.659015	-3.487109	-2.868145
H	-2.948448	-3.083229	-1.291545
N	3.454686	1.992368	1.805082
H	3.518787	2.558265	0.968989
N	1.815814	0.847854	0.592568
N	0.211173	0.402031	-1.385511
N	-1.360684	-1.130981	-2.156915
H	-0.973373	-1.733243	-1.427941
O	2.239353	0.314182	2.777259
O	-1.223994	0.985533	-3.055408
C	-4.166203	0.444685	1.215667
H	-4.632928	1.412996	1.097990
C	-4.929110	-0.687929	1.398242
O	-6.261337	-0.707159	1.463059
C	-4.296511	-1.992224	1.485236
O	-5.143005	-3.022201	1.587817
C	-2.926458	-2.096711	1.452747
H	-2.446297	-3.062165	1.539340
C	-2.123267	-0.945395	1.329742
C	-0.053628	-2.107010	1.077239
C	2.046642	-3.331713	1.659464
C	1.377933	-4.676937	1.953345
H	0.953928	-4.681625	2.963045
H	2.119304	-5.479553	1.886342
H	0.578792	-4.882454	1.236582
C	2.645760	-3.323185	0.249428
H	1.871606	-3.458365	-0.509948
H	3.373171	-4.135600	0.152987
H	3.162999	-2.378578	0.057810
C	3.143453	-3.064245	2.687101
H	3.634788	-2.103093	2.501513
H	3.899607	-3.852257	2.630623
H	2.735813	-3.052267	3.703967
N	-1.850900	3.134824	-0.720767
H	-1.405300	2.477957	-1.354145
N	-1.936525	1.307748	0.679446
N	-0.782900	-0.920256	1.388802
N	1.059224	-2.245613	1.810163
H	1.319826	-1.445440	2.385121
O	-3.133648	3.226089	1.180421
O	-0.418133	-2.870181	0.173257
C	-1.216030	4.591996	-2.512734
H	-0.155664	4.453037	-2.273117
H	-1.338034	5.580303	-2.964464
H	-1.506489	3.837042	-3.251651
C	-1.628112	5.534435	-0.232995
H	-2.223138	5.476282	0.681534
H	-1.744982	6.536053	-0.658631
H	-0.573442	5.389647	0.024661
C	-3.553820	4.673523	-1.617260
H	-3.865908	3.921223	-2.349862
H	-3.708853	5.664960	-2.055101
H	-4.187817	4.583129	-0.731326
C	-2.077212	4.490572	-1.255796
C	-2.380058	2.631326	0.409953
C	-2.768308	0.343174	1.105910
C	6.485239	-0.368644	-1.130999
H	6.286149	-0.942855	-0.218633
H	6.499052	0.705101	-0.913792
H	7.438353	-0.675239	-1.560240
H	-6.791834	1.019553	0.413457
C	-6.967884	0.538256	1.382535
H	-6.667178	1.206835	2.196739
H	-8.022064	0.279084	1.480952
C	-4.598950	-4.348274	1.585444
H	-4.028532	-4.529435	0.666495
H	-5.460049	-5.015669	1.624144
H	-3.961579	-4.510602	2.461975
C	3.030856	-1.661476	-5.123405
H	3.702561	-2.064923	-5.880656
H	2.491388	-0.795585	-5.523588
H	2.323752	-2.434732	-4.801088

Table S16. Optimized Cartesian Coordinates for **4** (<S^2> = 0.75).

Cu	0.084428	-0.193517	-0.177267
H	-2.282418	4.099563	4.060703
C	2.488762	2.858014	-2.992724
H	2.969837	1.994439	-2.519143
H	3.271350	3.528278	-3.363220
H	1.902482	2.506074	-3.846233
C	2.431799	4.033842	-0.783206
H	1.806247	4.534060	-0.034927
H	3.206980	4.735275	-1.104391
H	2.927034	3.178744	-0.307185
C	0.927610	4.806465	-2.627048
H	0.310370	4.507861	-3.477460
H	1.690859	5.508900	-2.977280
H	0.292383	5.320975	-1.897978
C	1.598903	3.592309	-1.985968
C	-0.355424	1.996545	-2.153157
C	-2.261695	0.713616	-1.404431
C	-3.248947	1.486054	-2.053631
H	-2.935485	2.340404	-2.639000
C	-4.572986	1.136522	-1.969048
H	-3.061979	1.673052	4.363468
C	-4.976964	-0.038104	-1.210511
H	-2.511068	0.430887	3.213020
C	-4.039310	-0.798287	-0.557626
H	-4.334031	-1.647087	0.044901
C	-2.673186	-0.448058	-0.605270
C	-1.876582	-1.979442	1.091041
C	-0.404983	-3.565850	2.375378
C	-1.453722	-4.535209	2.917460
H	-2.366764	-4.003763	3.195696
H	-1.063118	-5.049446	3.801911
H	-1.704347	-5.288269	2.162479
C	-0.051335	-2.496384	3.415718
H	0.694801	-1.801220	3.013476
H	0.361731	-2.966881	4.314024
H	-0.935604	-1.920822	3.703768
C	0.856399	-4.331138	1.980341
H	0.635589	-5.099174	1.231910
H	1.281093	-4.824041	2.859475
H	1.618486	-3.657198	1.570175
H	-0.584162	3.573206	4.061969
C	-3.074148	2.713160	1.818574
H	-3.398352	1.794493	1.314549
H	-3.915068	3.085001	2.410781
H	-2.834016	3.463568	1.056350
H	0.077684	-1.854928	-1.919790
N	-0.796486	1.987637	1.834704
H	-1.101664	1.709075	0.913023
O	2.891337	-3.056301	-1.172004
O	0.905662	1.677388	3.337758
N	1.766523	-1.130151	-0.441136
N	1.046099	0.778846	1.194583
N	0.764967	-2.595840	-1.877177
C	0.584694	-5.048594	-1.899560
H	-0.150849	-5.032168	-1.088201
H	0.373021	-5.914226	-2.535393
H	1.580842	-5.168332	-1.466896
C	-0.934067	-3.580591	-3.232082
H	-1.027135	-2.666827	-3.831389
H	-1.221407	-4.427322	-3.861571
H	-1.639927	-3.522792	-2.394768
C	1.468550	-3.809007	-3.908326
H	2.498484	-3.915821	-3.558271
N	0.584908	2.667772	-1.451729
H	0.771097	2.309215	-0.523467
N	-0.951244	0.961059	-1.390348
N	-1.679087	-1.056063	0.040081
N	-0.890032	-2.912170	1.142759
H	-0.193138	-2.816925	0.415050
H	1.230748	-4.658615	-4.557106
H	1.393253	-2.890611	-4.500737
O	-0.644034	2.197103	-3.335204
O	-2.791910	-1.874579	1.908589
C	0.497042	-3.763750	-2.726501
C	1.904305	-2.312403	-1.178462
C	2.775801	-0.564557	0.272767
C	4.141706	-0.903533	0.204211
H	4.428450	-1.727442	-0.431855
C	5.090907	-0.190271	0.904671
C	-1.461227	3.739588	3.431962
C	4.697897	0.915626	1.733703
H	-1.220760	4.516257	2.697593
C	3.364232	1.242624	1.848563
H	3.049057	2.055360	2.488394
C	2.377550	0.521662	1.152240
C	0.423420	1.512174	2.212014
C	-1.872548	2.447556	2.725453
C	-2.224474	1.350634	3.735482
H	-1.370891	1.131463	4.382892
O	5.704425	1.563949	2.352484
C	5.373795	2.694251	3.162797
H	6.324346	3.068557	3.545156
H	4.881461	3.473267	2.567617
H	4.727175	2.403672	3.999662
O	6.415129	-0.440007	0.868041
C	6.869148	-1.534783	0.068694
H	6.625371	-1.377610	-0.989103
H	6.432074	-2.480259	0.412418
H	7.952140	-1.560783	0.197318
O	-5.580086	1.799824	-2.549127
C	-5.265893	2.976634	-3.303217
H	-4.615559	2.732175	-4.151122
H	-4.784816	3.728829	-2.666920
H	-6.222403	3.353690	-3.665768
O	-6.294664	-0.270924	-1.203854
C	-6.775877	-1.399779	-0.464180
H	-6.532410	-1.297337	0.599885
H	-6.352688	-2.331721	-0.856569
H	-7.857656	-1.396038	-0.599613

Table S17. Optimized Cartesian Coordinates for **4** (<S^2> = 3.76).

Cu	-0.011465	-0.023784	-0.276359
H	1.383580	-4.202077	4.408072
C	-1.220672	-3.153311	-3.816822
H	-1.772494	-2.209878	-3.739857
H	-1.820136	-3.856706	-4.404169
H	-0.284234	-2.967559	-4.349241
C	-2.276083	-3.937860	-1.687036
H	-2.106266	-4.336057	-0.679749
H	-2.900043	-4.650441	-2.233636
H	-2.833550	-2.997534	-1.597842
C	-0.197820	-5.057234	-2.517265
H	0.762541	-4.922814	-3.020481
H	-0.791648	-5.783778	-3.081704
H	-0.014346	-5.463387	-1.516561
C	-0.952333	-3.729576	-2.423782
C	1.026514	-2.243695	-1.884728
C	2.614482	-0.682457	-0.875029
C	3.797740	-1.280272	-1.343138
H	3.719323	-2.201937	-1.900538
C	5.029560	-0.719154	-1.080098
H	2.120038	-1.763254	4.681086
C	5.116506	0.510101	-0.337590
H	1.820786	-0.606102	3.364980
C	3.967486	1.110828	0.132131
H	4.020528	2.030667	0.696051
C	2.703362	0.554999	-0.121545
C	1.393665	1.952824	1.373683
C	-0.460297	3.086250	2.670515
C	0.273500	4.363758	3.078731
H	1.308307	4.148534	3.354087
H	-0.230492	4.823174	3.935478
H	0.274909	5.083577	2.252892
C	-0.461965	2.061585	3.809931
H	-0.988114	1.149113	3.505156
H	-0.967135	2.475001	4.689123
H	0.559449	1.792325	4.092129
C	-1.898642	3.422587	2.275066
H	-1.919838	4.129094	1.437214
H	-2.422587	3.877876	3.120083
H	-2.450991	2.521123	1.982192
H	-0.278628	-3.673568	4.063744
C	2.595542	-2.985184	2.263017
H	3.021283	-2.109957	1.757616
H	3.305155	-3.309819	3.029130
H	2.491642	-3.793251	1.529540
H	0.503348	2.062541	-1.342349
N	0.367022	-2.253276	1.800396
H	0.839281	-2.069653	0.923467
O	-2.478389	2.759958	-2.088075
O	-1.585187	-1.848445	2.928688
N	-1.567197	1.060852	-0.756172
N	-1.307436	-1.102879	0.733347
N	-0.213222	2.535922	-1.882301
C	-0.264986	4.986318	-1.636351
H	0.098134	4.895892	-0.606281
H	0.106786	5.928424	-2.052687
H	-1.357546	5.023099	-1.618229
C	1.756816	3.758990	-2.453757
H	2.133122	2.906186	-3.030299
H	2.166279	4.674910	-2.889021
H	2.133221	3.675380	-1.426923
C	-0.258071	3.921430	-3.924599
H	-1.349105	3.948163	-3.969003
N	-0.196975	-2.765471	-1.614610
H	-0.699156	-2.362819	-0.831271
N	1.362642	-1.207016	-0.990727
N	1.515480	1.111202	0.249928
N	0.150210	2.499206	1.468164
H	-0.520312	2.175358	0.783179
H	0.133414	4.840004	-4.374268
H	0.095549	3.069663	-4.515716
O	1.746207	-2.619261	-2.815547
O	2.294411	2.154156	2.192250
C	0.228410	3.805684	-2.480076
C	-1.498620	2.171268	-1.618119
C	-2.733141	0.492541	-0.328455
C	-4.034165	0.942298	-0.616701
H	-4.139666	1.801537	-1.260497
C	-5.142187	0.323580	-0.075341
C	0.695044	-3.887427	3.616820
C	-4.985762	-0.813432	0.791361
H	0.580395	-4.715226	2.908581
C	-3.720073	-1.270834	1.089789
H	-3.582727	-2.125576	1.736801
C	-2.580140	-0.659416	0.544844
C	-0.896726	-1.761161	1.908690
C	1.249304	-2.651986	2.906656
C	1.422092	-1.484937	3.884466
H	0.466774	-1.212839	4.341154
O	-6.126271	-1.359242	1.245564
C	-6.030016	-2.490631	2.116811
H	-7.058376	-2.756935	2.363313
H	-5.539689	-3.331951	1.612521
H	-5.482371	-2.234582	3.031519
O	-6.409944	0.711715	-0.291514
C	-6.634931	1.843104	-1.138903
H	-6.256906	1.655635	-2.150826
H	-6.160996	2.741298	-0.725394
H	-7.717157	1.975220	-1.166063
O	6.205518	-1.242493	-1.466818
C	6.186216	-2.465338	-2.209521
H	5.641358	-2.341515	-3.152949
H	5.733518	-3.272826	-1.621696
H	7.231862	-2.698636	-2.413449
O	6.358512	0.993161	-0.163524
C	6.509530	2.213442	0.568623
H	6.130891	2.105385	1.592042
H	5.991095	3.038483	0.065515
H	7.582143	2.409497	0.589626

Table S18. Optimized Cartesian Coordinates for **4H$^+$** (<S^2> = 0.75).

Cu	0.164679	-0.777063	-0.571784
C	5.516465	-4.171877	-0.754983
H	5.984058	-3.651981	0.089419
H	6.304607	-4.683607	-1.313895
H	4.828286	-4.929475	-0.363916
C	5.772000	-2.133844	-2.184733
H	5.264365	-1.393794	-2.808689
H	6.551884	-2.613374	-2.784147
H	6.259624	-1.616754	-1.349996
C	4.133546	-3.939066	-2.830917
H	3.427376	-4.686403	-2.454392
H	4.900783	-4.453473	-3.418138
H	3.598072	-3.250289	-3.488817
C	4.791238	-3.191500	-1.671046
C	2.912562	-1.582374	-1.235113
C	2.541601	-0.282099	0.735533
C	3.910010	-0.014625	0.982094
H	4.657895	-0.445306	0.329414
C	4.287804	0.760530	2.044154
O	5.541690	1.066310	2.359229
C	3.278884	1.328344	2.949236
O	3.784403	2.006934	3.972824
C	1.946978	1.153309	2.694744
H	1.194465	1.591409	3.338875
C	1.523584	0.402749	1.570007
C	-0.793408	0.858788	1.882980
C	-2.590853	2.603660	1.714372
C	-3.798120	1.668628	1.818665
H	-3.997006	1.188451	0.854495
H	-4.688873	2.237614	2.103274
H	-3.633580	0.898886	2.575350
C	-2.285137	3.249779	3.066726
H	-2.078998	2.494623	3.828794
H	-3.144710	3.844948	3.391389
H	-1.418357	3.914614	2.986063
C	-2.870656	3.688978	0.677895
H	-2.013712	4.362187	0.569428
H	-3.735941	4.279195	0.992209
H	-3.086129	3.250098	-0.300957
N	3.758343	-2.540277	-0.835541
H	3.710778	-2.816793	0.136404
N	2.057220	-1.046744	-0.225414
N	0.288285	0.289676	1.145709
N	-1.413833	1.852437	1.226266
H	-1.060838	2.110902	0.304340
O	2.791046	-1.153066	-2.383070
O	-1.112624	0.392543	2.976810
C	-3.971006	-1.116042	-1.150784
H	-4.353458	-2.023144	-0.698010
C	-4.852221	-0.134034	-1.598378
O	-6.182713	-0.199878	-1.477701
C	-4.330097	1.043084	-2.227122
O	-5.253574	1.937959	-2.622259
C	-2.967833	1.201667	-2.362937
H	-2.566696	2.097726	-2.817101
C	-2.063749	0.239013	-1.861075
C	-0.078418	1.597676	-2.051897
C	2.118497	2.603648	-2.704846
C	1.541400	3.815988	-3.437338
H	1.226345	3.537892	-4.448790
H	2.308959	4.592604	-3.517181
H	0.681857	4.227101	-2.903934
C	2.548792	2.981264	-1.282508
H	1.694234	3.320102	-0.690399
H	3.286630	3.789398	-1.317250
H	3.007844	2.124248	-0.778929
C	3.317192	2.061432	-3.479828
H	3.740564	1.179335	-2.988238
H	4.092965	2.830094	-3.539829
H	3.029796	1.783597	-4.499969
N	-2.599210	-2.239976	1.354799
H	-2.769401	-1.243922	1.325532
N	-1.665591	-1.906467	-0.783844
N	-0.709113	0.336132	-1.893239
N	1.123595	1.513065	-2.655072
H	1.479940	0.573750	-2.812837
O	-1.534682	-3.945045	0.250032
O	-0.567353	2.636265	-1.589625
C	-3.708245	-1.916399	3.462333
H	-3.111182	-1.024820	3.677466
H	-3.992102	-2.374218	4.413843
H	-4.627516	-1.617273	2.944880
C	-1.611095	-3.277187	3.356376
H	-1.023700	-3.991774	2.773711
H	-1.839233	-3.728731	4.327321
H	-1.011690	-2.375682	3.520335
C	-3.764589	-4.158952	2.368711
H	-4.689992	-3.886413	1.848922
H	-4.033287	-4.624331	3.322162
H	-3.227022	-4.892976	1.764757
C	-2.910595	-2.918818	2.633136
C	-1.946294	-2.791750	0.322025
C	-2.605635	-0.935515	-1.261884
C	6.593626	0.574946	1.515456
H	6.479116	0.961118	0.495884
H	6.602176	-0.521266	1.510831
H	7.517194	0.951481	1.953977
C	2.873658	2.579615	4.924094
H	2.257655	1.798148	5.382520
H	3.502730	3.051444	5.678476
H	2.238094	3.331104	4.442130
C	-4.796475	3.162336	-3.204706
H	-4.167962	3.720721	-2.499735
H	-5.698711	3.734423	-3.423617
H	-4.241057	2.975226	-4.131171
C	-6.753593	-1.325461	-0.797733
H	-6.381175	-1.385457	0.232546
H	-6.537848	-2.256832	-1.332945
H	-7.828325	-1.144259	-0.789111
H	-1.353342	-2.514604	-1.544955

Table S19. Optimized Cartesian Coordinates for $\mathbf{4H^+}$ ($\langle S^2 \rangle$ = 3.76).

Cu	0.140197	-1.022892	-0.452650
C	6.062761	-3.390528	-0.991311
H	6.534057	-2.571578	-0.435209
H	6.826296	-3.851183	-1.624082
H	5.714487	-4.144940	-0.276574
C	5.430807	-1.811728	-2.822171
H	4.626084	-1.422051	-3.449518
H	6.196798	-2.245598	-3.472278
H	5.883135	-0.978656	-2.272463
C	4.271053	-4.040143	-2.618116
H	3.905814	-4.802079	-1.921420
H	5.010171	-4.503508	-3.279771
H	3.432618	-3.690390	-3.225756
C	4.913488	-2.879390	-1.856524
C	2.790372	-1.660999	-1.238956
C	2.478220	-0.363801	0.828530
C	3.808487	-0.004019	1.063186
H	4.580038	-0.357092	0.392606
C	4.156381	0.840015	2.111601
O	5.408761	1.234878	2.373445
C	3.138836	1.372978	2.969742
O	3.569264	2.170653	3.960394
C	1.815591	1.070822	2.717531
H	1.034280	1.485939	3.343467
C	1.449278	0.216069	1.659425
C	-0.878557	0.602215	1.980201
C	-2.515256	2.495671	1.767298
C	-3.795604	1.672573	1.920825
H	-4.053483	1.183297	0.975357
H	-4.627581	2.326329	2.200006
H	-3.687915	0.913225	2.698215
C	-2.133765	3.159701	3.092364
H	-1.983587	2.416371	3.878812
H	-2.932434	3.839537	3.405661
H	-1.213639	3.742992	2.976676
C	-2.715383	3.566090	0.698283
H	-1.807661	4.162784	0.559873
H	-3.525615	4.235275	1.000304
H	-2.976354	3.116445	-0.263735
N	3.943233	-2.278082	-0.917839
H	4.087507	-2.482741	0.061691
N	2.017761	-1.155026	-0.185601
N	0.179232	-0.079182	1.321752
N	-1.410819	1.628231	1.294635
H	-0.975381	1.891133	0.411571
O	2.353103	-1.547215	-2.395242
O	-1.254239	0.204818	3.085210
C	-3.990264	-1.014870	-1.084987
H	-4.436988	-1.889099	-0.627393
C	-4.803946	0.028895	-1.534607
O	-6.125529	0.057054	-1.401554
C	-4.203580	1.168653	-2.175874
O	-5.066147	2.114944	-2.571125
C	-2.836778	1.231610	-2.314597
H	-2.374864	2.093399	-2.776863
C	-1.997628	0.208041	-1.812435
C	0.060725	1.430865	-1.989800
C	2.288711	2.318913	-2.689594
C	1.771813	3.462424	-3.565069
H	1.513125	3.094465	-4.563586
H	2.547842	4.227720	-3.668732
H	0.886487	3.924565	-3.121093
C	2.677674	2.835313	-1.299485
H	1.823662	3.293124	-0.794918
H	3.469099	3.585881	-1.394533
H	3.054492	2.019567	-0.674832
C	3.499986	1.666100	-3.349212
H	3.896263	0.853470	-2.732220
H	4.291238	2.409190	-3.482415
H	3.243288	1.257729	-4.333037
N	-2.761540	-2.297282	1.391852
H	-2.872336	-1.292410	1.390630
N	-1.745131	-1.957257	-0.719529
N	-0.650667	0.210397	-1.843382
N	1.256601	1.269966	-2.579152
H	1.540883	0.308233	-2.761068
O	-1.756946	-4.024832	0.266094
O	-0.368332	2.491448	-1.517750
C	-3.927782	-1.975795	3.470160
H	-3.302612	-1.116087	3.729516
H	-4.265071	-2.443820	4.398823
H	-4.814871	-1.625351	2.929110
C	-1.883958	-3.422110	3.396572
H	-1.310456	-4.149166	2.815370
H	-2.160709	-3.884496	4.349486
H	-1.251728	-2.551719	3.598650
C	-4.040618	-4.187619	2.321185
H	-4.935591	-3.863791	1.778038
H	-4.361246	-4.660559	3.254464
H	-3.515477	-4.931950	1.719204
C	-3.144570	-2.992640	2.644877
C	-2.109846	-2.857300	0.366736
C	-2.622217	-0.929052	-1.204132
C	6.467033	0.754744	1.535856
H	6.325979	1.088555	0.500580
H	6.532560	-0.338959	1.576919
H	7.379320	1.193693	1.939830
C	2.592002	2.726169	4.846725
H	2.036625	1.933666	5.362297
H	3.156202	3.313159	5.571953
H	1.897946	3.379578	4.303896
C	-4.535831	3.304688	-3.168975
H	-3.868926	3.826321	-2.471790
H	-5.402278	3.929559	-3.386474
H	-4.000702	3.071498	-4.096444
C	-6.779393	-1.022008	-0.712719
H	-6.406117	-1.101385	0.315254
H	-6.634287	-1.966290	-1.247402
H	-7.836073	-0.757487	-0.701863
H	-1.482525	-2.581300	-1.488255

Table S20. Optimized Cartesian Coordinates for **3**$^-$ (<S^2> = 0.00).

Cu	-0.025162	0.013698	-0.250711
C	-2.006649	2.639440	-3.295104
H	-2.097154	1.871274	-4.071749
H	-2.593976	3.510072	-3.602137
H	-2.440744	2.244968	-2.367176
C	0.037658	3.576411	-4.392747
H	1.086982	3.851121	-4.260812
H	-0.521101	4.462248	-4.714621
H	-0.027398	2.819998	-5.182720
C	-0.451824	4.082116	-1.972944
H	-0.869915	3.685968	-1.040776
H	-1.008625	4.983896	-2.250088
H	0.589336	4.362691	-1.788517
C	-0.544036	3.034004	-3.088108
C	1.411409	1.736246	-2.147783
C	2.714428	0.248479	-0.718512
C	3.998638	0.591599	-1.177180
H	4.069404	1.293327	-1.995466
C	5.136939	0.087136	-0.579543
O	6.405450	0.394391	-0.960879
C	5.020707	-0.805591	0.525981
O	6.193246	-1.234446	1.066655
C	3.772040	-1.169192	0.985040
H	3.666198	-1.829380	1.833679
C	2.594214	-0.672201	0.391096
C	0.990941	-1.719232	1.890959
C	-1.134316	-2.568214	2.969698
C	-0.601418	-3.819331	3.670296
H	-0.545393	-4.655389	2.964201
H	-1.269140	-4.103540	4.491124
H	0.397035	-3.638566	4.075187
C	-1.222413	-1.389835	3.947031
H	-0.235852	-1.152096	4.354960
H	-1.891873	-1.636446	4.778768
H	-1.607728	-0.498695	3.439006
C	-2.519982	-2.856412	2.391653
H	-2.938615	-1.968406	1.904168
H	-3.202854	-3.159593	3.191113
H	-2.474813	-3.664419	1.652170
N	0.143540	1.793500	-2.687722
H	-0.490182	1.121250	-2.267970
N	1.531146	0.700537	-1.235168
N	1.321867	-0.998016	0.750330
N	-0.284589	-2.215032	1.824276
H	-0.792523	-1.943667	0.991739
O	2.301682	2.544152	-2.458164
O	1.727298	-1.901004	2.874402
C	-4.071233	-0.826303	-0.795114
H	-4.164550	-1.634765	-1.505444
C	-5.183670	-0.320210	-0.152073
O	-6.454304	-0.758590	-0.357949
C	-5.032716	0.715414	0.815902
O	-6.179726	1.141917	1.410461
C	-3.775826	1.205403	1.104577
H	-3.641565	1.969630	1.856513
C	-2.622962	0.700109	0.470231
C	-0.960403	1.974744	1.708307
C	1.227243	2.944689	2.532686
C	0.732532	4.241018	3.175445
H	0.601186	5.017352	2.413374
H	1.461836	4.597865	3.911030
H	-0.224557	4.080765	3.677317
C	1.417112	1.848747	3.588071
H	0.472182	1.632526	4.094799
H	2.148486	2.167663	4.339145
H	1.774448	0.923799	3.121880
C	2.554353	3.200738	1.817698
H	2.941675	2.281889	1.361346
H	3.299434	3.566996	2.530445
H	2.434111	3.951705	1.028137
N	-0.285776	-2.025175	-2.480376
H	0.342798	-1.281426	-2.194327
N	-1.614921	-0.816220	-1.061604
N	-1.345070	1.122783	0.681077
N	0.292267	2.492041	1.492132
H	0.747002	2.124723	0.665256
O	-2.379444	-2.828451	-1.984672
O	-1.631012	2.250307	2.716456
C	1.849737	-2.867081	-3.156005
H	1.833907	-2.214520	-4.036501
H	2.449096	-3.751127	-3.393691
H	2.345514	-2.329910	-2.337697
C	-0.226635	-4.036674	-3.913959
H	-1.246170	-4.328076	-3.651307
H	0.348558	-4.938604	-4.150534
H	-0.264762	-3.405835	-4.809010
C	0.490852	-4.154179	-1.500094
H	0.970360	-3.606656	-0.681093
H	1.063579	-5.067842	-1.693096
H	-0.514969	-4.438856	-1.177326
C	0.432480	-3.282441	-2.760165
C	-1.510794	-1.951897	-1.848349
C	-2.777738	-0.356326	-0.506342
C	6.565561	1.309812	-2.041970
H	6.117687	2.284344	-1.807492
H	6.121366	0.918116	-2.966235
H	7.642913	1.425044	-2.174349
C	6.119059	-2.090632	2.204218
H	5.613547	-3.034661	1.962499
H	7.153027	-2.293085	2.489836
H	5.596796	-1.601608	3.036974
C	-6.067033	2.143865	2.418517
H	-5.445726	1.798598	3.255266
H	-7.084639	2.325461	2.769552
H	-5.647246	3.073995	2.013695
C	-6.638463	-1.841674	-1.266625
H	-6.088213	-2.733078	-0.937974
H	-6.320118	-1.571321	-2.281697
H	-7.709781	-2.050935	-1.263605

Table S21. Optimized Cartesian Coordinates for **3**$^-$ ($\langle S^2 \rangle = 2.00$).

Cu	0.008298	0.065170	-0.243461
C	2.839969	-2.416984	-2.695814
H	2.828829	-1.817283	-3.613209
H	3.634362	-3.164423	-2.783532
H	3.086015	-1.757038	-1.854510
C	1.183280	-4.023641	-3.659575
H	0.215492	-4.512263	-3.524729
H	1.957952	-4.793247	-3.749371
H	1.156271	-3.449506	-4.592434
C	1.534215	-3.900308	-1.167085
H	1.760738	-3.236039	-0.326144
H	2.305044	-4.677104	-1.216427
H	0.571511	-4.382273	-0.972470
C	1.492381	-3.105808	-2.477782
C	-0.796025	-2.182645	-1.936603
C	-2.546710	-0.843386	-0.884858
C	-3.663607	-1.572828	-1.329866
H	-3.487936	-2.412733	-1.986287
C	-4.941763	-1.265326	-0.905415
O	-6.058674	-1.949155	-1.274231
C	-5.147347	-0.179550	-0.006230
O	-6.436193	0.041255	0.371527
C	-4.068036	0.557098	0.436782
H	-4.202081	1.364325	1.142196
C	-2.753489	0.256904	0.028385
C	-1.581072	1.884539	1.409815
C	0.223179	3.293421	2.489713
C	-0.590940	4.512360	2.925475
H	-0.716913	5.204575	2.085508
H	-0.073769	5.039899	3.734563
H	-1.579993	4.210552	3.278227
C	0.410276	2.313628	3.654392
H	-0.557824	1.960177	4.020972
H	0.937115	2.802574	4.481512
H	0.992858	1.442467	3.333698
C	1.591172	3.747175	1.978985
H	2.202697	2.890482	1.671174
H	2.127462	4.282383	2.768399
H	1.483844	4.417813	1.118617
N	0.498678	-2.017726	-2.388151
H	0.912704	-1.147152	-2.071246
N	-1.242821	-1.073864	-1.236234
N	-1.626292	0.923512	0.408963
N	-0.422526	2.617583	1.354853
H	0.226691	2.299943	0.645319
O	-1.441334	-3.222097	-2.144953
O	-2.453736	2.078302	2.271865
C	3.953271	1.366664	-0.613208
H	4.015656	2.072360	-1.428611
C	5.071025	1.051633	0.135419
O	6.312092	1.563482	-0.085453
C	4.961384	0.142923	1.227581
O	6.110098	-0.098493	1.916730
C	3.738567	-0.420304	1.527530
H	3.633075	-1.094510	2.365588
C	2.584993	-0.116953	0.778362
C	0.982664	-1.373807	2.108802
C	-1.084980	-2.592116	2.908968
C	-0.444105	-3.640696	3.819303
H	-0.088361	-4.491403	3.227355
H	-1.178598	-4.007117	4.544918
H	0.404117	-3.214970	4.360289
C	-1.590809	-1.395741	3.723683
H	-0.765771	-0.914709	4.257158
H	-2.334307	-1.724691	4.458305
H	-2.054063	-0.651208	3.066644
C	-2.251597	-3.220498	2.146614
H	-2.743878	-2.485549	1.499043
H	-2.995162	-3.605139	2.851254
H	-1.906460	-4.052436	1.521829
N	0.161939	1.887779	-2.632367
H	-0.416736	1.143391	-2.258800
N	1.529891	1.044941	-1.006174
N	1.348447	-0.653535	0.978352
N	-0.129543	-2.142903	1.885475
H	-0.530720	-2.029353	0.963182
O	2.161259	2.940267	-2.232359
O	1.558751	-1.330065	3.207102
C	-1.996333	2.392413	-3.525198
H	-1.906104	1.561827	-4.234728
H	-2.646972	3.152774	-3.967471
H	-2.480016	2.017403	-2.614347
C	0.026316	3.503437	-4.492235
H	1.017845	3.912082	-4.282420
H	-0.591393	4.289249	-4.940833
H	0.131182	2.689008	-5.217563
C	-0.788140	4.124770	-2.189748
H	-1.251264	3.750226	-1.270092
H	-1.421445	4.917757	-2.602155
H	0.182764	4.557152	-1.931313
C	-0.625164	2.990516	-3.208306
C	1.361920	2.026682	-1.971081
C	2.695203	0.808138	-0.325011
C	-5.889933	-3.076484	-2.130068
H	-5.250593	-3.837651	-1.663913
H	-5.461185	-2.784398	-3.097490
H	-6.891153	-3.483785	-2.282714
C	-6.677469	1.086359	1.310412
H	-6.380489	2.063416	0.907453
H	-7.754016	1.078790	1.491208
H	-6.145259	0.905058	2.253378
C	6.033151	-0.976262	3.037162
H	5.346609	-0.587911	3.800954
H	7.044452	-1.024163	3.445437
H	5.711459	-1.982099	2.737025
C	6.462578	2.491183	-1.157022
H	5.842024	3.383499	-1.001869
H	6.206513	2.032877	-2.121134
H	7.516697	2.774905	-1.156024

Table S22. Optimized Cartesian Coordinates for $\mathbf{3H_2^+}$ ($\langle S^2 \rangle$ = 0.00).

Cu	-0.260580	0.651465	1.236135
C	-0.699968	-5.067153	3.529250
H	0.349713	-5.208760	3.810696
H	-1.207723	-6.031657	3.617818
H	-1.163719	-4.372391	4.238690
C	-0.143462	-5.532296	1.134452
H	-0.199778	-5.171214	0.104571
H	-0.640064	-6.506155	1.190139
H	0.909958	-5.670914	1.402629
C	-2.289450	-4.348713	1.730371
H	-2.764817	-3.655209	2.432582
H	-2.820444	-5.304789	1.777869
H	-2.389657	-3.944515	0.719825
C	-0.818569	-4.553077	2.097953
C	0.042891	-2.467580	0.994730
C	1.956854	-1.088975	1.018980
C	2.855380	-2.144322	0.710229
H	2.474630	-3.158728	0.684994
C	4.165920	-1.885562	0.423129
O	5.070791	-2.792910	0.070617
C	4.676296	-0.506811	0.459705
O	5.966783	-0.395090	0.161205
C	3.840505	0.534147	0.753069
H	4.216093	1.549949	0.772032
C	2.468322	0.306432	1.025176
C	2.014124	2.609832	1.262523
C	2.212715	4.644901	-0.192209
C	1.652169	5.636346	0.827792
H	0.559793	5.588160	0.856041
H	1.942338	6.652652	0.543065
H	2.035318	5.433257	1.829977
C	3.743158	4.693057	-0.211831
H	4.154594	4.444862	0.770877
H	4.083735	5.698169	-0.480286
H	4.141384	3.988521	-0.950841
C	1.661076	4.962423	-1.579428
H	2.040965	4.259953	-2.329340
H	1.962035	5.972147	-1.871884
H	0.566546	4.915477	-1.589131
N	-0.112130	-3.254523	2.067288
H	0.270188	-2.910637	2.938609
N	0.674717	-1.224288	1.239516
N	1.572975	1.245459	1.245200
N	1.771287	3.267586	0.119281
H	1.380673	2.732089	-0.654697
O	-0.364624	-2.709956	-0.146094
O	2.533303	3.063874	2.280670
C	-3.893536	-1.044648	0.668833
H	-4.351270	-1.144792	1.647452
C	-4.342504	-1.804403	-0.403160
O	-5.326956	-2.724225	-0.332431
C	-3.746178	-1.625465	-1.681809
O	-4.246096	-2.378972	-2.684404
C	-2.708625	-0.717978	-1.831109
H	-2.246949	-0.570529	-2.799486
C	-2.216758	-0.016380	-0.733320
C	-0.033656	0.714328	-1.780753
C	1.300278	-0.922228	-3.113555
C	0.732357	-0.497345	-4.469543
H	-0.242620	-0.965970	-4.644983
H	1.408613	-0.810143	-5.271247
H	0.614609	0.588355	-4.523959
C	2.675597	-0.294157	-2.882752
H	2.622771	0.796219	-2.914165
H	3.369509	-0.635164	-3.657516
H	3.075779	-0.594993	-1.911279
C	1.411071	-2.443645	-3.050656
H	1.805599	-2.769633	-2.082673
H	2.085876	-2.795095	-3.835850
H	0.435153	-2.919592	-3.198556
N	-3.230347	2.560709	0.753540
H	-3.620686	1.950165	0.046483
N	-2.298762	0.576958	1.623315
N	-1.086194	0.859545	-0.818570
N	0.353134	-0.536513	-2.045070
H	-0.032644	-1.293420	-1.474483
O	-2.403700	2.496321	2.897802
O	0.462435	1.753356	-2.228546
C	-4.029120	4.176848	-0.821024
H	-3.300984	3.835539	-1.566456
H	-4.237568	5.232545	-1.013945
H	-4.961506	3.617558	-0.959365
C	-2.172433	4.766967	0.745577
H	-1.748598	4.639197	1.744795
H	-2.337053	5.834917	0.574568
H	-1.440582	4.414192	0.009659
C	-4.524807	4.484052	1.616272
H	-5.460812	3.927327	1.501632
H	-4.734847	5.547020	1.460643
H	-4.159118	4.348814	2.637053
C	-3.490562	4.007752	0.596772
C	-2.676584	1.972481	1.827203
C	-2.823801	-0.166211	0.510560
C	4.648710	-4.159553	-0.051525
H	3.862349	-4.249338	-0.810323
H	4.293839	-4.541772	0.912007
H	5.534482	-4.709385	-0.368326
C	6.544823	0.918753	0.134171
H	6.481149	1.386782	1.122704
H	7.587753	0.770774	-0.144481
H	6.043521	1.542708	-0.615313
C	-3.642636	-2.253496	-3.973827
H	-3.752927	-1.235094	-4.366967
H	-4.177122	-2.952848	-4.617847
H	-2.579629	-2.527494	-3.940210
C	-5.923023	-2.968490	0.943687
H	-6.419322	-2.069105	1.328257
H	-5.175416	-3.319296	1.666038
H	-6.663833	-3.751196	0.775340
H	-2.452080	0.094897	2.508682
H	-1.351484	1.844282	-0.841781

Table S23. Optimized Cartesian Coordinates for $\mathbf{3H_2^+}$ ($<S^2> = 2.00$).

Cu	-0.025307	0.692328	0.907174
C	-0.654196	-4.925316	3.587735
H	0.401402	-5.049466	3.855247
H	-1.136282	-5.904880	3.650199
H	-1.127920	-4.265031	4.322817
C	-0.109361	-5.299539	1.173404
H	-0.170463	-4.900603	0.157704
H	-0.592042	-6.281587	1.189547
H	0.945490	-5.436918	1.437231
C	-2.278549	-4.191563	1.824681
H	-2.765609	-3.529649	2.549216
H	-2.784793	-5.161743	1.851805
H	-2.393594	-3.765676	0.824770
C	-0.799686	-4.369754	2.174759
C	0.023306	-2.240693	1.122946
C	2.086179	-1.039145	1.136642
C	2.889613	-2.187653	1.023243
H	2.428401	-3.163508	1.118084
C	4.246520	-2.078746	0.801364
O	5.083089	-3.117576	0.649232
C	4.849433	-0.774333	0.694941
O	6.174419	-0.774628	0.483696
C	4.064093	0.361484	0.766392
H	4.508985	1.340207	0.642832
C	2.675598	0.262282	0.940932
C	2.206810	2.626110	0.742207
C	1.808871	4.754280	-0.534645
C	1.578864	5.626431	0.699075
H	0.569993	5.479617	1.097134
H	1.688161	6.679897	0.423204
H	2.299461	5.399151	1.487623
C	3.233743	4.930989	-1.069825
H	3.970918	4.650125	-0.312792
H	3.402664	5.976797	-1.346925
H	3.390763	4.311131	-1.959500
C	0.801741	5.120567	-1.623414
H	0.932974	4.495331	-2.513367
H	0.937328	6.165293	-1.916581
H	-0.227370	5.001320	-1.267066
N	-0.128280	-3.051961	2.179220
H	0.299263	-2.746550	3.044061
N	0.758246	-1.054698	1.389623
N	1.783302	1.283285	0.834699
N	1.559660	3.333958	-0.212479
H	1.184689	2.783387	-0.982273
O	-0.453487	-2.438625	0.000822
O	3.053270	3.077613	1.516232
C	-3.731982	-0.992329	0.309262
H	-4.129606	-1.207957	1.295086
C	-4.284269	-1.578851	-0.821024
O	-5.306517	-2.454384	-0.804981
C	-3.757959	-1.254938	-2.104767
O	-4.356439	-1.843868	-3.157883
C	-2.682329	-0.383979	-2.201739
H	-2.261933	-0.136047	-3.169559
C	-2.103804	0.154974	-1.055437
C	0.126981	0.691491	-2.054228
C	1.472531	-1.153925	-3.062060
C	1.043910	-0.890693	-4.506671
H	0.064976	-1.340815	-4.706755
H	1.770463	-1.333256	-5.195184
H	0.986071	0.181541	-4.712429
C	2.847584	-0.546021	-2.779439
H	2.843653	0.534715	-2.937686
H	3.589059	-0.993715	-3.448759
H	3.152905	-0.746029	-1.748758
C	1.510148	-2.656697	-2.794406
H	1.813549	-2.865061	-1.763098
H	2.231750	-3.128161	-3.467159
H	0.530344	-3.116177	-2.965576
N	-3.132669	2.478499	0.933709
H	-3.532284	1.964470	0.157836
N	-2.013432	0.436426	1.347543
N	-0.935137	0.992060	-1.116283
N	0.454820	-0.598063	-2.142729
H	0.018031	-1.252635	-1.488008
O	-1.986709	2.142545	2.899781
O	0.661693	1.643915	-2.623234
C	-4.281821	4.241451	-0.207997
H	-3.609743	4.147405	-1.068881
H	-4.631911	5.276081	-0.165207
H	-5.153606	3.596903	-0.368624
C	-2.337679	4.776696	1.270361
H	-1.793903	4.532378	2.186017
H	-2.647757	5.824376	1.322014
H	-1.658101	4.666248	0.419470
C	-4.519303	4.010974	2.279065
H	-5.391826	3.363808	2.142366
H	-4.866968	5.045203	2.363783
H	-4.020934	3.737508	3.212765
C	-3.565727	3.886732	1.091449
C	-2.411119	1.779641	1.814166
C	-2.637846	-0.138815	0.190156
C	4.527246	-4.435757	0.670492
H	3.786561	-4.558538	-0.129892
H	4.070018	-4.654474	1.643115
H	5.366825	-5.110043	0.499622
C	6.837842	0.484464	0.330225
H	6.718450	1.101965	1.228057
H	7.890969	0.241563	0.185336
H	6.457932	1.020770	-0.547831
C	-3.839894	-1.561158	-4.461424
H	-3.918217	-0.492251	-4.694523
H	-4.459567	-2.133289	-5.152748
H	-2.795507	-1.887479	-4.550130
C	-5.833373	-2.843342	0.467123
H	-6.254023	-1.982497	1.000654
H	-5.060519	-3.322545	1.080998
H	-6.624686	-3.561081	0.248154
H	-2.084581	-0.178448	2.159832
H	-1.172621	1.972096	-1.271229

Table S24. Optimized Cartesian Coordinates for **$3H_2^+$ (B)** (<S^2> = 0.00).

Cu	0.000000	0.000000	0.000000
C	0.000000	0.000000	5.116398
H	1.024513	0.355056	5.273974
H	-0.443955	-0.208499	6.093623
H	-0.575221	0.802409	4.641801
C	0.819205	-2.358185	4.938781
H	0.829862	-3.274019	4.342457
H	0.397901	-2.592079	5.921394
H	1.851021	-2.018063	5.082687
C	-1.457618	-1.732661	4.046398
H	-2.046622	-0.943127	3.567068
H	-1.921398	-1.969794	5.009064
H	-1.492371	-2.626299	3.417848
C	-0.017632	-1.267826	4.267598
C	0.771308	-1.724535	1.932833
C	2.612451	-1.177648	0.526411
C	3.452591	-1.844647	1.459022
H	2.988108	-2.340562	2.301224
C	4.812696	-1.847903	1.344231
O	5.665540	-2.416120	2.197660
C	5.419558	-1.173016	0.213229
O	6.738949	-1.195625	0.184840
C	4.625363	-0.577751	-0.752080
H	5.083490	-0.108336	-1.609865
C	3.236496	-0.572343	-0.655713
C	2.869466	1.083719	-2.473690
C	2.126162	2.460665	-4.411114
C	2.026152	3.798822	-3.672665
H	1.081903	3.863992	-3.121782
H	2.056511	4.623907	-4.391361
H	2.856983	3.925609	-2.974913
C	3.449901	2.354356	-5.171107
H	4.305904	2.459926	-4.501956
H	3.497105	3.147094	-5.924744
H	3.520757	1.388954	-5.683476
C	0.967031	2.327669	-5.395646
H	1.015086	1.375983	-5.935338
H	1.013864	3.141624	-6.124266
H	0.007201	2.377626	-4.876163
N	0.591364	-0.899502	2.969652
H	0.830245	0.084239	2.834560
N	1.304206	-1.048889	0.757929
N	2.473018	0.000000	-1.623741
N	1.984621	1.343234	-3.450199
H	1.118589	0.797316	-3.525832
O	0.515457	-2.920090	1.862265
O	3.909195	1.693619	-2.254512
C	-0.854033	4.045555	-0.686988
H	-0.534523	4.643803	0.159429
C	-1.367529	4.653575	-1.821711
O	-1.454281	5.990676	-2.004949
C	-1.861885	3.831023	-2.876037
O	-2.370990	4.494073	-3.936338
C	-1.784800	2.451912	-2.778349
H	-2.139627	1.828962	-3.589491
C	-1.199896	1.828566	-1.661905
C	-0.990661	-0.407304	-2.587284
C	-0.910898	-2.899709	-2.948827
C	-1.412138	-2.977342	-4.390843
H	-2.506599	-2.948052	-4.416983
H	-1.080097	-3.914438	-4.849949
H	-1.026952	-2.140925	-4.978504
C	0.620819	-2.941194	-2.903758
H	1.055359	-2.111833	-3.469892
H	0.987043	-3.874407	-3.343274
H	0.976666	-2.894991	-1.867538
C	-1.476700	-4.061647	-2.136364
H	-1.119163	-4.029297	-1.099709
H	-1.157149	-5.012158	-2.573039
H	-2.572039	-4.038616	-2.130557
N	1.826088	3.109368	0.575426
H	1.581127	3.356786	-0.374745
N	-0.264234	2.008468	0.573117
N	-1.054971	0.465033	-1.507480
N	-1.404894	-1.669615	-2.295906
H	-1.763332	-1.789875	-1.357388
O	1.044523	2.043658	2.448542
O	-0.522149	-0.090240	-3.696724
C	3.725830	4.445071	-0.040962
H	3.924309	3.810674	-0.910274
H	4.674993	4.875746	0.289251
H	3.068983	5.271606	-0.337727
C	4.034709	2.469521	1.460677
H	3.584617	1.832988	2.227348
H	4.981056	2.858263	1.849662
H	4.250028	1.867221	0.573961
C	2.855580	4.537347	2.301738
H	2.184500	5.360128	2.031814
H	3.803223	4.966550	2.641273
H	2.411781	3.979499	3.129361
C	3.112524	3.633054	1.095897
C	0.926936	2.418000	1.281850
C	-0.762335	2.658356	-0.608343
C	5.120643	-3.095153	3.337212
H	4.481192	-3.927553	3.021593
H	4.554080	-2.398399	3.967207
H	5.980894	-3.474971	3.888030
C	7.435317	-0.515895	-0.876866
H	7.168029	0.545902	-0.887028
H	8.492768	-0.635937	-0.644343
H	7.204591	-0.979673	-1.841055
C	-2.926571	3.728066	-5.007356
H	-2.162636	3.104549	-5.487606
H	-3.301820	4.459290	-5.724579
H	-3.751290	3.096832	-4.655383
C	-0.878419	6.831370	-1.004622
H	0.190099	6.612574	-0.873862
H	-1.398592	6.723121	-0.044614
H	-0.998606	7.851754	-1.370890
H	-0.987080	1.884347	1.285564
H	1.488070	-0.251612	-1.636647

Table S25. Optimized Cartesian Coordinates for $\mathbf{3H_2^+}$ **(B)** ($\langle S^2 \rangle$ = 2.00).

Cu	0.000000	0.000000	0.000000
C	0.000000	0.000000	5.333975
H	0.406270	-1.012128	5.437494
H	-0.236056	0.379126	6.332197
H	0.776115	0.637574	4.897050
C	-2.315120	-0.916658	5.099920
H	-3.221498	-0.951108	4.489677
H	-2.584627	-0.551547	6.096048
H	-1.921750	-1.934185	5.206378
C	-1.792232	1.430603	4.337099
H	-1.033746	2.077903	3.883502
H	-2.038304	1.830240	5.326098
H	-2.692267	1.459528	3.718282
C	-1.262189	0.002796	4.475645
C	-1.676365	-0.678783	2.097955
C	-1.134238	-2.561978	0.751372
C	-1.751009	-3.388926	1.726809
H	-2.211016	-2.917180	2.585510
C	-1.729919	-4.757683	1.645344
O	-2.242878	-5.595940	2.563297
C	-1.105199	-5.380449	0.512887
O	-1.086259	-6.718335	0.517730
C	-0.581065	-4.589036	-0.498333
H	-0.140400	-5.050843	-1.370821
C	-0.580874	-3.201058	-0.418335
C	1.118779	-2.797876	-2.171387
C	2.555964	-2.061449	-4.077979
C	3.867373	-2.013766	-3.286348
H	3.920985	-1.096163	-2.694268
H	4.721226	-2.026926	-3.971473
H	3.948024	-2.871881	-2.615012
C	2.465191	-3.361444	-4.880749
H	2.499496	-4.230210	-4.218917
H	3.304600	-3.423958	-5.581200
H	1.532996	-3.393455	-5.454790
C	2.480870	-0.867851	-5.028027
H	1.550846	-0.882986	-5.606834
H	3.321433	-0.895438	-5.726994
H	2.524694	0.076203	-4.475705
N	-0.864466	-0.520668	3.151899
H	0.098786	-0.846654	3.053634
N	-1.017308	-1.232626	0.941745
N	-0.023034	-2.438097	-1.430870
N	1.396994	-1.937752	-3.177011
H	0.861305	-1.073102	-3.233779
O	-2.869216	-0.383642	2.037919
O	1.783495	-3.791626	-1.884796
C	4.434059	0.381895	-0.031486
H	4.933919	-0.084065	0.809742
C	5.180611	0.872540	-1.100654
O	6.503572	0.769256	-1.207297
C	4.506337	1.553376	-2.172850
O	5.297038	1.999746	-3.158107
C	3.137866	1.677823	-2.140002
H	2.629494	2.175950	-2.955508
C	2.360888	1.126678	-1.091043
C	0.335236	1.522809	-2.298034
C	-0.475742	3.509390	-3.599065
C	0.304549	3.194510	-4.877887
H	1.339654	3.545699	-4.798485
H	-0.159254	3.705841	-5.726869
H	0.309741	2.121166	-5.084487
C	-1.930640	3.054250	-3.718460
H	-1.993356	1.983296	-3.926001
H	-2.416401	3.594352	-4.537153
H	-2.476935	3.265893	-2.793428
C	-0.415123	5.004029	-3.302484
H	-0.960364	5.245064	-2.383179
H	-0.872638	5.560739	-4.124784
H	0.621317	5.344501	-3.197159
N	3.159175	-2.178796	1.108773
H	3.397456	-2.012722	0.140659
N	2.307887	0.000000	1.078646
N	1.011127	1.113063	-1.104276
N	0.171689	2.838322	-2.448190
H	0.517775	3.426245	-1.700589
O	2.000439	-1.322747	2.893478
O	-0.062846	0.653255	-3.080729
C	4.345545	-4.185225	0.560548
H	3.786111	-4.272611	-0.377227
H	4.628809	-5.190551	0.884637
H	5.266662	-3.618386	0.378495
C	2.219068	-4.332127	1.867765
H	1.556858	-3.832671	2.580174
H	2.475045	-5.319802	2.265278
H	1.685454	-4.465506	0.923833
C	4.300028	-3.381997	2.929853
H	5.206269	-2.790116	2.759913
H	4.599716	-4.375291	3.278678
H	3.710262	-2.902733	3.714733
C	3.493107	-3.518657	1.638360
C	2.497166	-1.210939	1.767105
C	3.056051	0.497379	-0.001226
C	-2.863378	-5.022562	3.717325
H	-3.724130	-4.404448	3.434430
H	-2.145013	-4.423532	4.291685
H	-3.199045	-5.867381	4.319665
C	-0.398058	-7.396483	-0.541910
H	0.650148	-7.078847	-0.587476
H	-0.454852	-8.456129	-0.291675
H	-0.888272	-7.215002	-1.504874
C	4.681051	2.665354	-4.266246
H	3.996568	1.990317	-4.794666
H	5.499559	2.947175	-4.928492
H	4.146053	3.563471	-3.934980
C	7.222318	0.050436	-0.192097
H	6.867620	-0.985186	-0.130646
H	7.120965	0.546440	0.779098
H	8.264148	0.065991	-0.510635
H	1.906704	0.699589	1.696410
H	-0.524392	-1.603189	-1.716276

Table S26. Optimized Cartesian Coordinates for **2**$^{2-}$ (<S^2> = 0.75).

Cu	-0.063364	0.045672	0.350786
C	-2.577265	-3.591590	1.968912
H	-3.066350	-2.785610	2.528110
H	-3.108982	-4.526387	2.172740
H	-2.672223	-3.366783	0.899742
C	-1.019532	-4.027772	3.869506
H	0.023892	-4.111032	4.182094
H	-1.533310	-4.970196	4.091774
H	-1.494419	-3.229811	4.451665
C	-0.451357	-4.836788	1.554056
H	-0.523733	-4.610918	0.484302
H	-0.944632	-5.796241	1.747850
H	0.607416	-4.931412	1.812934
C	-1.107177	-3.718350	2.374533
C	0.870413	-2.175945	2.058876
C	2.534048	-0.774796	0.950106
C	3.686274	-1.486098	1.328797
H	3.542594	-2.407707	1.874397
C	4.961677	-1.037185	1.020458
O	6.115051	-1.707574	1.356743
C	5.120574	0.176912	0.322411
O	6.412536	0.575822	0.069491
C	3.994611	0.877550	-0.081568
H	4.098401	1.786091	-0.658707
C	2.695467	0.415592	0.181081
C	1.489712	1.873619	-1.331754
C	-0.342375	2.961526	-2.677411
C	0.457945	4.017211	-3.440687
H	0.599050	4.907834	-2.817444
H	-0.078742	4.312602	-4.349797
H	1.440870	3.629379	-3.715795
C	-0.546326	1.705882	-3.534912
H	0.419339	1.262634	-3.796826
H	-1.080669	1.948633	-4.461078
H	-1.124485	0.955176	-2.983758
C	-1.705512	3.543858	-2.298515
H	-2.321701	2.806317	-1.771558
H	-2.244010	3.856855	-3.199171
H	-1.585723	4.416678	-1.646449
N	-0.501889	-2.409847	2.075855
H	-0.980415	-1.940366	1.310246
N	1.226471	-1.169982	1.204912
N	1.539320	1.056121	-0.236337
N	0.319299	2.618730	-1.406901
H	-0.360282	2.318388	-0.714694
O	1.638146	-2.815532	2.811086
O	2.380190	1.982322	-2.201450
C	-3.934022	1.588780	0.269854
H	-3.939374	2.516936	0.823352
C	-5.080562	1.138535	-0.367400
O	-6.280227	1.812435	-0.361713
C	-5.047359	-0.080236	-1.074614
O	-6.219378	-0.474285	-1.677330
C	-3.859836	-0.791249	-1.143459
H	-3.806035	-1.704997	-1.718661
C	-2.679138	-0.332508	-0.538736
C	-1.129604	-1.854113	-1.616754
C	0.972638	-3.023963	-2.371381
C	0.381088	-4.120320	-3.257874
H	0.057209	-4.968534	-2.643752
H	1.134586	-4.476779	-3.969822
H	-0.482509	-3.744747	-3.810547
C	1.422376	-1.824310	-3.215306
H	0.570849	-1.394562	-3.751692
H	2.179591	-2.126409	-3.948554
H	1.847559	-1.043078	-2.574148
C	2.175079	-3.589731	-1.612942
H	2.644310	-2.825500	-0.982949
H	2.926066	-3.958047	-2.319605
H	1.869235	-4.421741	-0.968373
N	-0.079273	2.572572	2.033887
H	0.578376	2.084111	1.429569
N	-1.528866	1.277801	0.804637
N	-1.458739	-0.983707	-0.613335
N	0.008279	-2.601162	-1.341991
H	0.486679	-2.266438	-0.511125
O	-2.337759	2.965745	2.202099
O	-1.757812	-2.006376	-2.686025
C	1.946694	3.796165	2.375704
H	2.300181	3.019129	3.063373
H	2.401478	4.748516	2.666303
H	2.296403	3.539838	1.368554
C	-0.025539	4.264116	3.832873
H	-1.115118	4.323440	3.886215
H	0.397955	5.230526	4.129881
H	0.316831	3.503654	4.544067
C	-0.033842	4.978316	1.418389
H	0.293109	4.714477	0.406345
H	0.390168	5.953568	1.683961
H	-1.124544	5.062319	1.415551
C	0.420778	3.904167	2.415448
C	-1.402647	2.315129	1.685593
C	-2.724161	0.873346	0.223116
C	5.968987	-2.973471	1.985346
H	5.404805	-3.674832	1.354697
H	5.468809	-2.888746	2.960298
H	6.982875	-3.353009	2.132629
C	6.590064	1.794139	-0.640137
H	6.162767	2.647402	-0.095011
H	7.669899	1.930180	-0.734844
H	6.140558	1.749408	-1.642116
C	-6.190895	-1.682696	-2.424755
H	-5.469585	-1.630465	-3.252391
H	-7.197657	-1.807082	-2.830523
H	-5.945218	-2.546738	-1.791612
C	-6.314243	3.071094	0.296964
H	-5.603908	3.780926	-0.149984
H	-6.095021	2.974834	1.369609
H	-7.331768	3.447954	0.169222

Table S27. Optimized Cartesian Coordinates for **2**$^{2-}$ ($<S^2>$ = 3.76).

Cu	-0.003720	-0.006281	0.265794
C	-1.665631	-3.847199	2.256636
H	-2.171017	-3.110455	2.891993
H	-2.031724	-4.843304	2.524335
H	-1.947988	-3.645190	1.215756
C	0.197097	-4.045859	3.907753
H	1.276676	-3.990608	4.064869
H	-0.152647	-5.043180	4.197697
H	-0.287615	-3.307529	4.556584
C	0.528589	-4.801986	1.527682
H	0.280997	-4.595778	0.480173
H	0.196511	-5.815912	1.777943
H	1.616147	-4.755273	1.637559
C	-0.148539	-3.774341	2.443248
C	1.528800	-1.961135	1.887523
C	2.756042	-0.290870	0.592826
C	4.064770	-0.726372	0.891382
H	4.179287	-1.602218	1.510266
C	5.175997	-0.067365	0.351744
O	6.473785	-0.462018	0.577141
C	5.004802	1.081214	-0.453223
O	6.156713	1.682721	-0.895866
C	3.730784	1.538088	-0.755780
H	3.580312	2.374485	-1.421167
C	2.583748	0.832692	-0.289835
C	0.943246	2.007321	-1.642030
C	-1.254330	2.776527	-2.610366
C	-0.790988	3.952013	-3.470782
H	-0.692707	4.854755	-2.856862
H	-1.521771	4.150537	-4.263305
H	0.178134	3.736905	-3.926339
C	-1.391432	1.502862	-3.454670
H	-0.428213	1.234439	-3.899629
H	-2.118747	1.649981	-4.262189
H	-1.722614	0.663373	-2.832766
C	-2.602698	3.117179	-1.973937
H	-2.978391	2.287895	-1.362788
H	-3.343027	3.329346	-2.752324
H	-2.513729	4.000941	-1.331482
N	0.229278	-2.404892	2.062273
H	-0.423985	-1.990529	1.403612
N	1.592466	-0.879154	1.031115
N	1.298924	1.185880	-0.586326
N	-0.323377	2.554277	-1.488623
H	-0.807211	2.155549	-0.691380
O	2.481292	-2.493400	2.490967
O	1.642564	2.228832	-2.653846
C	-4.064951	0.743425	0.884511
H	-4.174592	1.628981	1.490057
C	-5.181052	0.084745	0.349454
O	-6.476756	0.492545	0.567010
C	-5.016247	-1.078429	-0.435880
O	-6.172187	-1.676902	-0.872392
C	-3.746261	-1.553430	-0.725619
H	-3.600992	-2.399830	-1.378960
C	-2.592739	-0.844654	-0.272331
C	-0.957685	-2.059870	-1.597094
C	1.239077	-2.815015	-2.575656
C	0.788254	-4.021871	-3.398564
H	0.719440	-4.910115	-2.760011
H	1.510523	-4.225316	-4.197690
H	-0.192465	-3.839175	-3.843285
C	1.336331	-1.561007	-3.454342
H	0.360421	-1.323222	-3.888935
H	2.052335	-1.714720	-4.270756
H	1.661269	-0.698726	-2.860724
C	2.604111	-3.111093	-1.952814
H	2.971981	-2.258598	-1.369692
H	3.336200	-3.328990	-2.737419
H	2.543913	-3.978778	-1.285552
N	-0.221321	2.423391	2.018035
H	0.428909	1.997891	1.363563
N	-1.593102	0.885088	1.018961
N	-1.310330	-1.208638	-0.564092
N	0.319994	-2.584485	-1.445400
H	0.808199	-2.156498	-0.666078
O	-2.470198	2.524810	2.457752
O	-1.666985	-2.326016	-2.592612
C	1.682423	3.855124	2.199200
H	2.185127	3.118964	2.837321
H	2.054873	4.850590	2.460291
H	1.960975	3.645575	1.158814
C	-0.174745	4.074649	3.854023
H	-1.254220	4.027869	4.014350
H	0.182748	5.071179	4.137057
H	0.306572	3.336790	4.505898
C	-0.508341	4.817586	1.469745
H	-0.262163	4.604876	0.423174
H	-0.172127	5.831594	1.714008
H	-1.595932	4.775577	1.580892
C	0.165452	3.792101	2.390246
C	-1.522112	1.980515	1.858487
C	-2.759908	0.295218	0.592272
C	6.673705	-1.687751	1.268634
H	6.185272	-2.525300	0.751729
H	6.301393	-1.638987	2.300951
H	7.754237	-1.848861	1.283544
C	6.017872	2.818004	-1.741514
H	5.485446	3.635076	-1.235977
H	7.034635	3.139473	-1.978598
H	5.488348	2.566146	-2.670405
C	-6.041165	-2.821722	-1.706426
H	-5.507170	-2.583475	-2.636304
H	-7.060120	-3.136836	-1.942695
H	-5.516894	-3.638264	-1.191590
C	-6.668777	1.731746	1.236035
H	-6.174121	2.556721	0.704844
H	-6.298143	1.699283	2.269586
H	-7.748196	1.900740	1.246837

Table S28. Optimized Cartesian Coordinates for **$2H_3^+$** (<S^2> = 0.75).

Cu	-0.153249	-0.918024	-1.426399
C	-2.043826	4.987811	-3.336001
H	-1.163733	5.297820	-3.911560
H	-2.685047	5.863664	-3.203737
H	-2.598977	4.242361	-3.916479
C	-0.847477	5.488546	-1.194792
H	-0.540905	5.104148	-0.218782
H	-1.466571	6.377971	-1.039512
H	0.046389	5.786583	-1.753995
C	-2.886786	4.010017	-1.195981
H	-3.452097	3.265916	-1.764736
H	-3.531451	4.877788	-1.025712
H	-2.621446	3.576993	-0.229006
C	-1.642075	4.434612	-1.971709
C	-0.173884	2.509643	-1.309210
C	1.941264	1.304801	-1.286983
C	2.682630	2.400871	-0.881566
H	2.243754	3.389002	-0.961924
C	3.971563	2.258481	-0.374566
O	4.724610	3.264062	0.086250
C	4.563573	0.953942	-0.322224
O	5.812918	0.903117	0.174465
C	3.829279	-0.135860	-0.724653
H	4.261811	-1.126544	-0.656778
C	2.488182	-0.009854	-1.164899
C	2.308545	-2.374477	-1.357077
C	2.674267	-4.353003	0.148450
C	2.214960	-5.408121	-0.858263
H	1.122256	-5.453068	-0.899581
H	2.586362	-6.390907	-0.551184
H	2.592776	-5.191639	-1.859809
C	4.202612	-4.267839	0.190555
H	4.608096	-3.996962	-0.788217
H	4.623022	-5.236636	0.478751
H	4.527223	-3.524059	0.926998
C	2.135781	-4.692494	1.535715
H	2.442054	-3.945112	2.275579
H	2.521947	-5.665792	1.850679
H	1.041460	-4.743966	1.534240
N	-0.787538	3.257875	-2.245323
H	-0.492055	3.142253	-3.205109
N	0.634351	1.460505	-1.816967
N	1.698800	-1.079283	-1.410163
N	2.110993	-3.029735	-0.200783
H	1.676192	-2.512091	0.560173
O	-0.298637	2.661987	-0.093390
O	2.923319	-2.795534	-2.336271
C	-3.930806	0.483891	-0.766365
H	-4.436794	0.387790	-1.721433
C	-4.447133	1.300954	0.228443
O	-5.567293	2.042598	0.109162
C	-3.761085	1.399270	1.470314
O	-4.309662	2.221447	2.389092
C	-2.601092	0.668057	1.672710
H	-2.086943	0.714099	2.624123
C	-2.065615	-0.117251	0.653416
C	0.173667	-0.596680	1.738752
C	1.350402	1.200995	3.007605
C	0.740645	0.830900	4.361825
H	-0.263056	1.258200	4.467005
H	1.363488	1.225526	5.170779
H	0.674316	-0.254339	4.479597
C	2.769739	0.643919	2.890043
H	2.776201	-0.442740	2.997365
H	3.401059	1.080119	3.670824
H	3.201217	0.896995	1.919485
C	1.377170	2.719615	2.850945
H	1.790999	3.004869	1.878816
H	1.998692	3.159070	3.635821
H	0.371052	3.146498	2.929254
N	-2.919000	-2.957349	-0.526804
H	-3.306132	-2.301654	0.141225
N	-2.161819	-1.002762	-1.619578
N	-0.866520	-0.873953	0.805482
N	0.478972	0.691291	1.926739
H	0.087114	1.380494	1.279839
O	-2.178070	-3.047927	-2.701123
O	0.738715	-1.571950	2.247561
C	-3.570430	-4.458746	1.220467
H	-2.817029	-4.033014	1.893728
H	-3.731544	-5.500122	1.511458
H	-4.513973	-3.919184	1.361638
C	-1.776353	-5.121210	-0.391279
H	-1.411546	-5.066506	-1.419969
H	-1.894900	-6.175141	-0.123356
H	-1.020366	-4.685780	0.271985
C	-4.178368	-4.988961	-1.153240
H	-5.127777	-4.456357	-1.034765
H	-4.339572	-6.041309	-0.899135
H	-3.870617	-4.929129	-2.200144
C	-3.111963	-4.395349	-0.233436
C	-2.449202	-2.446755	-1.674015
C	-2.732778	-0.200606	-0.566274
C	4.158894	4.579989	0.136104
H	3.260204	4.592052	0.764618
H	3.922018	4.943485	-0.870086
H	4.928353	5.208930	0.584149
C	6.416884	-0.384155	0.329287
H	6.528312	-0.887624	-0.638720
H	7.400501	-0.195820	0.760716
H	5.829610	-1.011065	1.012564
C	-3.611924	2.398719	3.623414
H	-3.543816	1.456463	4.181606
H	-4.201633	3.117504	4.193541
H	-2.606088	2.804545	3.451460
C	-6.282188	1.978567	-1.126092
H	-6.634408	0.959423	-1.327669
H	-5.662062	2.327703	-1.961425
H	-7.137054	2.645004	-1.004689
H	-2.414494	-0.631994	-2.536738
H	-1.041459	-1.875262	0.877472
H	0.610236	1.349793	-2.828552

Table S29. Optimized Cartesian Coordinates for **2H$_3^+$** ($\langle S^2 \rangle$ = 3.76).

Cu	-0.289484	-0.861575	-1.490589
C	-2.131819	4.984460	-3.319577
H	-1.264032	5.271544	-3.924863
H	-2.774297	5.862982	-3.213878
H	-2.694740	4.212193	-3.855920
C	-0.901485	5.582488	-1.222209
H	-0.571579	5.238430	-0.238599
H	-1.525011	6.472345	-1.088156
H	-0.021684	5.865188	-1.810723
C	-2.929555	4.093795	-1.124345
H	-3.488953	3.306518	-1.639523
H	-3.588432	4.957723	-0.993524
H	-2.640263	3.726515	-0.138311
C	-1.702605	4.492676	-1.940151
C	-0.220678	2.591996	-1.226053
C	1.861307	1.334156	-1.200592
C	2.632236	2.412210	-0.802873
H	2.214560	3.410064	-0.871408
C	3.931833	2.238886	-0.335983
O	4.718452	3.223630	0.108203
C	4.503576	0.921382	-0.320001
O	5.768378	0.846229	0.126835
C	3.735183	-0.151613	-0.700212
H	4.149128	-1.150779	-0.648496
C	2.378580	0.004822	-1.082427
C	2.146851	-2.349017	-1.278784
C	2.682598	-4.307126	0.194220
C	2.211586	-5.383563	-0.784101
H	1.122344	-5.477938	-0.761738
H	2.644351	-6.347483	-0.497997
H	2.519854	-5.153480	-1.805906
C	4.205750	-4.156282	0.138806
H	4.534937	-3.865130	-0.863009
H	4.685575	-5.107049	0.391840
H	4.545955	-3.402914	0.858414
C	2.247132	-4.669859	1.611162
H	2.570360	-3.912058	2.333065
H	2.690818	-5.627720	1.896554
H	1.157608	-4.762911	1.677867
N	-0.848152	3.310045	-2.177568
H	-0.588560	3.141105	-3.139597
N	0.560876	1.513804	-1.724887
N	1.551949	-1.041783	-1.271998
N	2.047869	-3.005232	-0.113293
H	1.640329	-2.501949	0.674176
O	-0.303321	2.781115	-0.014306
O	2.672484	-2.765723	-2.310259
C	-4.093226	0.303397	-0.608014
H	-4.673653	0.060665	-1.492327
C	-4.475815	1.345732	0.206972
O	-5.543424	2.116063	0.017194
C	-3.678329	1.694214	1.392367
O	-4.105251	2.773643	2.082658
C	-2.536148	0.956368	1.681538
H	-1.966332	1.182006	2.574980
C	-2.127135	-0.088151	0.880923
C	0.115143	-0.650040	1.919835
C	1.421324	1.118267	3.117415
C	0.885161	0.747588	4.502664
H	-0.107271	1.183063	4.664034
H	1.557184	1.128394	5.278404
H	0.813269	-0.338271	4.614902
C	2.822245	0.537141	2.919336
H	2.812172	-0.550665	3.013685
H	3.502386	0.952383	3.670220
H	3.206295	0.792749	1.930421
C	1.467905	2.637348	2.968488
H	1.815119	2.921437	1.970185
H	2.152278	3.059977	3.709182
H	0.478902	3.083615	3.122363
N	-2.905035	-3.179184	-0.568359
H	-3.355629	-2.560157	0.105975
N	-2.264550	-1.085118	-1.441026
N	-1.031343	-0.883416	1.150964
N	0.476470	0.634994	2.090832
H	0.057400	1.334606	1.479858
O	-2.019199	-3.034759	-2.689220
O	0.728099	-1.642457	2.345506
C	-3.646177	-4.837275	0.992764
H	-2.971616	-4.437186	1.758374
H	-3.783848	-5.904606	1.185590
H	-4.620907	-4.346207	1.091859
C	-1.701736	-5.308748	-0.509355
H	-1.255529	-5.150299	-1.494482
H	-1.802463	-6.385906	-0.345978
H	-1.022671	-4.909943	0.252109
C	-4.034343	-5.183896	-1.459947
H	-5.008556	-4.689640	-1.383454
H	-4.179611	-6.257636	-1.304962
H	-3.641188	-5.030232	-2.468200
C	-3.070473	-4.638072	-0.406239
C	-2.403357	-2.553137	-1.634697
C	-2.939956	-0.468238	-0.320809
C	4.175996	4.547751	0.203791
H	3.295101	4.558312	0.856681
H	3.918302	4.937166	-0.787336
H	4.968139	5.153451	0.643994
C	6.359704	-0.451515	0.242935
H	6.421478	-0.945805	-0.734093
H	7.363327	-0.281607	0.633769
H	5.792051	-1.076291	0.944398
C	-3.294316	3.227605	3.165258
H	-3.244269	2.477704	3.964950
H	-3.778687	4.129745	3.540690
H	-2.280269	3.466949	2.816852
C	-6.369864	1.880422	-1.130613
H	-6.796940	0.872421	-1.098628
H	-5.789809	2.013804	-2.051416
H	-7.161654	2.627365	-1.076829
H	-2.582302	-0.672689	-2.327396
H	-1.126407	-1.876912	0.947202
H	0.527159	1.384438	-2.733974

Table S30. Optimized Cartesian Coordinates for **$1H_4^+$** ($\langle S^2 \rangle = 0.00$).

Cu	0.056678	2.535028	0.288991
N	3.449457	-0.523677	-0.319173
H	1.627062	-5.541963	2.138278
C	3.617452	-5.511902	-1.020023
H	4.427292	-0.669936	-0.534495
H	-4.437587	-0.614402	0.476532
H	3.729375	-5.275391	0.046454
H	4.441784	-5.056811	-1.583977
H	3.637365	-6.594669	-1.154912
C	-1.330833	-4.017743	-2.811862
H	4.470110	2.357808	-0.956197
N	0.829408	0.266357	-1.399670
H	4.764391	3.836559	-1.892277
C	-2.596817	-1.507456	0.698863
C	-3.011664	-2.829337	0.493825
H	-3.981194	-2.989100	0.032773
C	-2.201277	-3.910842	0.800295
O	-2.530308	-5.209524	0.585254
C	-0.910826	-3.670282	1.335923
O	-0.141635	-4.763143	1.563419
C	-0.521180	-2.366973	1.588857
H	0.459253	-2.155668	2.002095
C	-1.348828	-1.275819	1.291420
H	-1.822157	-4.960244	-3.060781
H	3.207803	3.596372	-1.075186
C	1.173599	-4.551125	2.072647
H	-0.524457	-1.708585	-2.293279
C	0.788370	-3.372274	-1.941959
O	-0.022564	-4.372002	-2.367374
H	1.762530	-3.919373	1.395825
C	2.069920	-3.758502	-1.474069
O	2.350664	-5.085259	-1.516601
C	2.920407	-2.786703	-0.971320
H	3.883352	-3.067153	-0.556341
C	2.555318	-1.435915	-0.915361
H	-1.290057	-3.380314	-3.704520
C	1.317443	-1.048687	-1.444185
C	-3.806174	-5.484621	0.010761
N	-3.454103	-0.464834	0.291270
N	-0.811322	0.004086	1.498974
H	1.146475	-4.095718	3.070991
H	-1.892683	-3.506347	-2.020311
H	-0.172916	0.361648	-1.208220
C	0.448283	-2.031313	-1.937705
H	0.195300	0.096764	1.329408
C	1.460688	1.419458	-1.746013
C	3.159653	0.128495	0.869705
C	-1.400006	1.090440	2.065795
C	-3.135859	0.392238	-0.751169
O	2.039394	0.079529	1.390044
O	1.019588	2.539466	-1.322377
O	-2.014279	0.405944	-1.270725
O	-0.912872	2.253669	1.871079
N	2.523365	1.349046	-2.551464
H	2.722579	0.433298	-2.931326
N	-4.144099	1.218809	-1.123400
H	-5.032515	1.126055	-0.652632
N	-2.471096	0.905188	2.841428
H	-2.708744	-0.058723	3.033791
N	4.195275	0.835389	1.385372
H	5.077459	0.810348	0.895018
C	-4.050156	2.297344	-2.121967
C	-5.419057	2.974222	-2.134777
H	-5.424580	3.788562	-2.864633
H	-6.207193	2.264324	-2.411800
H	-5.658278	3.397845	-1.152125
C	-3.745299	1.717146	-3.505438
H	-3.710242	2.520995	-4.248315
H	-2.782368	1.200226	-3.502943
H	-4.524628	1.006897	-3.802619
C	-2.979379	3.313138	-1.711971
H	-3.194108	3.718137	-0.717364
H	-1.987952	2.852030	-1.691170
H	-2.960126	4.142937	-2.426110
C	4.139559	1.706403	2.571367
C	5.531761	2.319950	2.702433
H	6.293735	1.542814	2.834044
H	5.785771	2.913030	1.815903
H	5.567189	2.980903	3.572961
C	3.814021	0.884654	3.821176
H	3.814561	1.532204	4.704361
H	2.830157	0.417706	3.729468
H	4.563172	0.099000	3.968348
C	3.104696	2.817585	2.371224
H	2.097751	2.401976	2.273958
H	3.116796	3.497430	3.229492
H	3.329929	3.395741	1.468868
C	-3.394051	1.924996	3.375284
C	-3.886297	2.846440	2.259092
H	-4.367925	2.268581	1.465164
H	-4.617307	3.552036	2.665251
H	-3.063428	3.414948	1.819908
C	-4.572226	1.145280	3.955498
H	-4.242306	0.463270	4.747761
H	-5.300504	1.837128	4.387054
H	-5.077156	0.560847	3.177602
C	-2.699110	2.726527	4.477777
H	-2.357179	2.061834	5.278260
H	-1.835747	3.265854	4.079018
H	-3.396375	3.453511	4.906908
C	3.481288	2.419438	-2.888485
C	2.811901	3.440375	-3.810857
H	3.532021	4.213465	-4.098346
H	2.446521	2.953015	-4.721011
H	1.967821	3.920507	-3.308420
C	4.629629	1.725382	-3.617855
H	5.121019	0.991898	-2.968008
H	4.272880	1.212133	-4.518387
H	5.376537	2.462973	-3.923449
C	4.008576	3.093073	-1.621584
H	-4.619947	-5.117189	0.649052
H	-3.866229	-6.571561	-0.067036
H	-3.897946	-5.042039	-0.989988

Table S31. Optimized Cartesian Coordinates for **$1H_4^+$** (<S^2> = 2.00).

Cu	0.000000	0.000000	0.000000
N	0.000000	0.000000	4.714660
H	4.676660	3.437962	6.223873
C	1.408592	3.391329	8.183479
H	-0.350061	-0.427517	5.562236
H	2.466961	4.772904	-1.274043
H	2.201814	2.649326	8.021371
H	0.508704	2.888390	8.559871
H	1.746540	4.129201	8.913003
C	0.878362	6.213242	3.535327
H	-2.009568	-2.181862	3.541980
N	-0.638116	1.619367	2.354798
H	-3.539824	-2.822487	2.908964
C	2.969441	3.838753	0.449161
C	3.900374	4.826127	0.811508
H	4.086114	5.647294	0.121714
C	4.474036	4.832661	2.061973
O	5.399320	5.709346	2.500921
C	4.064240	3.819665	3.055781
O	4.521062	4.046570	4.304952
C	3.291978	2.760329	2.659636
H	3.053660	1.947985	3.342896
C	2.852963	2.648452	1.324670
H	1.275041	7.200816	3.777597
H	-2.370124	-2.064138	1.811016
C	4.255872	3.051885	5.294039
H	-0.148196	4.097345	2.675440
C	0.509680	4.183633	4.719758
O	0.855541	5.494737	4.768944
H	3.178434	2.889805	5.409306
C	0.689568	3.435791	5.911125
O	1.134321	4.125959	6.992098
C	0.477359	2.065727	5.880207
H	0.697164	1.461448	6.754640
C	0.063604	1.406658	4.715735
H	-0.128921	6.320923	3.112831
C	-0.235818	2.168868	3.579524
C	5.818085	6.737436	1.602319
N	2.120545	4.086756	-0.613383
N	2.451983	1.413686	0.867064
H	4.742644	2.104108	5.030630
H	1.533649	5.719990	2.804670
H	-0.267590	2.089282	1.523025
C	0.015117	3.546841	3.596136
H	2.089462	0.773874	1.583589
C	-1.590082	0.685036	2.099342
C	0.842700	-0.768194	3.921116
C	2.531193	0.891362	-0.396701
C	0.788810	3.722517	-0.751720
O	1.603093	-0.260923	3.088804
O	-1.604611	0.091024	0.970120
O	0.132511	3.231689	0.177959
O	1.687553	-0.000000	-0.785929
N	-2.511231	0.428629	3.031882
H	-2.488373	1.027764	3.845997
N	0.292899	3.941418	-2.000198
H	0.901453	4.378256	-2.67720
N	3.520133	1.345375	-1.193749
H	4.269091	1.835397	-0.724884
N	0.727258	-2.103995	4.119948
H	0.111428	-2.423060	4.853871
C	-1.110657	3.788161	-2.410915
C	-1.159038	4.195363	-3.882329
H	-2.180260	4.101337	-4.262069
H	-0.842933	5.236827	-4.014764
H	-0.510031	3.552003	-4.487843
C	-2.017214	4.705332	-1.584589
H	-3.055518	4.604607	-1.917885
H	-1.965881	4.445657	-0.523846
H	-1.716328	5.751762	-1.705171
C	-1.550979	2.328011	-2.270100
H	-0.876476	1.668360	-2.826442
H	-1.549807	2.018590	-1.222011
H	-2.563978	2.205083	-2.667852
C	1.476169	-3.149269	3.402957
C	0.995918	-4.477784	3.983141
H	1.205590	-4.536812	5.057656
H	-0.081566	-4.608604	3.828603
H	1.512063	-5.307095	3.491375
C	2.980300	-2.992954	3.642432
H	3.524284	-3.801738	3.143247
H	3.337369	-2.037488	3.250209
H	3.203630	-3.037617	4.714061
C	1.155182	-3.096195	1.906367
H	1.471247	-2.145056	1.469433
H	1.673939	-3.907384	1.384908
H	0.078655	-3.211097	1.740829
C	3.721005	1.071624	-2.624154
C	2.453557	1.388190	-3.418387
H	2.167495	2.434845	-3.287673
H	2.635839	1.210213	-4.482692
H	1.616933	0.762719	-3.098830
C	4.850066	2.000255	-3.068293
H	5.768411	1.803310	-2.502406
H	5.068595	1.843137	-4.128216
H	4.571117	3.050750	-2.927644
C	4.134471	-0.389822	-2.826051
H	5.052122	-0.608620	-2.269302
H	3.346938	-1.064288	-2.478703
H	4.319735	-0.586527	-3.887373
C	-3.493506	-0.672245	3.056756
C	-4.583637	-0.416587	2.013833
H	-5.333672	-1.213310	2.053604
H	-5.083745	0.538225	2.208173
H	-4.157639	-0.389292	1.007270
C	-4.095410	-0.651725	4.460425
H	-3.326373	-0.833226	5.220195
H	-4.575356	0.311278	4.670422
H	-4.854279	-1.433610	4.551091
C	-2.806616	-2.016112	2.811130
H	6.251298	6.308216	0.689860
H	6.575140	7.310262	2.139497
H	4.977608	7.391878	1.338693

Table S32. Optimized Cartesian Coordinates for **$1H_4^+$(B)** ($\langle S^2 \rangle$ = 0.00).

Cu	-0.124166	0.260428	-0.422333
C	-5.125777	4.056617	0.582736
H	-5.753049	3.281824	1.038508
H	-5.785212	4.831063	0.181535
H	-4.501610	4.509256	1.361097
C	-5.164581	2.833393	-1.600818
H	-4.570318	2.400524	-2.409882
H	-5.829628	3.587895	-2.032221
H	-5.783635	2.047123	-1.154968
C	-3.399724	4.578736	-1.152698
H	-2.773096	5.046385	-0.386703
H	-4.041315	5.349696	-1.590541
H	-2.753168	4.179321	-1.937358
C	-4.270324	3.481818	-0.541318
C	-2.532853	1.677433	-0.564100
C	-2.706488	-0.580971	0.374668
C	-3.681445	-0.856081	-0.586020
H	-3.853090	-0.139559	-1.379863
C	-4.391224	-2.045417	-0.583618
O	-5.323742	-2.380487	-1.506901
C	-4.137827	-2.996224	0.435792
O	-4.855438	-4.144277	0.377994
C	-3.191912	-2.701221	1.402624
H	-2.979061	-3.411143	2.194887
C	-2.455507	-1.508646	1.388986
C	-0.861567	-0.335225	2.914004
C	1.189387	0.485466	4.141893
C	1.526574	1.592559	3.140004
H	2.076111	1.178807	2.285244
H	2.166169	2.342128	3.615264
H	0.623044	2.087616	2.776773
C	0.473370	1.054681	5.368208
H	-0.452365	1.558617	5.080665
H	1.119435	1.777137	5.877601
H	0.231035	0.253145	6.074376
C	2.477934	-0.215230	4.568889
H	2.271452	-1.032326	5.269675
H	3.140318	0.497763	5.067262
H	3.010966	-0.625504	3.703153
N	-3.413125	2.445873	0.085679
H	-3.631900	2.203988	1.043689
N	-1.916698	0.633604	0.277789
N	-1.379917	-1.439520	2.301458
N	0.376022	-0.549407	3.471012
H	0.560367	-1.496814	3.779168
O	-2.216389	1.761132	-1.740663
O	-1.427681	0.774248	2.908460
C	3.953738	0.656415	-1.414465
H	3.985311	1.378232	-2.223664
C	5.127192	0.225253	-0.816969
O	6.361203	0.634572	-1.167044
C	5.059855	-0.711535	0.255949
O	6.234002	-1.063583	0.810669
C	3.824013	-1.196723	0.652687
H	3.751510	-1.938109	1.441494
C	2.646109	-0.744935	0.050110
C	0.953564	-2.551466	0.133592
C	0.606062	-4.046363	-1.855227
C	1.064900	-5.357489	-1.217004
H	2.158189	-5.421181	-1.212842
H	0.672909	-6.201724	-1.793151
H	0.703947	-5.441299	-0.189405
C	-0.920778	-3.928827	-1.840002
H	-1.310870	-3.965854	-0.819426
H	-1.362068	-4.753347	-2.408690
H	-1.239889	-2.985824	-2.298248
C	1.126339	-3.953930	-3.286952
H	0.816054	-3.015459	-3.761661
H	0.724315	-4.779228	-3.880761
H	2.220120	-4.018192	-3.312966
N	1.768065	2.860269	-0.605325
H	2.114386	2.329137	0.182262
N	1.490532	0.718112	-1.544184
N	1.381466	-1.236553	0.503733
N	1.192644	-2.891440	-1.141969
H	1.739734	-2.249149	-1.698431
O	0.989713	2.630351	-2.754330
O	0.351434	-3.242556	0.957293
C	2.273846	4.617812	0.945301
H	1.631648	4.170820	1.711687
H	2.297192	5.697378	1.115188
H	3.293124	4.233800	1.069946
C	0.303856	4.828213	-0.585282
H	-0.109227	4.592266	-1.568874
H	0.270724	5.913061	-0.445159
H	-0.331229	4.364009	0.177131
C	2.654614	4.975038	-1.501351
H	3.679027	4.599124	-1.402227
H	2.672239	6.059383	-1.355040
H	2.302454	4.768984	-2.514863
C	1.743499	4.330305	-0.456032
C	1.402648	2.176286	-1.699265
C	2.712023	0.193455	-0.970205
C	-5.568329	-1.458581	-2.569032
H	-4.662951	-1.291407	-3.167065
H	-5.942090	-0.499462	-2.186896
H	-6.334013	-1.923117	-3.192237
C	-4.585216	-5.144838	1.359038
H	-3.539120	-5.474589	1.312040
H	-4.812377	-4.783486	2.370089
H	-5.242220	-5.981162	1.114847
C	6.200507	-1.955930	1.928403
H	5.616848	-1.527856	2.753394
H	7.238994	-2.076156	2.238720
H	5.785648	-2.930698	1.644710
C	6.471706	1.588404	-2.226978
H	7.539931	1.776248	-2.341156
H	5.960002	2.524474	-1.968883
H	6.065394	1.186856	-3.163289
H	1.347975	0.361341	-2.491618
H	1.293933	-1.156935	1.519168
H	-1.800145	0.970314	1.259489
H	-0.764189	-2.249348	2.220165

10. References.

(1) Mader, E. A.; Davidson, E. R.; Mayer, J. M. Large Ground-State Entropy Changes for Hydrogen Atom Transfer Reactions of Iron Complexes. *Journal of the American Chemical Society* **2007**, *129* (16), 5153-5166. DOI: 10.1021/ja0686918. De Vries, J. G.; Kellogg, R. M. Reduction of aldehydes and ketones by sodium dithionite. *The Journal of Organic Chemistry* **1980**, *45* (21), 4126-4129. DOI: 10.1021/jo01309a011.
(2) Manner, V. W.; Markle, T. F.; Freudenthal, J. H.; Roth, J. P.; Mayer, J. M. The first crystal structure of a monomeric phenoxyl radical: 2,4,6-tri-tert-butylphenoxyl radical. *Chemical communications* **2008**, (2), 256-258, 10.1039/B712872J. DOI: 10.1039/B712872J.
(3) Wittman, J. M.; Hayoun, R.; Kaminsky, W.; Coggins, M. K.; Mayer, J. M. A C–C Bonded Phenoxyl Radical Dimer with a Zero Bond Dissociation Free Energy. *Journal of the American Chemical Society* **2013**, *135* (35), 12956-12959. DOI: 10.1021/ja406500h.
(4) Wise, C. F.; Agarwal, R. G.; Mayer, J. M. Determining Proton-Coupled Standard Potentials and X–H Bond Dissociation Free Energies in Nonaqueous Solvents Using Open-Circuit Potential Measurements. *Journal of the American Chemical Society* **2020**, *142* (24), 10681-10691. DOI: 10.1021/jacs.0c01032.
(5) Wu, T.; Rajabimoghadam, K.; Puri, A.; Hebert, D. D.; Qiu, Y. L.; Eichelberger, S.; Siegler, M. A.; Swart, M.; Hendrich, M. P.; Garcia-Bosch, I. A 4H+/4e– Electron-Coupled-Proton Buffer Based on a Mononuclear Cu Complex. *Journal of the American Chemical Society* **2022**, *144* (37), 16905-16915. DOI: 10.1021/jacs.2c05454.
(6) Potentiometry in Non-Aqueous Solutions. In *Electrochemistry in Nonaqueous Solutions*, 2002; pp 167-200.
(7) Maran, F.; Celadon, D.; Severin, M. G.; Vianello, E. Electrochemical determination of the pKa of weak acids in N,N-dimethylformamide. *Journal of the American Chemical Society* **1991**, *113* (24), 9320-9329. DOI: 10.1021/ja00024a041.
(8) *ADF2012.01*; SCM: Amsterdam, 2012. (accessed.
(9) te Velde, G.; Bickelhaupt, F. M.; Baerends, E. J.; Fonseca Guerra, C.; van Gisbergen, S. J. A.; Snijders, J. G.; Ziegler, T. Chemistry with ADF. *J Comput Chem* **2001**, *22* (9), 931-967.
(10) Swart, M.; Bickelhaupt, F. M. QUILD: QUantum-regions interconnected by local descriptions. *J Comput Chem* **2008**, *29* (5), 724-734. DOI: 10.1002/jcc.20834.
(11) van Lenthe, E.; Baerends, E. J. Optimized Slater-Type Basis Sets for the Elements 1–118. *J Comput Chem* **2003**, *24*, 1142-1156. Chong, D. P.; van Lenthe, E.; van Gisbergen, S. J. A.; Baerends, E. J. Even-tempered Slater-type orbitals revisited: from hydrogen to krypton. *J Comput Chem* **2004**, *25*, 1030-1036.
(12) Wolff, S. K. Analytical second derivatives in the Amsterdam density functional package. *International Journal of Quantum Chemistry* **2005**, *104*, 645-659.
(13) Becke, A. D. Density-functional exchange-energy approximation with correct asymptotic behavior. *Physical Review A* **1988**, *38*, 3098-3100. Perdew, J. P. Density-functional approximation for the correlation-energy of the inhomogeneous electron-gas. *Physical Review B* **1986**, *33*, 8822-8824. Erratum: *Ibid*. 8834, 7406. Grimme, S.; Antony, J.; Ehrlich, S.; Krieg, H. A consistent and accurate ab initio parametrization of density functional dispersion correction (DFT-D) for the 94 elements H-Pu. *Journal of Chemical Physics* **2010**, *132*, 154104.
(14) Klamt, A.; Schüürmann, G. COSMO: a new approach to dielectric screening in solvents with explicit expressions for the screening energy and its gradient. *Journal of the Chemical Society Perkin Transactions 2* **1993**, 799-805.
(15) Swart, M.; Rösler, E.; Bickelhaupt, F. M. Proton Affinities in Water of Maingroup-Element Hydrides. Effects of Hydration and Methyl Substitution. *Eur. J. Inorg. Chem.* **2007**, 3646-3654.
(16) Averkiev, B. B.; Truhlar, D. G. Free energy of reaction by density functional theory: oxidative addition of ammonia by an iridium complex with PCP pincer ligands. *Catalysis Science & Technology* **2011**, *1*, 1526-1529. DOI: 10.1039/c1cy00227a. Klein, J. E. M. N.; Dereli, B.; Que Jr., L.; Cramer, C. J. Why metal–oxos react with dihydroanthracene and cyclohexadiene at comparable rates, despite having different C–H bond strengths. A computational study. *Chem. Commun.* **2016**, *52*, 10509-10512. DOI: 10.1039/c6cc05395e.
(17) van Lenthe, E.; Baerends, E. J.; Snijders, J. G. Relativistic regular two-component Hamiltonians. *Journal of Chemical Physics* **1993**, *99*, 4597-4610.
(18) Álvarez-Moreno, M.; de Graaf, C.; López, N.; Maseras, F.; Poblet, J. M.; Bo, C. Managing the Computational Chemistry Big Data Problem: The ioChem-BD Platform. *Journal of Chemical Information and Modeling* **2015**, *55* (1), 95-103. DOI: 10.1021/ci500593j.

(19) Wilkinson, M. D.; Dumontier, M.; Aalbersberg, I. J.; Appleton, G.; Axton, M.; Baak, A.; Blomberg, N.; Boiten, J.-W.; da Silva Santos, L. B.; Bourne, P. E.; et al. The FAIR Guiding Principles for scientific data management and stewardship. *Scientific Data* **2016**, *3* (1), 160018. DOI: 10.1038/sdata.2016.18.
(20) Namazian, M.; Lin, C. Y.; Coote, M. L. Benchmark Calculations of Absolute Reduction Potential of Ferricinium/Ferrocene Couple in Nonaqueous Solutions. *Journal of Chemical Theory and Computation* **2010**, *6* (9), 2721-2725. DOI: 10.1021/ct1003252.
(21) Bartmess, J. E. Thermodynamics of the Electron and the Proton. *The Journal of Physical Chemistry* **1994**, *98* (25), 6420-6424. DOI: 10.1021/j100076a029.
(22) Souilah, C.; Jannuzzi, S. A. V.; Demirbas, D.; Ivlev, S.; Swart, M.; DeBeer, S.; Casitas, A. Synthesis of FeIII and FeIV Cyanide Complexes Using Hypervalent Iodine Reagents as Cyano-Transfer One-Electron Oxidants. *Angew. Chem., Int. Ed.* **2022**, *61* (22), e202201699. DOI: https://doi.org/10.1002/anie.202201699.
(23) Cattaneo, M.; Parada, G. A.; Tenderholt, A. L.; Kaminsky, W.; Mayer, J. M. Structural, Electronic, and Thermochemical Preference for Multi-PCET Reactivity of Ruthenium(II)-Amine and Ruthenium(IV)-Amido Complexes. *Eur. J. Inorg. Chem.* **2021**, *2021* (39), 4066-4073. DOI: https://doi.org/10.1002/ejic.202100604.
(24) Kelly, C. P.; Cramer, C. J.; Truhlar, D. G. Adding Explicit Solvent Molecules to Continuum Solvent Calculations for the Calculation of Aqueous Acid Dissociation Constants. *The Journal of Physical Chemistry A* **2006**, *110* (7), 2493-2499. DOI: 10.1021/jp055336f.
